# Strategies for implementing genomic selection in a public soybean breeding program

**DOI:** 10.1371/journal.pone.0353481

**Published:** 2026-07-13

**Authors:** Leonardo De Azevedo Peixoto, Eliana Monteverde Dominguez, Andrew Scaboo, Reka Howard, Asheesh K. Singh

**Affiliations:** 1 Department of Agronomy, Iowa State University, Ames, Iowa, United States of America; 2 Department of Crop Sciences, University of Illinois, Urbana, Illinois, United States of America; 3 Division of Plant Science and Technology, University of Missouri, Columbia, Missouri, United States of America; 4 Department of Statistics, University of Nebraska–Lincoln, Lincoln, Nebraska, United States of America; Jeju National University, KOREA, REPUBLIC OF

## Abstract

Improving selection accuracy in soybean breeding programs is crucial for reducing costs and shortening the time required to develop new varieties. Genomic selection (GS) is a promising tool for enhancing the accuracy of selection. This study aimed to identify the most effective GS models for predicting key traits, including seed yield, protein content, oil content, and maturity in soybean breeding programs. Additionally, we sought to determine the optimal training population optimization method (structure vs. size) and establish the minimum number of genotypes required for a training population that ensures high model performance across locations. Finally, we explored multitrait selection based on genomic prediction to improve breeding decisions. Data were obtained from the soybean variety development program and included experiments planted in a randomized block design with two replications at eight locations during the 2023 and 2024 growing seasons. Six GS models were tested: rrBLUP, Bayes A, Bayes B, RKHS, random forest, and support vector machine. Four methods for optimizing the training population were evaluated: random selection (RS), maturity group random selection (MGRS), experimental random selection (ERS), and genetic algorithm (GA). We also assessed ten different training population sizes (ranging from 10% to 90%) and five selection index strategies: direct selection on seed yield, direct selection on oil content, direct selection on protein content, Rank-index, and Smith-Hazel index. Our results suggest that the most effective strategy for implementing GS in a public soybean breeding program involves using the rrBLUP model for genomic prediction. To optimize its performance, it is recommended to train the model using 80% of the total population. This approach provides a robust and reliable prediction. Furthermore, structuring the training population through experimental random selection enhances genetic diversity, which is crucial for improving selection accuracy and robustness across breeding cycles. Finally, the Rank-index proved to be a highly effective strategy for selecting soybean genotypes, particularly for improving seed yield and oil content, while results for seed protein content were less promising. By considering multiple traits simultaneously, this method offers a more balanced approach to genetic improvement compared to single-trait selection methods, making it an excellent tool for breeding programs that aim to enhance both productivity and quality.

## Introduction

Traditional soybean breeding methods rely heavily on phenotypic selection, which is crucial for informed decision-making [1]. However, phenotype-only selection presents important limitations, particularly for traits with low heritability, as the observed phenotype is strongly influenced by environmental variability, reducing the accuracy of selection based solely on field observations. As a result, improving complex traits such as seed yield, protein content, and oil composition often requires extensive multi-environment field trials, making the breeding process time-consuming and resource-intensive [2]. An alternative approach is genomic selection (GS), which uses genome-wide molecular marker information to predict the genetic potential of individuals before phenotypic traits are fully expressed [1–3]. By leveraging genomic information, GS can increase selection accuracy for polygenic traits and reduce the number of field evaluation cycles required, thereby accelerating genetic gain. Despite these advantages, the implementation of GS in breeding programs, particularly public breeding programs, may still be constrained by factors such as genotyping costs, the need for well-structured training populations, and the computational and statistical expertise required to implement genomic prediction models [4].

Soybean breeding programs focus on several key traits, including improving seed yield, enhancing resistance to pests and diseases, and increasing tolerance to abiotic stresses such as drought [5,6]. GS has emerged as a transformative approach in plant breeding, offering a powerful method to enhance the efficiency and accuracy of selecting superior crop varieties [7]. Soybean [*Glycine max* (L.) Merr.], a vital oilseed crop with significant economic and nutritional importance, benefits significantly from integrating GS into its breeding programs [8]. This approach utilizes dense molecular markers distributed across the genome to predict the breeding values of plants, thereby accelerating the breeding cycle and enhancing selection precision [9]. The integration of GS for trait prediction and selection holds significant promise for addressing these challenges by enabling the simultaneous improvement of multiple traits [10]. Moreover, GS facilitates the utilization of a broader genetic base, thereby increasing the potential for genetic gains [11].

Several studies have demonstrated the effectiveness of GS in soybean breeding [12–14]. Notable improvements have been observed in traits such as seed yield [12], protein content [15], and resistance to pathogens [16], supported by advancements in high-throughput genotyping and the development of sophisticated statistical models capable of managing large genomic datasets [17]. Techniques such as ridge regression best linear unbiased prediction (RR-BLUP) [12], Bayesian approaches like Bayes A and Bayes B [18], and kernel-based methods such as reproducing kernel Hilbert spaces (RKHS) [19] have shown significant promise in GS for soybean. However, several factors can influence the predictive ability estimated by GS models in the soybean breeding program, such as training population structure [20], training population size [21], number of markers [22], GS methods [22,23], and number of locations [18].

The composition of the training population (TR) is a critical factor influencing the accuracy of genomic predictions. Larger TRs tend to improve predictive ability [24], but simply increasing size is not always optimal. Low genetic diversity in the TR limits the accuracy of estimating genotypic effects [25], while excessive diversity, especially from individuals genetically distant from the target population (TE), can reduce predictive ability [26]. Thus, striking the right balance between diversity and relatedness is essential. Various strategies have been developed to optimize TR composition [27,28] and trait-specific or bin-weighted relationship matrices [29,30]. However, these methods often depend on population structure and trait architecture. In this context, genetic algorithms (GAs) have gained attention for their flexibility and efficiency in optimizing TRs [31]. By mimicking natural selection through iterative processes such as selection, crossover, and mutation, GAs effectively explore complex solution spaces to identify optimal subsets. Studies have shown that GAs can outperform traditional approaches in selecting training sets that maximize predictive ability [32–34].

Another important aspect to consider in plant breeding is that selection is rarely based on a single trait. In most cases, breeders must consider multiple traits of interest simultaneously. One widely used method for multi-trait selection is the selection index [35]. This approach integrates the goals of a breeding program and the desired response to selection across traits into a single index, helping breeders identify genotypes with the highest overall merit [36,37]. Selection indices are particularly useful when dealing with complex trade-offs, such as the well-known negative correlation between oil and protein content in soybean [38]. Improving both traits simultaneously is a significant challenge, as lines with higher oil content typically have lower protein content. Selection indices can simplify decision-making by converting multiple trait values into a single, interpretable metric. However, defining the appropriate economic weights for each trait remains a challenge. These weights may be based on market prices, breeding priorities, or specific program objectives [1,39,40].

In this paper, we present the above-listed factors and their combinations, which are crucial for selecting the most appropriate GS strategy to improve predictive ability in a public soybean breeding program. The phenotypic data used for genomic prediction were obtained through standard field evaluations commonly used in soybean breeding programs. Seed yield was measured at harvest using plot-level yield data, while relative maturity was recorded based on the timing of plant development and physiological maturity. Seed composition traits, including protein and oil content, were determined using seed quality analyses performed with near-infrared spectroscopy (NIR).

Therefore, the objectives of this research were: (a) to identify the most suitable GS models for predicting seed yield, protein content, oil content, and maturity in soybean breeding programs, while determining the optimal training population strategy (structure versus size) and establishing the minimum number of genotypes required to build a training population that ensures high predictive performance across multiple locations; and (b) to perform multi-trait selection based on genomic prediction to improve the accuracy of breeding decisions. By addressing these objectives, we aim to enhance the application of GS in soybean breeding.

## Materials and methods

### Experimental design

At the Iowa State University soybean breeding program, approximately 75 new breeding populations are developed annually. Generations are advanced using a modified single-seed (pod) descent method, in which individual plant selection is conducted in the F₂ generation. From each selected plant, a single pod is harvested and bulked, and the resulting seed is advanced in a winter nursery. Upon return, the bulked seed is planted in Iowa, where selection is performed based on maturity group, plant height, pod and node placement, and overall plant health. Selected plants are advanced to the next generation and evaluated as progeny rows in a single-replication paired-row design. For this study, F₆-derived lines were used, as they are considered to be homozygous and suitable for reliable phenotypic evaluation.

Experiments were conducted during the 2023 and 2024 growing seasons. Detailed information for each experimental location, including the number of genotypes, number of replications, harvest date, plot length, alley length, number of rows, row spacing, planter type, fertilizer management, chemical applications, and harvester type, is provided in S1 Table.

### Data collection

Seed yield was measured using a plot combine (Zurn 150 Plot Combine, for the weight/moisture/real-time NIR, we used a HarvestMaster H3 GrainGuage) equipped with sensors for weight and moisture content. Seed yield was then calculated based on the known plot dimensions, which were 12’ planted, 4’ alley, 2 rows, 30” row spacing. Maturity was assessed visually. During early-stage testing, relative maturity was determined using standard check cultivars grown within each field. Seed oil and protein content were estimated in real time using a near-infrared (NIR – PerkinElmer DA 7250 GP NIR ANALYZER) sensor mounted on the combine, and these estimates were validated using a bench-top analyzer at the Ames facility. Seed yield was recorded for all experiments in both years. Maturity data were collected at four locations (Ames, Lucas, Sutherland, and Crawfordsville), while protein and oil content data were collected at three locations (Ames, Lucas, and Crawfordsville) each year.

Outliers were detected using boxplots and standardized residuals greater than ±3 standard deviations from the mean [41]. Observations meeting these criteria were considered likely errors or inconsistencies and were removed prior to downstream analyses. The proportion of excluded observations differed among traits, locations, and years. A detailed breakdown of the percentage of outliers detected for each trait across environments is presented in S2 Table.

Because the proportion of removed observations was low, the impact on variance components and the distribution of genomic estimated breeding values (GEBVs) is expected to be minimal. Moreover, genomic prediction models are generally robust to the removal of clear data anomalies when these are identified using conservative statistical thresholds. Best Linear Unbiased Predictors (BLUPs) for each genotype were estimated using the following linear mixed-effects model:


$$\begin{array}{c} y_{ijk}=\mu +B_{k}+G_{i}+E_{j}+{GE}_{ij}+\epsilon_{ijk} \end{array}$$

where:

$y_{ijk}$ is the phenotypic observation for genotype i, environment j, and replication k;

$B_{k}$ is the fixed effects of block k;

$G_{i}$ is the random effect of genotype i with assumption Gj ~ N(0,$\overset{\text{2}}{\underset{g}{\sigma }}$);

$E_{j}$ is the random effect of environment j with assumption Ej ~ N(0,$\overset{\text{2}}{\underset{E}{\sigma }}$);

${GE}_{ij}$ is the interaction effect between genotype i and environment j, which is considered random;

$\epsilon_{ijk}$ is the random error term with assumption Ej ~ N(0,σ^2^).

All the mixed-model analyses were performed using the lme4 package [42] in R. The BLUPs were estimated based on the mixed-model outcomes for each trait, which were used as input for each GS method.

### Genomic selection methods

Six GS methods were compared when predicting seed yield, protein content, oil content, and maturity in the Iowa State University’s soybean breeding program. The six methods used were Ridge Regression Best Linear Unbiased Prediction (rrBLUP) [9], Random Forest (RF) [43], Support Vector Machine (SVM) [43], Reproducing Kernel Hilbert Space (RKHS) [44], Bayes A [9], and Bayes B [9].

A total of approximately 3,000 single-nucleotide polymorphism (SNP) markers were used to construct genomic prediction models. Prior to analysis, the marker dataset was subjected to quality control filtering to ensure reliable genotype information. Markers were filtered based on call rate and minor allele frequency (MAF). Specifically, SNPs with a call rate of less than 90% were removed to minimize the impact of missing genotype data, and markers with an MAF of less than 5% were excluded to avoid including rare alleles that provide limited information for genomic prediction. After filtering, the remaining high-quality SNP markers (1,326) were used to build genomic relationship matrices and train GS models to predict seed yield, protein content, oil content, and maturity in the Iowa State University soybean breeding program.

All genomic prediction methods were evaluated using a repeated 5-fold cross-validation scheme with 10 independent replications per environment–year combination, yielding a total of 384,000 model fits. For each cross-validation replicate, the data were partitioned into five mutually exclusive subsets: four for training and one for validation. This procedure was repeated across all folds and replications to ensure robust estimation of predictive ability. GS models were fitted independently within each specific environment and year, thereby restricting inference to within-environment prediction scenarios. Genotype × environment (G × E) interaction effects were not explicitly modeled in this study, as the objective was to assess model performance under environment-specific prediction frameworks.

All methods were implemented in R. The package BGLR [45] was used to implement Bayes A, Bayes B, and RKHS. The package rrBLUP [46] was used for rrBLUP, and the package caret [47] was used for RF and SVM.

### Training population structure

Four training population structures were evaluated to determine the configuration that provided the highest prediction accuracy.

The first strategy was Random Selection (RS), in which genotypes were randomly selected from the entire population to compose the training set. The second strategy was Maturity Group Random Selection (MGRS), in which the training population was constructed via stratified random sampling by maturity group, ensuring that each group's relative frequency in the training set matched its proportion in the entire population. The third strategy was Experiment Random Selection (ERS), in which genotypes were randomly sampled within each experiment while preserving the same proportional distribution observed in the complete dataset. Finally, a Genetic Algorithm (GA) approach was implemented to identify a training population with maximum genetic diversity. The GA simulates natural selection by iteratively generating candidate genotype subsets, applying crossover and mutation operators, and retaining the best-performing subsets across generations. Genetic diversity was quantified as the average pairwise genetic distance among selected individuals, calculated from the marker-based genomic relationship matrix (GRM), and the subset with the highest diversity score was retained as the final training population.

### Training population size

Genomic prediction performance was evaluated across training population sizes ranging from 10% to 90% of the available genotypes (10, 20, 30, 40, 50, 60, 70, 80, and 90%). For each location and year, the complete set of evaluated genotypes was partitioned into training and testing subsets according to the four training population selection strategies described above. The training set (10–90% of genotypes) was used to fit the genomic prediction model, and the remaining genotypes were used as the testing set. Predictive ability was then assessed by correlating predicted and observed phenotypes for the withheld genotypes.

### Genomic selection effectiveness

The following statistical parameters were estimated to compare all evaluated scenarios, including the combinations of training population structure, training population size, and GS methods described above. Predictive ability was calculated as the Pearson correlation coefficient between the observed phenotypic values in the validation population and the corresponding genomic estimated breeding values (GEBV). In addition, validation coincidence was calculated as the percentage overlap between the top 10% of individuals selected based on GEBV in the validation population and the top 10% selected based on phenotypic values in the same population.

### Statistical analysis

Plateau regression [48] was used to determine the optimal number of genotypes to be evaluated in each location-year combination, without incurring any loss of prediction accuracy.

A three-way ANOVA was conducted to assess the effects of GS method, training population structure, and training population size on prediction accuracy. All effects in the model were treated as fixed, and the analysis followed the model:


$$\begin{array}{c} {\text{Accuracy}}_{ijkl}=\mu +M_{i}+T_{j}+S_{k}+{MT}_{ij}+{MS}_{ik}+{TS}_{jk}+{MTS}_{ijk}+\varepsilon_{ijkl} \end{array}$$

where

$M_{i}$ represents the genomic selection method,

$T_{j}$ the training population structure,

$S_{k}$ the training population size,

and $\varepsilon_{ijkl}$ the residual error.

Tukey’s HSD test was applied for post-hoc pairwise comparisons among factor levels. All analyses were performed using R, and the following packages were used: AgroReg [49] for plateau regression, easyanova [50] for ANOVA and Tukey test, and ggplot2 [51] for all graphics. All R scripts used in this study are publicly available at Figshare: https://figshare.com/s/028790bf3c927875528a.

### Optimal number of locations to achieve maximum accuracy

A power analysis [52] was conducted to determine the optimal number of locations required to achieve predictive abilities above a predefined threshold for seed yield, protein content, oil content, and maturity. The parameters used in the analysis, including genetic variance, genotype-by-environment interaction variance, residual variance, and heritability, were estimated for each trait using mixed models fitted to the multi-environment trial data. These variance components were then used to approximate the expected reliability of genotype evaluation across environments and to derive the expected correlation between predicted and true genetic values as a function of the number of testing locations, assuming balanced replication across environments.

To evaluate different levels of genetic diversity within the population, genotypes were grouped into two to five clusters representing distinct performance categories: (1) Elite Performers, (2) High Performers, (3) Average Performers, (4) Low Performers, and (5) Underperformers. The clustering of genotypes into these groups was performed using the k-means algorithm based on trait performance. These clusters were used to simulate scenarios with varying genetic structures, allowing the power analysis to evaluate how the number of testing locations influences the expected prediction accuracy under different levels of genetic variability.

### Index selection implementation

After evaluating all GS models and identifying the best-performing approach, genomic estimated breeding values (GEBVs) were predicted for all experimental lines. From this population, the superior 10% of genotypes (selection intensity = 10%) were selected using five different strategies: (1) direct selection for seed yield only, (2) direct selection for oil content only, (3) direct selection for protein content only, (4) a rank-based index [53], and (5) the Smith–Hazel index [54,55]. For the Smith–Hazel index, the weights were defined using the genetic standard deviation of each trait, which provides a practical approach to balance traits measured on different scales while accounting for their underlying genetic variability. Although alternative weighting strategies based on economic values or breeding priorities could also be applied, the Smith-Hazel index was chosen for this study due to its scale-independent combination of traits in a multi-trait selection scenario.

Response to selection was calculated for seed yield, protein content, and oil content across the five selection methods using the formula described by Singh et al. [1]:


$$\begin{array}{c} \Delta G=\overset{―}{x_{s}}-\overset{―}{x_{g}} \end{array}$$

where

ΔG is the expected genetic gain,

$\overset{―}{x_{s}}$ is the selected mean,

$\overset{―}{x_{g}}$ is the general mean.

Additionally, we evaluated the overall response by considering the simultaneous improvement of the three traits. Specifically, we compared the five selection strategies described above in terms of their ability to maximize genetic gain across seed yield, protein content, and oil content, and identified the most effective approach.

All selection index analyses were performed using R (All R scripts used in this study are publicly available at Figshare: https://figshare.com/s/028790bf3c927875528a).

## Results

### Optimal number of genotypes for training genomic selection models

**Fig 1:**
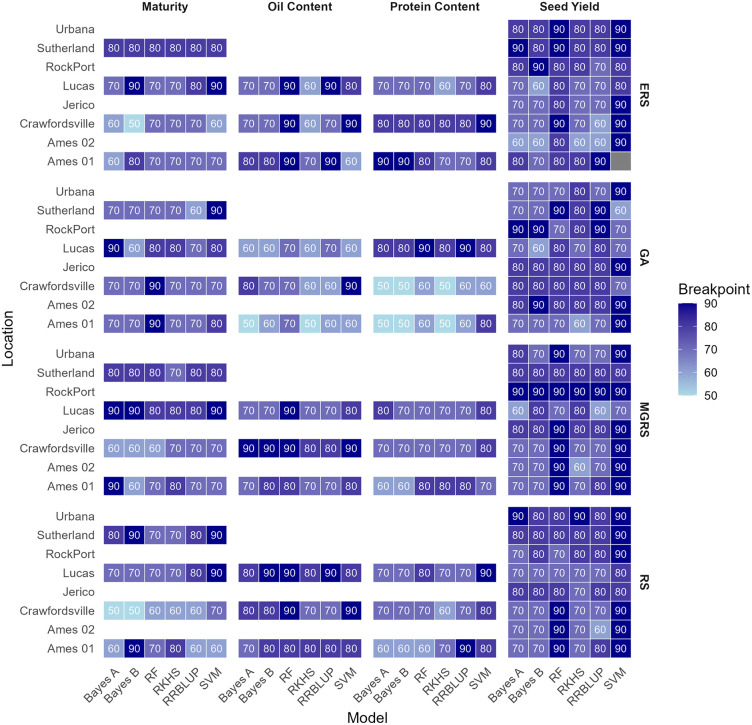
Minimum percentage of genotypes required in the training population to achieve maximum predictive ability for seed yield, oil content, protein content, and maturity across different training population structures, genomic selection methods, and locations. ERS – Experiment random selection, GA – Genetic algorithm, MGRS – Maturity group random selection, RS – Random selection. Six genomic selection models were tested, including Bayes A, Bayes B, Random Forest (RF), Reproducing Kernel Hilbert Space (RKHS), Ridge Regression Best Linear Unbiased Prediction (rrBLUP), and Support Vector Machine (SVM).

The minimum number of genotypes (MNG) required to achieve the highest predictive ability at each location was determined using plateau regression, and the corresponding regression parameters are presented in S3 Table. Across traits, models, and training population structures, the estimated breakpoints generally ranged between 50% and 90% of the total genotypes, indicating that a substantial proportion of individuals must be included in the training population to achieve maximum predictive accuracy (Fig 1). For seed yield, the breakpoints ranged from approximately 58% to 84% across models and training population structures, with lower values typically observed under the experimental and mixed-group structures and higher values under the genetic algorithm approach. For oil content, the breakpoints were generally concentrated between 56% and 88%, while protein content required slightly larger training populations in several scenarios, with breakpoints reaching up to 87%. In contrast, maturity showed relatively smaller breakpoints in most cases, typically ranging between 53% and 68% of the genotypes. Despite these differences among traits and prediction models, the results consistently indicate that training populations comprising approximately 60–80% of the available genotypes are sufficient to achieve near-maximum predictive ability across most scenarios.

Based on the results of the plateau regression analysis, we determined the optimal number of genotypes to evaluate at each location for effective training of the genomic prediction model. These findings provide valuable insights for refining the breeding strategy, such as increasing the number of testing locations or expanding the number of genotypes evaluated per site.

### Comparing the performance of genomic selection models

Statistically, the Random Forest (RF) and Support Vector Machine (SVM) methods often achieved the highest predictive abilities for seed yield across locations and training population structures (TPS) (S1–S4 Figs). For protein content, SVM consistently showed the highest predictive ability across locations and TPS, followed closely by RF (S1–S4 Figs). Similarly, for oil content, both SVM and RF frequently produced the highest prediction accuracies across locations and TPS configurations (S1–S4 Figs). For maturity, RF outperformed the other methods in Lucas and Sutherland, while showing similar performance to SVM in Ames 01 and Crawfordsville (S1–S4 Figs). However, the differences in predictive ability among methods were generally small across traits, locations, and TPS scenarios. In most cases, rrBLUP showed predictive abilities comparable to those of machine learning approaches, indicating that the overall performance gap among models was limited. Given its stable performance across environments, computational efficiency, and widespread use in plant breeding programs, rrBLUP remains a practical and robust genomic prediction method despite the marginal numerical advantages occasionally observed for RF and SVM.

### Comparative analysis of training population structure

**Fig 2:**
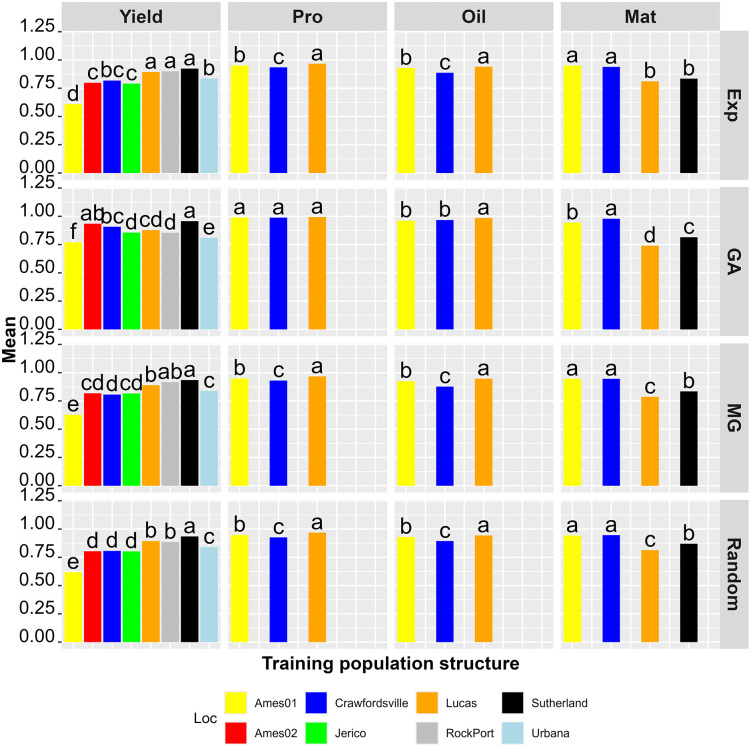
Predictive ability across all locations for seed yield, protein content (Pro), oil content, and maturity (Mat) under four training-population structures: Random (random selection), MG (maturity-group random selection), Exp (experimental random selection), and GA (genetic algorithm), using the rrBLUP genomic selection model. Locations followed by different letters differ significantly according to Tukey’s test at the 5% significance level.

Lucas, Rockport, and Sutherland consistently exhibited the highest predictive ability for seed yield (Fig 2 and S5–S9 Figs), demonstrating distinct mean values compared to other locations. Meanwhile, Lucas presented the best predictive ability for oil content (Fig 2 and S5–S9 Figs), for protein content (Fig 2 and S5–S9 Figs), and Ames 01 and Crawfordsville for maturity (Fig 2 and S5–S9 Figs). This suggests potential site-specific effects. In contrast, Ames 01 showed the lowest predictive ability for seed yield (Fig 2 and S5–S9 Figs), Crawfordsville for oil and protein content (Fig 2 and S5–S9 Figs), and Lucas for maturity (Fig 2 and S5–S9 Figs), highlighting the influence of locations on prediction outcomes across various traits.

**Fig 3:**
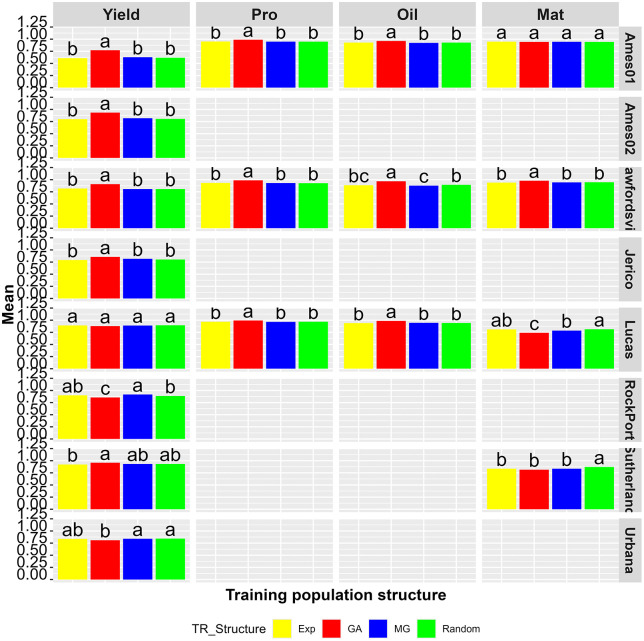
Predictive ability across all training population structures for seed yield, protein content (Pro), oil content, and maturity (Mat) under eight locations: Ames 01, Ames 02, Crawfordsville, Jerico, Lucas, Rockport, Sutherland, and Urbana, using the rrBLUP genomic selection model. Training population structures followed by different letters differ significantly according to Tukey’s test at the 5% significance level.

Building the training population based on the genetic algorithm was the most effective strategy for improving seed yield predictive ability in almost all locations and across GS models (Fig 3 and S10–S14 Figs). However, for Urbana and Rockport, identifying the Maturity Group random selection (MGRS) structure was the most effective across GS models (Fig 3 and S10–S14 Figs).

In addition, the predictive ability for oil and protein content was highest for the genetic algorithm training population structure across all locations and GS models (Fig 3 and S10–S14 Figs). For maturity, the random selection strategy achieved the highest accuracy for Sutherland, Lucas, and Ames 01 (Fig 3 and S10–S14 Figs).

### Optimal number of locations to enhance the best prediction accuracy

The genetic parameters used for power analysis are presented in Table 1. These parameters were estimated from data collected at 15 locations for both 2023 and 2024 and served as the basis for the simulations performed in this study. The results indicate substantial differences among traits in terms of variance components and heritability. Seed yield presented the largest residual variance (σ² = 21.28) and a relatively high genotype × environment interaction variance ($\overset{\text{2}}{\underset{GE}{\sigma }}$ = 10.20), resulting in a lower broad-sense heritability (h² = 0.30). This suggests that seed yield is strongly influenced by environmental conditions and G × E interactions, which can make accurate prediction more challenging. In contrast, oil and protein content showed much higher heritability estimates (h² = 0.89 and 0.94, respectively), indicating that these traits are more strongly controlled by genetic factors and are less affected by environmental variation. Maturity also exhibited high heritability (h² = 0.83), with relatively small residual and G × E variances compared to the genetic variance. These differences in genetic architecture among traits help explain the variation observed in the number of locations required to achieve high predictive ability in the subsequent power analysis.

**Table 1:** Genetic parameters for the four traits were evaluated at eight locations. $\overset{2}{\underset{G}{\sigma }}$*-* genetic variance, $\overset{2}{\underset{GE}{\sigma }}$ – geneticXenvironment variance, $\sigma^{2}$- residual variance, $H^{2}$- heritability.

Trait	$\overset{2}{\underset{G}{\sigma }}$	$\overset{2}{\underset{GE}{\sigma }}$	$\sigma^{2}$	$H^{2}$
**Seed yield**	9.17	10.20	21.28	0.30
**Oil content**	0.32	0.04	0.04	0.89
**Protein content**	1.09	0.17	0.07	0.94
**Maturity**	0.15	0.02	0.01	0.83

**Fig 4:**
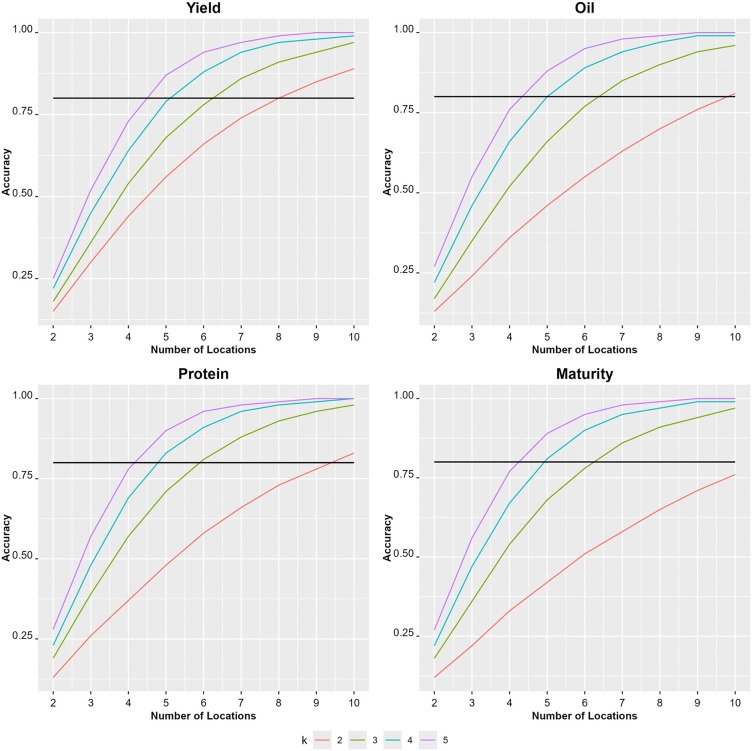
Number of locations needed to get predictive ability greater than 0.8 for seed yield, oil content, protein content, and maturity.

The power analysis revealed that the number of locations required to achieve predictive abilities greater than 0.80 varied depending on the level of genetic stratification in the population (Fig 4). When genotypes were divided into two groups, at least eight locations were required to achieve the target predictive ability for seed yield, ten for oil and protein content, and more than ten for maturity. When the population was partitioned into three groups, the required number of locations decreased to seven for seed yield, oil content, and maturity, and six for protein content. For four genotype groups, six locations were sufficient for seed yield, while five locations were required for oil content, protein content, and maturity. When genotypes were further divided into five groups, only five locations were required to achieve predictive abilities above 0.80 for all evaluated traits. These results indicate that increasing genetic stratification within the population improves the efficiency of prediction across environments, thereby reducing the number of testing locations needed to achieve high predictive accuracy.

### Selection index implementation

**Fig 5:**
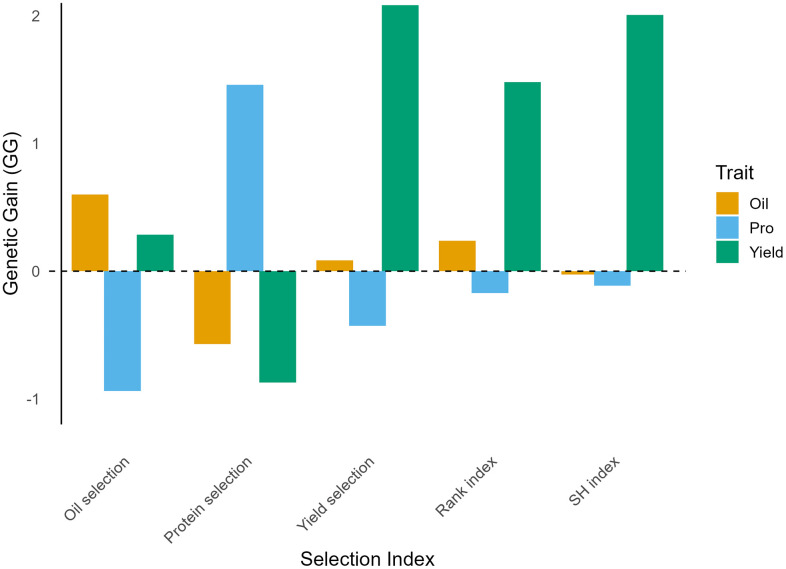
Genetic gain estimates for seed yield, oil content, and protein content obtained through five selection strategies: direct selection for seed yield, direct selection for oil content, direct selection for protein content, rank-based index, and Smith-Hazel index. Gains were calculated based on GEBVs derived from the optimal genomic prediction model.

Five selection strategies were evaluated to identify genotypes capable of simultaneously improving seed yield, oil content, and protein content. The results indicate that achieving positive genetic gains for all three traits at the same time was not feasible under any of the evaluated strategies (Fig 5), reflecting the well-known trade-offs among these traits. Among the tested approaches, the Rank Index produced the most balanced outcome, yielding positive selection gains in seed yield while maintaining oil content at comparable or slightly higher levels. However, consistent with the negative genetic correlation between oil and protein, this strategy reduced protein content. Nevertheless, the magnitude of this reduction was smaller than that observed under the other selection strategies, except when protein content itself was used as the direct selection criterion.

The weights used in the Smith–Hazel index were defined using the genetic standard deviation of each trait. This approach provides a practical method to balance traits measured on different scales while accounting for their genetic variability. However, it is important to recognize that these weights represent a biologically reasonable but not unique solution. In practical breeding programs, index weights are often determined by economic values, breeding objectives, or market demands, and alternative weighting schemes can yield different selection outcomes. For example, prioritizing protein content more strongly or assigning explicit economic values to traits could shift the relative importance of yield, oil, and protein in the selection index. Therefore, the results presented here should be interpreted as one plausible selection scenario illustrating how genomic predictions can be integrated into multi-trait breeding decisions.

## Discussion

### Optimal number of genotypes for training genomic selection models

The determination of the minimum number of genotypes (MNG) required to achieve the highest predictive ability for each location using plateau regression revealed several important insights. Overall, the results demonstrate the need for location-specific optimization in soybean GS. Across most locations, using 60% to 80% of the genotypes in the training population was sufficient to identify the MNG needed to maximize predictive ability across different training population structures and GS methods (Fig 1). However, substantial variability in MNG was observed among locations and models. For instance, Jerico, Lucas, and Urbana required at least 80% of the individuals in the training population to reach peak accuracy in nearly all scenarios, emphasizing the influence of environmental and genetic context on model performance. In contrast, locations such as Ames01 and Lucas displayed relatively low MNG values, indicating that fewer genotypes were needed to achieve high predictive accuracy compared with other environments. This pattern may reflect a higher inherent predictive power at these sites, enabling accurate modeling with smaller training populations [56].

The completely random strategy for most locations generally required a lower MNG than maturity group randomization and the genetic algorithm. This finding suggests that simpler population structures can be effective in certain contexts, potentially reducing the complexity and cost of breeding programs. However, it is also essential to consider genetic diversity and specific traits of interest when selecting the most appropriate strategy [57–59]. Similarly, the results suggest that reducing the number of genotypes evaluated for each location is possible without compromising prediction accuracy. This reduction is significant as it allows for reallocating resources towards expanding the number of locations or increasing the evaluation of critical traits such as disease resistance, iron deficiency, and lodging resistance. By optimizing the number of genotypes, breeding programs can enhance their efficiency and effectiveness, ultimately accelerating the development of improved soybean varieties. Again, the caveat is that it may lead to a genetic bottleneck or reduction in useful genetic variation, leading to a negative outcome for genetic gain in the medium to long term [56,57]. Efficiently designed training populations improve predictive ability and ensure that breeding programs are cost-effective and sustainable in the long term [21,22].

### Comparative analysis of training population structure

The accuracy of GS models tends to decrease as the training and test populations diverge [32], which is why it is crucial to identify the most effective strategies for each trait to enhance selection efficiency. The comparative analysis of training population structure and GS methods provided critical insights into optimizing soybean breeding programs. The experimental random selection (ERS) was the best training population structure across all locations and GS methods.

Results from this work helped develop guidelines and establish a benchmark of a wide range of training set optimization methods under different population structures, genetic architectures, and heritability values, in the context of a public breeding program. An ideal training set optimization method should (i) be able to build a training set that maximizes the accuracy of the GS model by maximizing the relationship between the training set and the test set and by minimizing the relationship within the training set to capture as much genetic variance as possible. [60,61], (ii) reduce the computational burden, and iii) be easy to implement [56,62–64]. We report that the training set has a very significant impact on increasing the accuracy of GS models relative to random sampling [20]. These findings are remarkable given that the experiment random selection (ERS), which involves straightforward selection without specific rules, proved effective. This contrasts with the stratified method, which requires proportional representation by maturity group, and the genetic algorithm, which is more time-consuming and computationally intensive. Despite the simplicity of the ERS, it performed comparably to the more complex methods.

With the advent of GS as a widespread breeding tool, mechanisms to efficiently design an optimal training set for GS models became more relevant, since they allow maximizing the accuracy while minimizing the phenotyping costs [20]. It has been demonstrated that selecting an optimized training set is a crucial factor for accurate genomic predictions [33,65–67]. The optimization aims to maximize the accuracy of the predictions made on a test set while minimizing the size of the training set [68], which reduces phenotyping costs or uses these resources to evaluate other essential traits that cannot be evaluated right now, such as diseases, drought, heat, and flooding.

With the implementation of genomic-assisted breeding tools into breeding programs, the evaluation of germplasm has undergone significant changes. Breeders do not need to evaluate the entire progeny in the field, but rather to optimally select a training set size that will improve the resource allocation within the breeding program. For instance, a reduction in training set size due to optimization implies that more alleles could be tested in the field or that more resources could be invested in more precise phenotyping. Consequently, it is important to optimize the training set size to minimize it without incurring a large loss of accuracy. However, the training set size optimization is computationally intensive as it relies on performing optimization repeatedly for several training set sizes and fitting a function that can predict the evolution of the evaluation criteria as the training set size varies [20].

From a practical breeding perspective, optimized training set strategies, particularly ERS, can be effectively integrated at multiple stages of the soybean breeding pipeline. The greatest impact is expected during early-generation selection, such as the F₂ stage, where single plants are selected and advanced through pod bulking. At this stage, GS could be used to prioritize which plants or pods are advanced to the winter nursery, thereby reducing population size while maintaining genetic diversity and maximizing selection accuracy. Additionally, the method is highly applicable at later stages, such as the F₄ generation, when individual plants are selected for advancement into progeny rows. In this context, GS combined with an optimized training set can improve the identification of superior lines before extensive field evaluation, allowing breeders to allocate resources more efficiently. By reducing the number of lines carried forward without compromising predictive performance, these strategies enable more effective phenotyping, increase selection intensity, and accelerate genetic gain. Therefore, integrating ERS-based training population optimization across both early- and mid-generation stages offers a practical, scalable approach to enhance decision-making in public soybean breeding programs.

Across traits and environments, the GS methods showed comparable predictive performance, with only small differences among approaches. Although SVM and RF occasionally achieved slightly higher predictive abilities, the magnitude of these differences was generally limited. In this context, rrBLUP emerged as a practical and robust GS method, providing stable predictive performance across scenarios while maintaining high computational efficiency and interpretability. Regarding the training population structure, the ERS consistently produced the highest predictive abilities, indicating that this strategy is the most effective for optimizing training populations in this study. Implementing this approach across all locations promises enhanced selection accuracy within the soybean breeding program while optimizing time efficiency. The performances of the different models were also tested by Fernandez-Gonzales et al. [20], and they found them very similar across datasets and different training sets and they recommended simple GS models, such as rrBLUP and GBLUP, as these perform similarly compared to complex models, such as Bayesian models and some machine learning models.

Overall, our study underscores the importance of location-specific optimization in GS for soybean breeding [69,70]. The identification of the minimum number of genotypes required to achieve high predictive ability provides a practical framework for improving the efficiency of breeding programs by guiding the design of training populations and multi-environment trials. In addition, our power analysis highlights the role of environmental diversity in achieving reliable genomic predictions. Although increasing the number of testing locations can substantially improve predictive ability by capturing a broader range of environmental conditions, breeding programs must balance these benefits with logistical and financial constraints. In practice, this may involve optimizing the trade-off between spatial replication (number of locations) and temporal replication (number of years), as well as leveraging historical multi-environment trial data to effectively expand environmental representation. Future research should continue refining these strategies by exploring approaches that increase genotypic diversity and sample size in training populations, particularly in locations where predictive performance remains limited.

### Selection index implementation

In this study, five different selection strategies were evaluated to select genotypes that simultaneously improve seed yield, oil content, and protein content. The results demonstrated that achieving positive selection gains across all three traits simultaneously is not feasible, regardless of the strategy employed, suggesting that trade-offs between traits are inherent when selecting for multiple complex traits [1,71]. While some strategies performed better for specific traits, no single method allowed for the simultaneous improvement of seed yield, oil content, and protein content, highlighting the difficulty in achieving balanced genetic gains across multiple objectives. Among the five strategies tested, the Rank index proved to be the most promising approach, yielding positive genetic gains in both seed yield and oil content. While it resulted in a negative response to selection for protein content, the magnitude of the reduction was lower than other methods, indicating its usefulness. In the soybean breeding programs, several contrasting breeding objectives may be targets, such as traits that have a negative correlation (e.g., seed yield and protein content) or are linked in repulsion. Breeders may be targeting these three most important traits, and each trait will be under polygenic control, leading to more complexity in assembling the desirable combination and an ability to identify that combination [1]. In addition, we can utilize GS to achieve an effective selection outcome for more complex traits, as demonstrated in this paper. DePauw et al. [71] provided a detailed description of negative correlations and breeding strategies in wheat, essentially establishing that traits with negative correlations must be selected simultaneously. While the progress is slower, over the longer term, even seed yield and protein content correlations were positively shifted. The Rank index outperformed the direct selection strategies for each individual trait, suggesting that multi-trait selection approaches may be more effective when aiming for overall improvement. Moreover, such indices can be readily expanded to include additional traits of increasing relevance to soybean breeding programs, such as heat tolerance, which has become a critical target in the U.S. Midwest due to rising temperatures and increased climate variability [72].

A crucial consideration in implementing GS is the necessity to maintain genetic diversity in the selection of parents and breeding populations [73,74]. While selecting specific traits can drive genetic improvement, preserving the variability of the gene pool is crucial to avoid narrowing the genetic diversity. Genetic diversity can be measured using phenotypic or molecular tools; however, it is crucial to consider the diversity of the genetic basis of multiple traits. For example, seed yield, protein content, oil content, abiotic and biotic tolerance, quality traits, and more [75–77]. Genetic diversity enables more adaptive responses to changing environmental conditions and reduces the risk of selecting undesirable alleles that could negatively impact broader breeding objectives [78]. The breeding program should consider incorporating diversity management strategies to overcome these challenges.

In this context, phenomic selection emerges as a valuable complementary approach to GS. High-throughput phenotyping enables the collection of detailed, multi-trait phenotypic data across environments and developmental stages, capturing complex plant responses that may not be fully explained by genomic data alone [79]. By integrating phenomic information with GS models, breeders can improve prediction accuracy, better account for genotype × environment interactions, and enhance the selection of superior genotypes while maintaining genetic diversity. Moreover, phenomic selection can support the identification of complementary parental combinations, thereby contributing to increased genetic gain without excessively narrowing the genetic base of breeding populations.

From an applied breeding perspective, this study's results provide several practical guidelines for implementing GS in public soybean breeding programs. Our findings indicate that rrBLUP represents an effective and robust baseline model for genomic prediction across traits. To maximize predictive performance, the training population should include approximately 80% of the available genotypes, which ensures stable parameter estimation and reliable predictions. In addition, assembling the training population through experimental random selection helps maintain genetic diversity and representativeness, which are critical for sustaining predictive ability across breeding cycles. The results also highlight the importance of conducting evaluations across an adequate number of testing environments, with the required number of locations varying according to the trait and the degree of population stratification. Finally, when the objective is the simultaneous improvement of multiple traits, a rank-based selection index provides an efficient strategy for achieving balanced genetic progress, particularly for improving seed yield and oil content while accepting a moderate reduction in protein content. Together, these recommendations offer a practical framework for optimizing GS strategies in public soybean breeding programs.

## Conclusion

The most effective strategy for implementing genomic selection in a public soybean breeding program is to use the rrBLUP model. To maximize predictive performance, the model should be trained with approximately 80% of the total population, ensuring robust and reliable estimates. Incorporating experimental random selection to assemble the training population further increases genetic diversity, which is essential for improving predictive ability and maintaining consistent performance across breeding cycles. Together, these practices provide a strong foundation for maximizing genetic gains within the program.

The Rank Index is also a highly effective strategy for selecting soybean genotypes, especially when the goal is to improve multiple traits simultaneously. By integrating information from several traits, this method enables more balanced and comprehensive genetic progress than single-trait selection approaches, making it a valuable tool for breeding programs aiming to enhance overall productivity and quality.

## Supporting information

S1 Table Information about the 16 locations used to perform the genomic selection methods. (DOCX)

S2 Table Percentage of outliers removed per trait across environments and years. (DOCX)

S3 Table Regression parameters from plateau models were used to determine the minimum number of genotypes (MNG) required to reach the highest predictive ability at each location. (DOCX)

S1 Fig Prediction accuracy of all genomic selection models across all locations under the Random selection training-population structure strategy, using 80% of the individuals for model training. Genomic selection models followed by different letters differ significantly according to Tukey’s test at the 5% significance level.
(TIFF)

S2 Fig Prediction accuracy of all genomic selection models across all locations under the Maturity group random selection training-population structure strategy, using 80% of the individuals for model training. Genomic selection models followed by different letters differ significantly according to Tukey’s test at the 5% significance level.
(TIFF)

S3 Fig Prediction accuracy of all genomic selection models across all locations under the Experiment random selection training-population structure strategy, using 80% of the individuals for model training. Genomic selection models followed by different letters differ significantly according to Tukey’s test at the 5% significance level.
(TIFF)

S4 Fig Prediction accuracy of all genomic selection models across all locations under the Genetic Algorithm training-population structure strategy, using 80% of the individuals for model training. Genomic selection models followed by different letters differ significantly according to Tukey’s test at the 5% significance level.
(TIFF)

S5 Fig Prediction accuracy across all locations for seed yield, protein (Pro), oil, and maturity (Mat) under four training-population structures: Random (random selection), MG (maturity-group random selection), Exp (experimental random selection), and GA (genetic algorithm), using the Bayes A genomic selection model. Locations followed by different letters differ significantly according to Tukey’s test at the 5% significance level.
(TIFF)

S6 Fig Prediction accuracy across all locations for seed yield, protein (Pro), oil, and maturity (Mat) under four training-population structures: Random (random selection), MG (maturity-group random selection), Exp (experimental random selection), and GA (genetic algorithm), using the Bayes B genomic selection model. Locations followed by different letters differ significantly according to Tukey’s test at the 5% significance level.
(TIFF)

S7 Fig Prediction accuracy across all locations for seed yield, protein (Pro), oil, and maturity (Mat) under four training-population structures: Random (random selection), MG (maturity-group random selection), Exp (experimental random selection), and GA (genetic algorithm), using the RKHS genomic selection model. Locations followed by different letters differ significantly according to Tukey’s test at the 5% significance level.
(TIFF)

S8 Fig Prediction accuracy across all locations for seed yield, protein (Pro), oil, and maturity (Mat) under four training-population structures: Random (random selection), MG (maturity-group random selection), Exp (experimental random selection), and GA (genetic algorithm), using the Random forest genomic selection model. Locations followed by different letters differ significantly according to Tukey’s test at the 5% significance level.
(TIFF)

S9 Fig Prediction accuracy across all locations for seed yield, protein (Pro), oil, and maturity (Mat) under four training-population structures: Random (random selection), MG (maturity-group random selection), Exp (experimental random selection), and GA (genetic algorithm), using the support vector machine genomic selection model. Locations followed by different letters differ significantly according to Tukey’s test at the 5% significance level.
(TIFF)

S10 Fig Prediction accuracy across all training population structures for seed yield, protein (Pro), oil, and maturity (Mat) under eight locations: Ames 01, Ames 02, Crawfordsville, Jerico, Lucas, Rockport, Sutherland, and Urbana, using the Bayes A genomic selection model. Training population structures followed by different letters differ significantly according to Tukey’s test at the 5% significance level.
(TIFF)

S11 Fig Prediction accuracy across all training population structures for seed yield, protein (Pro), oil, and maturity (Mat) under eight locations: Ames 01, Ames 02, Crawfordsville, Jerico, Lucas, Rockport, Sutherland, and Urbana, using the Bayes B genomic selection model. Training population structures followed by different letters differ significantly according to Tukey’s test at the 5% significance level.
(TIFF)

S12 Fig Prediction accuracy across all training population structures for seed yield, protein (Pro), oil, and maturity (Mat) under eight locations: Ames 01, Ames 02, Crawfordsville, Jerico, Lucas, Rockport, Sutherland, and Urbana, using the RKHS genomic selection model. Training population structures followed by different letters differ significantly according to Tukey’s test at the 5% significance level.
(TIFF)

S13 Fig Prediction accuracy across all training population structures for seed yield, protein (Pro), oil, and maturity (Mat) under eight locations: Ames 01, Ames 02, Crawfordsville, Jerico, Lucas, Rockport, Sutherland, and Urbana, using the Random Forest genomic selection model. Training population structures followed by different letters differ significantly according to Tukey’s test at the 5% significance level.
(TIFF)

S14 Fig Prediction accuracy across all training population structures for seed yield, protein (Pro), oil, and maturity (Mat) under eight locations: Ames 01, Ames 02, Crawfordsville, Jerico, Lucas, Rockport, Sutherland, and Urbana, using the Support Vector Machine genomic selection model. Training population structures followed by different letters differ significantly according to Tukey’s test at the 5% significance level.
(TIFF)
