# Supplementary material for: Strategies for implementing genomic selection in a public soybean breeding program
Source: PLoS One. 2026 Jul 13;21(7):e0353481. doi: 10.1371/journal.pone.0353481 (PMC13362134; doi:10.1371/journal.pone.0353481)
Supplement: S1 Table — (DOCX) [file pone.0353481.s001.docx]

| **Year** | **Location** | **Number of replications** | **Number of Experimental lines** | **Harvest Date** | **Plot Length** | **Alley Length** | **Number of Rows** | **Row Spacing** | **Planter** | **Fertilizer** | **Chemicals** | **Harvester** |
| --- | --- | --- | --- | --- | --- | --- | --- | --- | --- | --- | --- | --- |
| 2023 | Ames 30 | 2 | 358 | 10/31/2023 | 15' | 3' | 2 | 30" | Amaco 4 row cone | Conventional tillage | Conventional Herbicides | Zurn 150 |
| 2023 | Ames 15 | 2 | 358 | 10/29/2023 | 15' | 3' | 3 | 15" | SRES Drill | Conventional tillage | Conventional Herbicides | Zurn 150 |
| 2023 | Sutherland | 2 | 196 | 9/10/2023 | 15' | 3' | 2 | 30" | Amaco 4 row cone | Conventional tillage | Conventional Herbicides | Zurn 150 |
| 2023 | Floyd | 2 | 198 | 10/5/2023 | 15' | 3' | 2 | 30" | Amaco 4 row cone | Conventional tillage | Conventional Herbicides | Zurn 150 |
| 2023 | Crawfordsville | 2 | 358 | 10/7/2023 | 15' | 3' | 2 | 30" | Amaco 4 row cone | Conventional tillage | Conventional Herbicides | Zurn 150 |
| 2023 | McNay | 2 | 312 | 10/3/2023 | 15' | 3' | 2 | 30" | Amaco 4 row cone | Conventional tillage | Conventional Herbicides | Zurn 150 |
| 2023 | University of Illinois | 2 | 164 | 9/27/2023 | 12' | 3' | 2 | 30" | NA | NA | Conventional Herbicides | NA |
| 2023 | University of Missouri | 2 | 165 | 10/3/2023 | 15' | 3' | 2 | 30" | NA | NA | Conventional Herbicides | NA |
| 2024 | Ames 30 | 2 | 462 | 10/9/2024 | 12' | 3' | 2 | 30" | Amaco 4 row cone | Conventional tillage | Conventional Herbicides | Zurn 150 |
| 2024 | Ames 15 | 2 | 462 | 10/11/2024 | 12' | 3' | 3 | 15" | SRES Drill | Conventional tillage | Conventional Herbicides | Zurn 150 |
| 2024 | Sutherland | 2 | 237 | 10/21/2024 | 12' | 3' | 2 | 30" | Amaco 4 row cone | Conventional tillage | Conventional Herbicides | Zurn 150 |
| 2024 | Kanawha | 2 | 116 | 9/30/2024 | 12' | 3' | 2 | 30" | Amaco 4 row cone | Conventional tillage | Conventional Herbicides | Zurn 150 |
| 2024 | Nashua | 2 | 116 | 9/27/2024 | 12' | 3' | 2 | 30" | Amaco 4 row cone | Conventional tillage | Conventional Herbicides | Zurn 150 |
| 2024 | McNay | 2 | 350 | 10/5/2024 | 12' | 3' | 2 | 30" | Amaco 4 row cone | Conventional tillage | Conventional Herbicides | Zurn 150 |
| 2024 | University of Illinois | 1 | 350 | 10/8/2024 | 12' | 3' | 2 | 30" | NA | NA | Conventional Herbicides | NA |
| 2024 | University of Missouri | 2 | 165 | 10/3/2024 | 15' | 3' | 2 | 30" | NA | NA | Conventional Herbicides | NA |
