# Supplementary material for: Strategies for implementing genomic selection in a public soybean breeding program
Source: PLoS One. 2026 Jul 13;21(7):e0353481. doi: 10.1371/journal.pone.0353481 (PMC13362134; doi:10.1371/journal.pone.0353481)
Supplement: S2 Table — (DOCX) [file pone.0353481.s002.docx]

| **Year** | **Location** | **Number of Outliers Seed Yield** | **Number of Outliers Oil Content** | **Number of Outliers Protein Content** | **Number of Outliers Maturity** |
| --- | --- | --- | --- | --- | --- |
| 2023 | Ames 30 | 2.01% | 0.73% | 0.27% | - |
| 2023 | Ames 15 | 0.60% | - | - | - |
| 2023 | Sutherland | 1.44% | - | - | 0.83% |
| 2023 | Floyd | - | - | - | - |
| 2023 | Crawfordsville | 1.01% | 1.26% | 1.01% | - |
| 2023 | McNay | 1.47% | - | 0.99% | - |
| 2023 | University of Illinois | 4.10% | 0.99% | 1.50% | - |
| 2023 | University of Missouri | - | 0.64% | 0.32% | 2.99% |
| 2024 | Ames 30 | 1.95% | 0.67% | 0.54% | 1.94% |
| 2024 | Ames 15 | 0.70% | - | - | - |
| 2024 | Sutherland | 1.12% | - | - | - |
| 2024 | Kanawha | - | 0.15% | 0.55% | - |
| 2024 | Nashua | 1.20% | - | - | - |
| 2024 | McNay | 1.36% | - | - | - |
| 2024 | University of Illinois | 3.55% | 1.35% | 1.43% | - |
| 2024 | University of Missouri | 0.15% | 0.76% | 0.45% | 0.54% |
