# Supplementary material for: Strategies for implementing genomic selection in a public soybean breeding program
Source: PLoS One. 2026 Jul 13;21(7):e0353481. doi: 10.1371/journal.pone.0353481 (PMC13362134; doi:10.1371/journal.pone.0353481)
Supplement: S3 Table — (DOCX) [file pone.0353481.s003.docx]

| **Trait** | **TRStructure** | **Model** | **Breakpoint** | **R2** | **Accuracy_Breakpoint** |
| --- | --- | --- | --- | --- | --- |
| Seeds yield | Exp | rrblup | 58 | 0.93 | 0.72 |
| Seeds yield | Exp | BayesA | 58 | 0.93 | 0.73 |
| Seeds yield | Exp | BayesB | 58 | 0.92 | 0.73 |
| Seeds yield | Exp | RKHS | 59 | 0.93 | 0.73 |
| Seeds yield | Exp | RF | 68 | 0.94 | 0.88 |
| Seeds yield | Exp | SVM | 79 | 0.95 | 0.88 |
| Seeds yield | GA | rrblup | 79 | 0.78 | 0.50 |
| Seeds yield | GA | BayesA | 79 | 0.80 | 0.51 |
| Seeds yield | GA | BayesB | 79 | 0.80 | 0.51 |
| Seeds yield | GA | RKHS | 78 | 0.79 | 0.51 |
| Seeds yield | GA | RF | 84 | 0.83 | 0.55 |
| Seeds yield | GA | SVM | 68 | 0.90 | 0.41 |
| Seeds yield | MG | rrblup | 68 | 0.92 | 0.74 |
| Seeds yield | MG | BayesA | 59 | 0.91 | 0.73 |
| Seeds yield | MG | BayesB | 58 | 0.91 | 0.73 |
| Seeds yield | MG | RKHS | 59 | 0.90 | 0.73 |
| Seeds yield | MG | RF | 68 | 0.94 | 0.88 |
| Seeds yield | MG | SVM | 79 | 0.94 | 0.89 |
| Seeds yield | Random | rrblup | 60 | 0.89 | 0.72 |
| Seeds yield | Random | BayesA | 59 | 0.90 | 0.73 |
| Seeds yield | Random | BayesB | 58 | 0.90 | 0.72 |
| Seeds yield | Random | RKHS | 60 | 0.90 | 0.73 |
| Seeds yield | Random | RF | 69 | 0.94 | 0.88 |
| Seeds yield | Random | SVM | 79 | 0.94 | 0.89 |
| Oil content | Exp | rrblup | 58 | 0.91 | 0.82 |
| Oil content | Exp | BayesA | 59 | 0.91 | 0.82 |
| Oil content | Exp | BayesB | 59 | 0.91 | 0.82 |
| Oil content | Exp | RKHS | 58 | 0.91 | 0.82 |
| Oil content | Exp | RF | 68 | 0.93 | 0.90 |
| Oil content | Exp | SVM | 80 | 0.95 | 0.86 |
| Oil content | GA | rrblup | 58 | 0.81 | 0.62 |
| Oil content | GA | BayesA | 57 | 0.83 | 0.62 |
| Oil content | GA | BayesB | 57 | 0.83 | 0.62 |
| Oil content | GA | RKHS | 60 | 0.81 | 0.62 |
| Oil content | GA | RF | 56 | 0.85 | 0.63 |
| Oil content | GA | SVM | 88 | 0.85 | 0.45 |
| Oil content | MG | rrblup | 58 | 0.92 | 0.82 |
| Oil content | MG | BayesA | 58 | 0.91 | 0.82 |
| Oil content | MG | BayesB | 58 | 0.91 | 0.82 |
| Oil content | MG | RKHS | 58 | 0.91 | 0.82 |
| Oil content | MG | RF | 60 | 0.92 | 0.88 |
| Oil content | MG | SVM | 87 | 0.96 | 0.90 |
| Oil content | Random | rrblup | 60 | 0.93 | 0.81 |
| Oil content | Random | BayesA | 67 | 0.95 | 0.83 |
| Oil content | Random | BayesB | 66 | 0.95 | 0.83 |
| Oil content | Random | RKHS | 59 | 0.93 | 0.81 |
| Oil content | Random | RF | 69 | 0.94 | 0.90 |
| Oil content | Random | SVM | 80 | 0.95 | 0.86 |
| Protein content | Exp | rrblup | 70 | 0.93 | 0.88 |
| Protein content | Exp | BayesA | 76 | 0.95 | 0.89 |
| Protein content | Exp | BayesB | 76 | 0.95 | 0.89 |
| Protein content | Exp | RKHS | 70 | 0.93 | 0.88 |
| Protein content | Exp | RF | 77 | 0.93 | 0.93 |
| Protein content | Exp | SVM | 87 | 0.96 | 0.92 |
| Protein content | GA | rrblup | 55 | 0.73 | 0.72 |
| Protein content | GA | BayesA | 52 | 0.75 | 0.72 |
| Protein content | GA | BayesB | 52 | 0.74 | 0.72 |
| Protein content | GA | RKHS | 53 | 0.76 | 0.72 |
| Protein content | GA | RF | 54 | 0.76 | 0.71 |
| Protein content | GA | SVM | 70 | 0.64 | 0.42 |
| Protein content | MG | rrblup | 70 | 0.94 | 0.88 |
| Protein content | MG | BayesA | 70 | 0.93 | 0.88 |
| Protein content | MG | BayesB | 70 | 0.93 | 0.88 |
| Protein content | MG | RKHS | 69 | 0.93 | 0.88 |
| Protein content | MG | RF | 70 | 0.93 | 0.92 |
| Protein content | MG | SVM | 80 | 0.94 | 0.88 |
| Protein content | Random | rrblup | 69 | 0.92 | 0.88 |
| Protein content | Random | BayesA | 70 | 0.92 | 0.88 |
| Protein content | Random | BayesB | 69 | 0.92 | 0.88 |
| Protein content | Random | RKHS | 60 | 0.88 | 0.87 |
| Protein content | Random | RF | 70 | 0.93 | 0.92 |
| Protein content | Random | SVM | 77 | 0.94 | 0.87 |
| Maturity | Exp | rrblup | 57 | 0.87 | 0.88 |
| Maturity | Exp | BayesA | 53 | 0.85 | 0.86 |
| Maturity | Exp | BayesB | 53 | 0.84 | 0.86 |
| Maturity | Exp | RKHS | 56 | 0.87 | 0.87 |
| Maturity | Exp | RF | 56 | 0.86 | 0.90 |
| Maturity | Exp | SVM | 60 | 0.91 | 0.87 |
| Maturity | GA | rrblup | 57 | 0.80 | 0.66 |
| Maturity | GA | BayesA | 58 | 0.81 | 0.67 |
| Maturity | GA | BayesB | 58 | 0.82 | 0.69 |
| Maturity | GA | RKHS | 57 | 0.81 | 0.66 |
| Maturity | GA | RF | 58 | 0.82 | 0.70 |
| Maturity | GA | SVM | 59 | 0.88 | 0.58 |
| Maturity | MG | rrblup | 59 | 0.93 | 0.88 |
| Maturity | MG | BayesA | 55 | 0.91 | 0.87 |
| Maturity | MG | BayesB | 55 | 0.90 | 0.86 |
| Maturity | MG | RKHS | 59 | 0.92 | 0.87 |
| Maturity | MG | RF | 57 | 0.88 | 0.90 |
| Maturity | MG | SVM | 59 | 0.94 | 0.86 |
| Maturity | Random | rrblup | 58 | 0.88 | 0.87 |
| Maturity | Random | BayesA | 53 | 0.86 | 0.86 |
| Maturity | Random | BayesB | 53 | 0.84 | 0.86 |
| Maturity | Random | RKHS | 58 | 0.88 | 0.87 |
| Maturity | Random | RF | 55 | 0.84 | 0.90 |
| Maturity | Random | SVM | 68 | 0.94 | 0.88 |
