# Supplementary material for: Strategies for implementing genomic selection in a public soybean breeding program
Source: PLoS One. 2026 Jul 13;21(7):e0353481. doi: 10.1371/journal.pone.0353481 (PMC13362134; doi:10.1371/journal.pone.0353481)
Supplement: Attachment — Submitted filename: Reviewer comment-GC.pdf [file pone.0353481.s019.pdf]

Strategies for Implementing Genomic Selection in a Public Soybean Breeding Program

Generally, the study is comprehensive and well-structured and relevant topic in modern plant breeding landscape. The study presents a systematic analysis of relevant parameters both in breadth and depth and the applications of such analysis in practicable plant breeding programs. The manuscript can be accepted with some revisions, especially clarifying the model used because **Gi in line 151** should be random. The author needs to correct Model equation formatting errors, minor typographical errors.
