# Supplementary material for: Strategies for implementing genomic selection in a public soybean breeding program
Source: PLoS One. 2026 Jul 13;21(7):e0353481. doi: 10.1371/journal.pone.0353481 (PMC13362134; doi:10.1371/journal.pone.0353481)
Supplement: Attachment — Submitted filename: PONE-D-26-02573_review.pdf [file pone.0353481.s020.pdf]

PLOS One

# Strategies for Implementing Genomic Selection in a Public Soybean Breeding Program

--Manuscript Draft--

| | |
|---|---|
| Manuscript Number: | PONE-D-26-02573 |
| Article Type: | Research Article |
| Full Title: | Strategies for Implementing Genomic Selection in a Public Soybean Breeding Program |
| Short Title: | Genomic Selection Strategies in Soybean Breeding |
| Corresponding Author: | Leonardo de Azevedo Peixoto, PhD<br>Iowa State University College of Agriculture and Life Sciences<br>Ames, UNITED STATES OF AMERICA |
| Keywords: | Glycine max; genomic prediction; training population structure; Machine Learning; Bayesian models |
| Abstract: | Improving selection accuracy in soybean breeding programs is crucial for reducing costs and shortening the time required to develop new varieties. Genomic selection (GS) is a promising tool for enhancing the accuracy of selection. This study aimed to identify the most effective GS models for predicting key traits, including yield, protein content, oil content, and maturity in soybean breeding programs. Additionally, we sought to determine the optimal training population optimization method (structure vs. size) and establish the minimum number of genotypes required for a training population that ensures high model performance across locations. Finally, we explored multitrait selection based on genomic prediction to improve breeding decisions. Data were obtained from the soybean variety development program and included experiments planted in a randomized block design with two replications at eight locations during the 2023 and 2024 growing seasons. Six genomic selection models were tested: rrBLUP, Bayes A, Bayes B, RKHS, random forest, and support vector machine. Four methods for optimizing the training population were evaluated: random selection (RS), maturity group random selection (MGRS), experimental random selection (ERS), and genetic algorithm (GA). We also assessed ten different training population sizes (ranging from 10% to 90%) and five selection index strategies: direct selection on yield, direct selection on oil content, direct selection on protein content, Rank-index, and Smith-Hazel index. Our results suggest that the most effective strategy for implementing genomic selection in a public soybean breeding program involves using the rrBLUP model for genomic prediction. To optimize its performance, it is recommended to train the model using 80% of the total population. This approach provides a robust and reliable prediction. Furthermore, structuring the training population through experimental random selection enhances genetic diversity, which is crucial for improving selection accuracy and robustness across breeding cycles. Finally, the Rank-index proved to be a highly effective strategy for selecting soybean genotypes, particularly for improving seed yield and oil content, while results for seed protein were less promising. By considering multiple traits simultaneously, this method offers a more balanced approach to genetic improvement compared to single-trait selection methods, making it an excellent tool for breeding programs that aim to enhance both productivity and quality. |
| Order of Authors: | Leonardo de Azevedo Peixoto, PhD |
| | Eliana Monteverde Dominguez |
| | Andrew Scaboo |
| | Reka Howard |
| | Asheesh Singh |
| Additional Information: | |
| Question | Response |
| Financial Disclosure | Yes |

Powered by Editorial Manager® and ProduXion Manager® from Aries Systems Corporation

| | |
|---|---|
| Enter a financial disclosure statement that describes the sources of funding for the work included in this submission. Review the submission guidelines for detailed requirements. View published research articles from *PLOS ONE* for specific examples.<br><br>This statement is required for submission and **will appear in the published article** if the submission is accepted. Please make sure it is accurate.<br><br>**Funded studies**<br>Enter a statement with the following details:<br>• Initials of the authors who received each award<br>• Grant numbers awarded to each author<br>• The full name of each funder<br>• URL of each funder website<br>• Did the sponsors or funders play any role in the study design, data collection and analysis, decision to publish, or preparation of the manuscript?<br><br>Did you receive funding for this work? | |
| Please add funding details.<br>as follow-up to "**Financial Disclosure**<br><br>Enter a financial disclosure statement that describes the sources of funding for the work included in this submission. Review the submission guidelines for detailed requirements. View published research articles from *PLOS ONE* for specific examples.<br><br>This statement is required for submission and **will appear in the published article** if the submission is accepted. Please make sure it is accurate. | Iowa Soybean Association<br>R.F. Baker Center for Plant Breeding<br>Plant Sciences Institute<br>North Central Soybean Research Program<br>USDA CRIS project IOW04714<br>AI Institute for Resilient Agriculture (USDA-NIFA 2021-67021-35329)<br>G.F. Sprague Chair in Agronomy |

| | |
|---|---|
| **Funded studies**<br>Enter a statement with the following details:<br>• Initials of the authors who received each award<br>• Grant numbers awarded to each author<br>• The full name of each funder<br>• URL of each funder website<br>• Did the sponsors or funders play any role in the study design, data collection and analysis, decision to publish, or preparation of the manuscript?<br><br>Did you receive funding for this work?" | |
| Please select the country of your main research funder (please select carefully as in some cases this is used in fee calculation).<br>as follow-up to "**Financial Disclosure**<br><br>Enter a financial disclosure statement that describes the sources of funding for the work included in this submission. Review the submission guidelines for detailed requirements. View published research articles from *PLOS ONE* for specific examples.<br><br>This statement is required for submission and **will appear in the published article** if the submission is accepted. Please make sure it is accurate.<br><br>**Funded studies**<br>Enter a statement with the following details:<br>• Initials of the authors who received each award<br>• Grant numbers awarded to each author<br>• The full name of each funder<br>• URL of each funder website<br>• Did the sponsors or funders play any role in the study design, data collection and analysis, decision to publish, or preparation of the manuscript?<br><br>Did you receive funding for this work?" | UNITED STATES - US |
| **Competing Interests** | NO authors have competing interests |

| | |
|---|---|
| Use the instructions below to enter a competing interest statement for this submission. On behalf of all authors, disclose any competing interests that could be perceived to bias this work—acknowledging all financial support and any other relevant financial or non-financial competing interests.<br><br>This statement is required for submission and **will appear in the published article** if the submission is accepted. Please make sure it is accurate and that any funding sources listed in your Funding Information later in the submission form are also declared in your Financial Disclosure statement.<br><br>View published research articles from *PLOS ONE* for specific examples.<br><br>**NO authors have competing interests**<br><br>Enter: *The authors have declared that no competing interests exist.*<br><br>**Authors with competing interests**<br><br>Enter competing interest details beginning with this statement:<br><br>*I have read the journal's policy and the authors of this manuscript have the following competing interests: [insert competing interests here]*<br><br>* typeset | |
| **Ethics Statement**<br><br>Enter an ethics statement for this submission. This statement is required if the study involved:<br><br>• Human participants<br>• Human specimens or tissue<br>• Vertebrate animals or cephalopods<br>• Vertebrate embryos or tissues | NA |

| | |
|---|---|
| • Field research<br><br>Write "N/A" if the submission does not require an ethics statement.<br><br>General guidance is provided below. Consult the submission guidelines for detailed instructions. **Make sure that all information entered here is included in the Methods section of the manuscript.** | |

| | |
|---|---|
| **Format for specific study types**<br><br>**Human Subject Research (involving human participants and/or tissue)**<br>• Give the name of the institutional review board or ethics committee that approved the study<br>• Include the approval number and/or a statement indicating approval of this research<br>• Indicate the form of consent obtained (written/oral) or the reason that consent was not obtained (e.g. the data were analyzed anonymously)<br><br>**Animal Research (involving vertebrate animals, embryos or tissues)**<br>• Provide the name of the Institutional Animal Care and Use Committee (IACUC) or other relevant ethics board that reviewed the study protocol, and indicate whether they approved this research or granted a formal waiver of ethical approval<br>• Include an approval number if one was obtained<br>• If the study involved *non-human primates*, add *additional details* about animal welfare and steps taken to ameliorate suffering<br>• If anesthesia, euthanasia, or any kind of animal sacrifice is part of the study, include briefly which substances and/or methods were applied<br><br>**Field Research**<br><br>Include the following details if this study involves the collection of plant, animal, or other materials from a natural setting:<br>• Field permit number<br>• Name of the institution or relevant body that granted permission | |
| **Data Availability**<br><br>Authors are required to make all data underlying the findings described fully available, without restriction, and from the time of publication. PLOS allows rare exceptions to address legal and ethical concerns. See the PLOS Data Policy and FAQ for detailed information. | Yes - all data are fully available without restriction |

| | |
|---|---|
| A Data Availability Statement describing where the data can be found is required at submission. Your answers to this question constitute the Data Availability Statement and **will be published in the article**, if accepted.<br><br>**Important:** Stating 'data available on request from the author' is not sufficient. If your data are only available upon request, select 'No' for the first question and explain your exceptional situation in the text box.<br><br>Do the authors confirm that all data underlying the findings described in their manuscript are fully available without restriction? | |
| **Describe where the data may be found in full sentences. If you are copying our sample text, replace any instances of XXX with the appropriate details.**<br><br>• If the data are **held or will be held in a public repository**, include URLs, accession numbers or DOIs. If this information will only be available after acceptance, indicate this by ticking the box below. For example: *All XXX files are available from the XXX database (accession number(s) XXX, XXX.).*<br>• If the data are all contained **within the manuscript and/or Supporting Information files**, enter the following: *All relevant data are within the manuscript and its Supporting Information files.*<br>• If neither of these applies but you are able to provide **details of access elsewhere**, with or without limitations, please do so. For example:<br><br>*Data cannot be shared publicly because of [XXX]. Data are available from the XXX Institutional Data Access / Ethics Committee (contact via XXX) for researchers who meet the criteria for access to confidential data.*<br><br>*The data underlying the results presented in the study are available from (include the name of the third party* | The data underlying the results presented in the study are available from corresponding authors. |

| | |
|---|---|
| *and contact information or URL).*<br>• This text is appropriate if the data are owned by a third party and authors do not have permission to share the data.<br><br>* typeset | |
| Additional data availability information: | |

Manuscript
Click here to access/download;Manuscript;Plos One manuscript final version.docx

# Strategies for Implementing Genomic Selection in a Public Soybean Breeding Program

Leonardo De Azevedo Peixoto[1*], Eliana Monteverde Dominguez[2], Andrew Scaboo[3], Reka Howard[4], Asheesh K. Singh[1*]

[1] Department of Agronomy, Iowa State University, Ames, Iowa, United States of America

[2] Department of Crop Sciences, University of Illinois, Urbana, Illinois, United States of America

[3] Division of Plant Science and Technology, University of Missouri, Columbia, Missouri, United States of America

[4] Department of Statistics, University of Nebraska–Lincoln, Lincoln, Nebraska, United States of America

* Corresponding author

E-mail: leoazevedop@gmail.com; singhak@iastate.edu

# Abstract

Improving selection accuracy in soybean breeding programs is crucial for reducing costs and shortening the time required to develop new varieties. Genomic selection (GS) is a promising tool for enhancing the accuracy of selection. This study aimed to identify the most effective GS models for predicting key traits, including yield, protein content, oil content, and maturity in soybean breeding programs. Additionally, we sought to determine the optimal training population optimization method (structure vs. size) and establish the minimum number of genotypes required for a training population that ensures high model performance across locations. Finally, we explored multitrait selection based on genomic prediction to improve breeding decisions. Data were obtained from the soybean variety development program and included experiments planted in a randomized block design with two replications at eight locations during the 2023 and 2024 growing seasons. Six genomic selection models were tested: rrBLUP, Bayes A, Bayes B, RKHS, random forest, and support vector machine. Four methods for optimizing the training population were evaluated: random selection (RS), maturity group random selection (MGRS), experimental random selection (ERS), and genetic algorithm (GA). We also assessed ten different training population sizes (ranging from 10% to 90%) and five selection index strategies: direct selection on yield, direct selection on oil content, direct selection on protein content, Rank-index, and Smith-Hazel index. Our results suggest that the most effective strategy for implementing genomic selection in a public soybean breeding program involves using the rrBLUP model for genomic prediction. To optimize its performance, it is recommended to train the model using 80% of the total population. This approach provides a robust and reliable prediction. Furthermore, structuring the training population through experimental random selection enhances genetic diversity, which is crucial for improving selection accuracy and robustness across breeding cycles. Finally, the

Rank-index proved to be a highly effective strategy for selecting soybean genotypes, particularly for improving seed yield and oil content, while results for seed protein were less promising. By considering multiple traits simultaneously, this method offers a more balanced approach to genetic improvement compared to single-trait selection methods, making it an excellent tool for breeding programs that aim to enhance both productivity and quality.

**Keywords:** *Glycine max*, genomic prediction, training population structure, machine learning, Bayesian models

# Introduction

Traditional soybean breeding methods rely heavily on phenotypic selection, which is crucial for informed decision-making [1]. However, there are significant challenges in phenotype only selection methods, particularly for low heritability traits, as these are often limited by environmental variability and lower accuracy of phenotypic data. These methods are time-consuming and resource-intensive, posing significant challenges for breeders aiming to improve complex traits such as yield, protein content, and oil composition [2]. Genomic selection offers a solution by enabling the selection of individuals based on their genetic potential before phenotypic traits are fully expressed [1-3]. This method reduces the duration and cost of field trials, while also enhancing selection accuracy, particularly for polygenic traits [4].

Soybean breeding programs focus on several key traits, including improving yield, enhancing resistance to pests and diseases, and increasing tolerance to abiotic stresses such as drought [5-6]. Genomic selection (GS) has emerged as a transformative approach in plant breeding, offering a powerful method to enhance the efficiency and accuracy of selecting superior crop varieties [7]. Soybean [*Glycine max* (L.) Merr.], a vital oilseed crop with significant economic

and nutritional importance, benefits significantly from integrating GS into its breeding programs [8]. This approach utilizes dense molecular markers distributed across the genome to predict the breeding values of plants, thereby accelerating the breeding cycle and enhancing selection precision [9]. The integration of GS for trait prediction and selection holds significant promise for addressing these challenges by enabling the simultaneous improvement of multiple traits [10]. Moreover, GS facilitates the utilization of a broader genetic base, thereby increasing the potential for genetic gains [11].

Several studies have demonstrated the effectiveness of GS in soybean breeding [12-14]. Notable improvements have been observed in traits such as seed yield [12], protein content [15], and resistance to pathogens [16], supported by advancements in high-throughput genotyping and the development of sophisticated statistical models capable of managing large genomic datasets [17]. Techniques such as ridge regression best linear unbiased prediction (RR-BLUP) [12], Bayesian approaches like Bayes A and Bayes B [18], and kernel-based methods such as reproducing kernel Hilbert spaces (RKHS) [19] have shown significant promise in GS for soybean. However, several factors can influence the prediction accuracy estimated by GS models in the soybean breeding program, such as training population structure [20], training population size [21], number of markers [22], GS methods [22-23], and number of locations [18].

The composition of the training population (TR) is a critical factor influencing the accuracy of genomic predictions. Larger TRs tend to improve predictive ability [24], but simply increasing size is not always optimal. Low genetic diversity in the TR limits the accuracy of estimating genotypic effects [25], while excessive diversity, especially from individuals genetically distant from the target population (TE), can reduce prediction accuracy [26]. Thus, striking the right balance between diversity and relatedness is essential. Various strategies have been developed to

optimize TR composition [27-28] and trait-specific or bin-weighted relationship matrices [29-30]. However, these methods often depend on population structure and trait architecture. In this context, genetic algorithms (GAs) have gained attention for their flexibility and efficiency in optimizing TRs [31]. By mimicking natural selection through iterative processes such as selection, crossover, and mutation, GAs effectively explore complex solution spaces to identify optimal subsets. Studies have shown that GAs can outperform traditional approaches in selecting training sets that maximize prediction accuracy [32-34].

The number and quality of markers used in genomic selection significantly impact prediction accuracy and program efficiency. High-density SNP arrays offer excellent genome coverage but are costly, especially when genotyping large populations. Genotyping-by-sequencing (GBS) is more affordable, although it may introduce high levels of missing data and lower genotype quality [35]. Studies have shown that increasing marker density does not always improve prediction accuracy [36-37]. In some cases, carefully selected subsets of markers outperform full datasets [29]. Marker selection based on genome distribution, QTL association, or effect size has shown promise for maintaining or even improving accuracy [38]. Tayeh et al. [39] reduced the marker set by two-thirds without losing predictive performance. Due to these factors, fixed low-density arrays with uniform coverage present a cost-effective alternative for smaller breeding programs [21,40].

Another important aspect to consider in plant breeding is that selection is rarely based on a single trait. In most cases, breeders must consider multiple traits of interest simultaneously. One widely used method for multi-trait selection is the selection index [41]. This approach integrates the goals of a breeding program and the desired response to selection across traits into a single index, helping breeders identify genotypes with the highest overall merit [42-43]. Selection indices

are particularly useful when dealing with complex trade-offs, such as the well-known negative correlation between oil and protein content in soybean [44]. Improving both traits simultaneously is a significant challenge, as lines with higher oil content typically have lower protein content. Selection indices can simplify decision-making by converting multiple trait values into a single, interpretable metric. However, defining the appropriate economic weights for each trait remains a challenge. These weights may be based on market prices, breeding priorities, or specific program objectives [1,45-46].

In this paper, we present the above listed factors and their combinations, which are crucial in choosing the best GS strategy to improve the prediction accuracy for selection in a public soybean breeding program. The objectives of this research were: a) to define the best genomic selection (GS) models for predicting yield, protein, oil content, and maturity in soybean breeding programs, while identifying the optimal training population optimization method (structure versus size) and determining the minimum number of genotypes required to compose a training population that ensures high performance in the GS model across multiple locations; b) to make multi-trait selection based on genomic prediction to improve the accuracy of breeding decisions. By addressing these objectives, we aim to enhance the application of GS in soybean breeding.

# Material and methods

## Experimental design

Experiments were conducted in 2023 and 2024, and all relevant information is presented in S1 Table.

## Data collection

Grain yield was measured using a plot combine equipped with sensors for weight and moisture content. Yield was then calculated based on the known plot dimensions. Maturity was assessed visually. During early-stage testing, relative maturity was determined using standard check cultivars grown within each field. Seed oil and protein concentrations were estimated in real time using a near-infrared (NIR) sensor mounted on the combine, and these estimates were validated using a bench-top analyzer at the Ames facility. Seed yield was recorded for all experiments in both years. Maturity data were collected at four locations (Ames, Lucas, Sutherland, and Crawfordsville), while protein and oil data were collected at three locations (Ames, Lucas, and Crawfordsville) each year.

Outliers were identified using both boxplot visualization and standardized residuals greater than ±3 standard deviations from the mean. Observations meeting these criteria were considered errors or inconsistencies and were excluded from subsequent analyses. In total, approximately 1% of the plots were removed. Best Linear Unbiased Predictors (BLUPs) were obtained using model:

$$Y_{ijk} = B_k + G_i + E_j + GE_{ij} + E_{ijk}$$

where:

$Y_{ijk}$ is the phenotypic observation for genotype i, environment j, and replication k;

$B_k$ is the fixed effects of block k with assumption $Ej \sim N(0,\sigma^2_B)$;

$G_i$ is the fixed effect of genotype i;

$E_j$ is the random effect of environment j with assumption $Ej \sim N(0,\sigma^2_E)$;

$GE_{ij}$ is the interaction effect between genotype i and environment j, which is considered random;

$E_{ijk}$ is the random error term with assumption $Ej \sim N(0,\sigma^2)$.

All the mixed model analyses were performed using the package lme4 [47] in R.

## Genomic selection methods

Six genomic selection methods were compared when predicting yield, protein, oil, and maturity in the Iowa State University's soybean breeding program. The six methods used were Ridge Regression Best Linear Unbiased Prediction (RRBLUP), Random Forest (RF), Support Vector Machine (SVM), Reproducing Kernel Hilbert Space (RKHS), Bayes A, and Bayes B.

All the methods were implemented using the R software. The package BGLR [48] was used to implement Bayes A, Bayes B, and RKHS. The package rrBLUP [49] was used to perform RRBLUP, and the package caret [50] was used to perform RF and SVM. All methods were implemented using 5-fold cross-validation with 10 replications, resulting in a total of 384,000 analyses.

## Training population structure

Four training population structures were tested to capture the best prediction accuracy.

Random Selection (RS): This method consists of choosing genotypes to compose the training population completely random from the whole population.

Maturity Group Random Selection (MGRS): This method consists of building the training population by applying stratified random sampling by maturity group, ensuring that the relative frequency of each group in the training set matched its frequency in the entire population (e.g., if MG1 accounted for 5% of the population, it also represented 5% of the training population).

Experiment Random Selection (ERS): To construct the training population, we randomly sampled genotypes within each experiment while preserving the same proportional distribution as

in the entire dataset (e.g., if a given experiment contributed 15% of the total population, it also accounted for 15% of the training set).

Genetic Algorithm (GA): A genetic algorithm was implemented to identify a training set with maximum genetic diversity. The GA simulates natural selection by iteratively generating candidate solutions (subsets of genotypes), applying crossover and mutation operators, and retaining the best-performing subsets across generations. Genetic diversity was quantified as the average pairwise genetic distance among selected individuals, calculated from the marker-based genomic relationship matrix (GRM). The subset with the highest diversity score was retained as the final training population.

# Training population size

We evaluated genomic prediction under different training population sizes, ranging from 10% to 90% of the available genotypes. For each location and year, the complete set of evaluated genotypes was partitioned into training and testing subsets according to the four training population selection strategies described above. The training set (10–90% of genotypes) was used to fit the genomic prediction model, and the remaining genotypes were used as the testing set. Prediction accuracy was then assessed by correlating predicted and observed phenotypes for the withheld genotypes.

# Genomic selection effectiveness

The following statistical parameters were estimated to compare all scenarios (combinations among training population structure, training population size, genomic selection methods, and training population size, as described above):

- Prediction accuracy was calculated as the Pearson correlation coefficient between the validation population for each variable and the genomic estimated breeding value (GEBV).
- Validation coincidence is the percentage between the 10% selected superior individuals based on GEBV in the validation population, and the 10% selected superior individuals based on the phenotypic value in the validation population.

## Statistical analysis

Plateau regression [51] was used to determine the optimal number of genotypes to be evaluated in each location-year combination, without incurring any loss of prediction accuracy.

A three-way ANOVA was conducted to assess the effects of genomic selection method, training population structure, and training population size on prediction accuracy. All effects in the model were treated as fixed, and the analysis followed the model:

$$\text{Accuracy}_{ijkl} = \mu + M_i + T_j + S_k + MT_{ij} + MS_{ik} + TS_{jk} + MTS_{ijk} + \varepsilon_{ijkl}$$

where

$M_i$ represents the genomic selection method,

$T_j$ the training population structure,

$S_k$ the training population size,

and $\varepsilon_{ijkl}$ the residual error.

Tukey's HSD test was applied for post-hoc pairwise comparisons among factor levels. All analyses were performed using R, and the following packages were used: AgroReg [52] for plateau regression, easyanova [53] for ANOVA and Tukey test, and ggplot2 [54] for all graphics. All codes are provided as supplementary material upon request to the authors.

## Optimal number of locations to achieve maximum accuracy

A power analysis was performed to determine the optimal number of locations required to achieve the highest prediction accuracy for yield, protein, oil, and maturity. The parameters used in the power analysis, including genetic variance and heritability, were estimated for each trait using mixed models. To evaluate different levels of genetic diversity, the genotypes were grouped into two to five clusters representing distinct performance categories: (1) Elite Performers, (2) High Performers, (3) Average Performers, (4) Low Performers, and (5) Underperformers. The clustering of genotypes into these groups was conducted using the k-means algorithm.

## Index selection implementation

After testing all combinations of genomic selection models and identifying the best-performing one, we estimated the GEBVs for each line among the entire experimental lines. From this population, the superior 5% of genotypes were selected using five different strategies: (1) direct selection for yield only, (2) direct selection for oil only, (3) direct selection for protein only, (4) a rank-based index, and (5) Smith–Hazel index. For the Smith–Hazel index, the weights were calculated as the genetic standard deviation for each trait.

Response to selection was calculated for yield, protein, and oil across the five selection methods using the formula described by Singh et al. [1]:

$$\Delta G = i * h^2 * \sigma_p$$

where

$\Delta G$ is the expected genetic gain,

i is the selection intensity,

$h^2$ is the trait heritability, $\sigma$

$\sigma_p$ is the phenotypic standard deviation.

Additionally, we evaluated the overall response by considering the simultaneous improvement of the three traits. Specifically, we compared the five selection strategies described above in terms of their ability to maximize genetic gain across yield, protein, and oil, and identified the most effective approach.

All selection index analyses were performed using R (codes are available in the supplementary material and upon the request for the authors.

# Results

## Optimal number of genotypes for training genomic selection models

The minimum number of genotypes (MNG) required to achieve the highest prediction accuracy for each location was determined using plateau regression. For all scenarios, a range from 50% to 90% of the individuals in the training population is necessary to capture the highest accuracy for each location, model, and training population structure combination (Fig 1).

**Fig 1. Minimum percentage of genotypes required in the training population to achieve maximum prediction accuracy for yield, oil, protein, and maturity across different training population structures, genomic selection methods, and locations**. ERS - Experiment random selection, GA - Genetic algorithm, MGRS - Maturity group random selection, RS - Random selection. Six genomic selection models were tested, including Bayes A, Bayes B, Random Forest (RF), Reproducing Kernel Hilbert Space (RKHS), Ridge Regression Best Linear Unbiased Prediction (RRBLUP), and Support Vector Machine (SVM).

Based on the results obtained from the plateau regression analysis, we determined the optimal number of genotypes to be evaluated in each location for effective training of the genomic prediction model. These findings provide valuable insights for refining the breeding strategy, such as increasing the number of testing locations or expanding the number of genotypes evaluated per site.

## Comparing the performance of genomic selection models

Statistically, the Random Forest (RF) and Support Vector Machine (SVM) methods showed the highest prediction accuracy for yield across all locations and training population structures (TPS) (S1-S4 Figs). For protein, SVM consistently outperformed all other genomic selection (GS) methods across locations and TPS, followed by RF (S1-S4 Figs). For oil, both SVM and RF achieved the highest prediction accuracies across all locations and TPS configurations (S1-S4 Figs). Finally, for maturity, RF outperformed the other methods in Lucas and Sutherland, while showing similar performance to SVM in Ames 01 and Crawfordsville (S1-S4 Figs).

## Comparative analysis of training population structure

Lucas, Rockport, and Sutherland consistently exhibited the highest prediction accuracy for yield (Fig 2 and S5-S9 Figs), demonstrating distinct mean values compared to other locations. Meanwhile, Lucas presented the best prediction accuracy for oil (Fig 2 and S5-S9 Figs), for protein (Fig 2 and S5-S9 Figs), and Ames 01 and Crawfordsville for maturity (Fig 2 and S5-S9 Figs). This suggests potential site-specific effects. In contrast, Ames 01 showed the lowest prediction accuracy for yield (Fig 2 and S5-S9 Figs), Crawfordsville for oil and protein (Fig 2 and S5-S9 Figs), and

Lucas for maturity (Fig 2 and S5-S9 Figs), highlighting the influence of locations on prediction outcomes across various traits.

**Fig 2. Prediction accuracy across all locations for yield, protein (Pro), oil, and maturity (Mat) under four training-population structures: Random (random selection), MG (maturity-group random selection), Exp (experimental random selection), and GA (genetic algorithm), using the rrBLUP genomic selection model. Locations followed by different letters differ significantly according to Tukey's test at the 5% significance level**.

Building the training population based on the genetic algorithm was the most effective strategy for improving yield prediction accuracy in almost all locations and across genomic selection models (Fig 3 and S10-S14 Figs). However, for Urbana and Rockport, identifying the Maturity Group random selection (MGRS) structure was the most effective across genomic selection models (Fig 3 and S10-S14 Figs).

**Fig 3. Prediction accuracy across all training population structures for yield, protein (Pro), oil, and maturity (Mat) under eight locations: Ames 01, Ames 02, Crawfordsville, Jerico, Lucas, Rockport, Sutherland, and Urbana, using the rrBLUP genomic selection model. Training population structures followed by different letters differ significantly according to Tukey's test at the 5% significance level**.

In addition, the prediction accuracy for oil and protein was highest for the genetic algorithm training population structure across all locations and genomic selection models (Fig 3 and S10-

S14 Figs). For maturity, the random selection strategy achieved the highest accuracy for Sutherland, Lucas, and Ames 01 (Fig 3 and S10-S14 Figs).

## Optimal number of locations to enhance the best prediction accuracy

The parameters used for power analysis are presented in Table 1. The results indicate that when genotypes are divided into two groups, at least eight locations are required to achieve a prediction accuracy greater than 0.80 for yield, ten locations for oil and protein, and more than ten for maturity (Fig 4). When the population is partitioned into three groups, the required number of locations decreases to seven for yield, oil, and maturity, and six for protein. For four groups, six locations are sufficient for yield, and five locations are needed for oil, protein, and maturity. When genotypes are further divided into five groups, only five locations are required to achieve the desired prediction accuracy for all evaluated traits.

**Table 1**. Genetic parameters for the four traits were evaluated at eight locations. $\sigma_G^2$- genetic variance, $\sigma_{GE}^2$ – geneticXenvironment variance, $\sigma^2$- residual variance, $H^2$- heritability.

| Trait | $\sigma_G^2$ | $\sigma_{GE}^2$ | $\sigma^2$ | $H^2$ |
|---|---|---|---|---|
| **Yield** | 9.17 | 10.20 | 21.28 | 0.30 |
| **Oil** | 0.32 | 0.04 | 0.04 | 0.89 |
| **Pro** | 1.09 | 0.17 | 0.07 | 0.94 |
| **Mat** | 0.15 | 0.02 | 0.01 | 0.83 |

**Fig 4**. **Number of locations needed to get prediction accuracy greater than 0.8 for yield, oil, protein, and maturity**.

## Selection index implementation

Five selection strategies were evaluated to identify genotypes that can simultaneously optimize yield, oil content, and protein content. The results demonstrate that achieving simultaneous positive gains for all three traits is not feasible, regardless of the strategy applied (Fig 5). Among the evaluated approaches, the Rank Index showed the best overall performance, generating positive selection gains for both yield and oil content. However, as expected due to the known trade-offs among these traits, it produced a negative gain for protein content. Even so, the decline in protein was less pronounced than that observed for the other strategies, except when protein itself was used as the direct selection criterion.

**Fig 5. Genetic gain estimates for grain yield, oil content, and protein content obtained through five selection strategies: direct selection for yield, direct selection for oil, direct selection for protein, rank-based index, and Smith-Hazel index. Gains were calculated based on GEBVs derived from the optimal genomic prediction model**.

# Discussion

## Optimal number of genotypes for training genomic selection models

The determination of the minimum number of genotypes (MNG) required to achieve the highest prediction accuracy for each location using plateau regression revealed several important

insights. Overall, the results demonstrate the need for location-specific optimization in soybean genomic selection. Across most locations, using 60% to 80% of the genotypes in the training population was sufficient to identify the MNG needed to maximize prediction accuracy across different training population structures and GS methods (Fig 1). However, substantial variability in MNG was observed among locations and models. For instance, Jerico, Lucas, and Urbana required at least 80% of the individuals in the training population to reach peak accuracy in nearly all scenarios, emphasizing the influence of environmental and genetic context on model performance. In contrast, locations such as Ames01 and Lucas displayed relatively low MNG values, indicating that fewer genotypes were needed to achieve high predictive accuracy compared with other environments. This pattern may reflect a higher inherent predictive power at these sites, enabling accurate modeling with smaller training populations [55].

The completely random strategy for most locations generally required a lower MNG than maturity group randomization and the genetic algorithm. This finding suggests that simpler population structures can be effective in certain contexts, potentially reducing the complexity and cost of breeding programs. However, it is also essential to consider genetic diversity and specific traits of interest when selecting the most appropriate strategy [56-58]. Similarly, the results suggest that reducing the number of genotypes evaluated for each location is possible without compromising prediction accuracy. This reduction is significant as it allows for reallocating resources towards expanding the number of locations or increasing the evaluation of critical traits such as disease resistance, iron deficiency, and lodging resistance. By optimizing the number of genotypes, breeding programs can enhance their efficiency and effectiveness, ultimately accelerating the development of improved soybean varieties. Again, the caveat is that it may lead to a genetic bottleneck or reduction in useful genetic variation, leading to a negative outcome for

genetic gain in the medium to long term [55-56]. Efficiently designed training populations improve prediction accuracy and ensure that breeding programs are cost-effective and sustainable in the long term [21-22].

## Comparative analysis of training population structure

The accuracy of genomic selection models tends to decrease as the training and test populations diverge [32], which is why it is crucial to identify the most effective strategies for each trait to enhance selection efficiency. The comparative analysis of training population structure and GS methods provided critical insights into optimizing soybean breeding programs. The experimental random selection (ERS) was the best training population structure across all locations and genomic selection methods.

Results from this work helped develop guidelines and establish a benchmark of a wide range of training set optimization methods under different population structures, genetic architectures, and heritability values, in the context of a public breeding program. An ideal training set optimization method should (i) be able to build a training set that maximizes the accuracy of the GS model by maximizing the relationship between the training set and the test set and by minimizing the relationship within the training set to capture as much genetic variance as possible. [59-60], (ii) reduce the computational burden, and iii) be easy to implement [55, 61-63]. We report that the training set has a very significant impact on increasing the accuracy of GS models relative to random sampling [64]. These findings are remarkable given that the experiment random selection (ERS), which involves straightforward selection without specific rules, proved effective. This contrasts with the stratified method, which requires proportional representation by maturity

group, and the genetic algorithm, which is more time-consuming and computationally intensive. Despite the simplicity of the ERS, it performed comparably to the more complex methods.

With the advent of GS as a widespread breeding tool, mechanisms to efficiently design an optimal training set for GS models became more relevant, since they allow maximizing the accuracy while minimizing the phenotyping costs [64]. It has been demonstrated that selecting an optimized training set is a crucial factor for accurate genomic predictions [33, 65-67]. The optimization aims to maximize the accuracy of the predictions made on a test set while minimizing the size of the training set [68], which reduces phenotyping costs or uses these resources to evaluate other essential traits that cannot be evaluated right now, such as diseases, drought, heat, and flooding.

With the implementation of genomic-assisted breeding tools into breeding programs, the evaluation of germplasm has undergone significant changes. Breeders do not need to evaluate the entire progeny in the field, but rather to optimally select a training set size that will improve the resource allocation within the breeding program. For instance, a reduction in training set size due to optimization implies that more alleles could be tested in the field or that more resources could be invested in more precise phenotyping. Consequently, it is important to optimize the training set size to minimize it without incurring a large loss of accuracy. However, the training set size optimization is computationally intensive as it relies on performing optimization repeatedly for several training set sizes and fitting a function that can predict the evolution of the evaluation criteria as the training set size varies [64].

Our results indicate that all GS methods were statistically similar, except for SVM. RRBLUP emerged as the optimal GS method, while the ERS proved to be the most effective training population structure. Implementing this approach across all locations promises enhanced

selection accuracy within the soybean breeding program while optimizing time efficiency. The performances of the different models were also tested by Fernandez-Gonzales et al. [64], and they found them very similar across datasets and different training sets and they recommended simple GS models, such as RRBLUP and GBLUP, as these perform similarly compared to complex models, such as Bayesian models and some machine learning models.

Overall, our study underscores the necessity of location-specific optimization in GS for soybean [69-70]. The identification of the minimum number of genotypes required for optimal prediction accuracy provides a framework for improving the efficiency of breeding programs. Future research should continue to refine these strategies, focusing on increasing genotypic diversity and sample sizes at locations where current methods are insufficient.

## Selection index implementation

In this study, five different selection strategies were evaluated to select genotypes that simultaneously improve grain yield, oil content, and protein content. The results demonstrated that achieving positive selection gains across all three traits simultaneously is not feasible, regardless of the strategy employed, suggesting that trade-offs between traits are inherent when selecting for multiple complex traits [1,71]. While some strategies performed better for specific traits, no single method allowed for the simultaneous improvement of yield, oil content, and protein content, highlighting the difficulty in achieving balanced genetic gains across multiple objectives. Among the five strategies tested, the Rank index proved to be the most promising approach, yielding positive genetic gains in both yield and oil content. While it resulted in a negative response to selection for protein content, the magnitude of the reduction was lower than other methods, indicating its usefulness. In the soybean breeding programs, several contrasting breeding

objectives may be targets, such as traits that have a negative correlation (e.g., yield and protein) or are linked in repulsion. Breeders may be targeting these three most important traits, and each trait will be under polygenic control, leading to more complexity in assembling the desirable combination and an ability to identify that combination [1]. In addition, we can utilize genomic selection to achieve an effective selection outcome for more complex traits, as demonstrated in this paper. DePauw et al. [71] provided a detailed description of negative correlations and breeding strategies in wheat, essentially establishing that traits with negative correlations must be selected simultaneously. While the progress is slower, over the longer term, even grain yield and protein correlations were positively shifted. The Rank index outperformed the direct selection strategies for each individual trait, suggesting that multi-trait selection approaches may be more effective when aiming for overall improvement. Moreover, such indices can be readily expanded to include additional traits of increasing relevance to soybean breeding programs, such as heat tolerance, which has become a critical target in the U.S. Midwest due to rising temperatures and increased climate variability [72].

A crucial consideration in implementing GS is the necessity to maintain genetic diversity in the selection of parents and breeding populations [73-74]. While selecting specific traits can drive genetic improvement, preserving the variability of the gene pool is crucial to avoid narrowing the genetic diversity. Genetic diversity can be measured using phenotypic or molecular tools; however, it is crucial to consider the diversity of the genetic basis of multiple traits. For example, yield, protein, oil, abiotic and biotic tolerance, quality traits, and more [75-77]. Genetic diversity enables more adaptive responses to changing environmental conditions and reduces the risk of selecting undesirable alleles that could negatively impact broader breeding objectives [78]. The

breeding program should consider incorporating diversity management strategies to overcome these challenges.

In this context, phenomic selection emerges as a valuable complementary approach to GS. High-throughput phenotyping enables the collection of detailed, multi-trait phenotypic data across environments and developmental stages, capturing complex plant responses that may not be fully explained by genomic data alone [79]. By integrating phenomic information with GS models, breeders can improve prediction accuracy, better account for genotype × environment interactions, and enhance the selection of superior genotypes while maintaining genetic diversity. Moreover, phenomic selection can support the identification of complementary parental combinations, thereby contributing to increased genetic gain without excessively narrowing the genetic base of breeding populations.

# Conclusion

The most effective strategy for implementing genomic selection in a public soybean breeding program is to use the RRBLUP model. To maximize predictive performance, the model should be trained with approximately 80% of the total population, ensuring robust and reliable estimates. Incorporating experimental random selection to assemble the training population further increases genetic diversity, which is essential for improving prediction accuracy and maintaining consistent performance across breeding cycles. Together, these practices provide a strong foundation for maximizing genetic gains within the program.

The Rank Index is also a highly effective strategy for selecting soybean genotypes, especially when the goal is to improve multiple traits simultaneously. By integrating information from several traits, this method enables more balanced and comprehensive genetic progress than

single-trait selection approaches, making it a valuable tool for breeding programs aiming to enhance overall productivity and quality.


# Acknowledgments

The authors thank the students, scholars and staff in A. K. Singh group for their assistance with data collection. Our special thanks to Jennifer Hicks and Brian Scott for their efforts on this project.


# References


1. Singh DP, Singh AK, Singh A. Plant breeding and cultivar development. Academic Press; 2021 Jan 21.
2. Bhat JA, Ali S, Salgotra RK, Mir ZA, Dutta S, Jadon V, Tyagi A, Mushtaq M, Jain N, Singh PK, Singh GP. Genomic selection in the era of next generation sequencing for complex traits in plant breeding. Frontiers in genetics. 2016 Dec 27;7:221.
3. Jackson R, Buntjer JB, Bentley AR, Lage J, Byrne E, Burt C, Jack P, Berry S, Flatman E, Poupard B, Smith S. Phenomic and genomic prediction of yield on multiple locations in winter wheat. Frontiers in genetics. 2023 May 9;14:1164935.
4. Xu Y, Liu X, Fu J, Wang H, Wang J, Huang C, Prasanna BM, Olsen MS, Wang G, Zhang A. Enhancing genetic gain through genomic selection: from livestock to plants. Plant Communications. 2020 Jan 13;1(1).
5. Zhou J, Zhou J, Ye H, Ali ML, Chen P, Nguyen HT. Yield estimation of soybean breeding lines under drought stress using unmanned aerial vehicle-based imagery and convolutional neural network. Biosystems Engineering. 2021 Apr 1;204:90-103.

6. Lin F, Chhapekar SS, Vieira CC, Da Silva MP, Rojas A, Lee D, Liu N, Pardo EM, Lee YC, Dong Z, Pinheiro JB. Breeding for disease resistance in soybean: a global perspective. Theoretical and Applied Genetics. 2022 Nov;135(11):3773-872.

7. Anilkumar C, Sunitha NC, Harikrishna, Devate NB, Ramesh S. Advances in integrated genomic selection for rapid genetic gain in crop improvement: a review. Planta. 2022 Nov;256(5):87.

8. Bandillo NB, Jarquin D, Posadas LG, Lorenz AJ, Graef GL. Genomic selection performs as effectively as phenotypic selection for increasing seed yield in soybean. The Plant Genome. 2023 Mar;16(1):e20285.

9. Meuwissen TH, Hayes BJ, Goddard M. Prediction of total genetic value using genome-wide dense marker maps. genetics. 2001 Apr 1;157(4):1819-29.

**10.** Zuffo AM, Steiner F, Aguilera JG, Teodoro PE, Teodoro LP, Busch A. Multi-trait stability index: A tool for simultaneous selection of soya bean genotypes in drought and saline stress. Journal of Agronomy and Crop Science. 2020 Dec;206(6):815-22.

11. Voss-Fels KP, Cooper M, Hayes BJ. Accelerating crop genetic gains with genomic selection. Theoretical and Applied Genetics. 2019 Mar 1;132(3):669-86.

12. Ravelombola W, Qin J, Shi A, Song Q, Yuan J, Wang F, Chen P, Yan L, Feng Y, Zhao T, Meng Y. Genome-wide association study and genomic selection for yield and related traits in soybean. PLoS One. 2021 Aug 13;16(8):e0255761.

13. Toda Y, Kaga A, Kajiya-Kanegae H, Hattori T, Yamaoka S, Okamoto M, Tsujimoto H, Iwata H. Genomic prediction modeling of soybean biomass using UAV-based remote sensing and longitudinal model parameters. The Plant Genome. 2021 Nov;14(3):e20157.

14. Wartha CA, Lorenz AJ. Genomic predictions of genetic variances and correlations among traits for breeding crosses in soybean. Heredity. 2024 Sep;133(3):173-85.

15. Qin J, Wang F, Zhao Q, Shi A, Zhao T, Song Q, Ravelombola W, An H, Yan L, Yang C, Zhang M. Identification of candidate genes and genomic selection for seed protein in soybean breeding pipeline. Frontiers in Plant Science. 2022 Jun 16;13:882732.

16. Ravelombola WS, Qin J, Shi A, Nice L, Bao Y, Lorenz A, Orf JH, Young ND, Chen S. Genome-wide association study and genomic selection for soybean chlorophyll content associated with soybean cyst nematode tolerance. BMC genomics. 2019 Dec;20:1-8.

17. Al-Mamun HA, Danilevicz MF, Marsh JI, Gondro C, Edwards D. Exploring genomic feature selection: A comparative analysis of GWAS and machine learning algorithms in a large-scale soybean dataset. The Plant Genome. 2024 Sep 10:e20503.

18. Miller MJ, Song Q, Li Z. Genomic selection of soybean (Glycine max) for genetic improvement of yield and seed composition in a breeding context. The Plant Genome. 2023 Dec;16(4):e20384.

19. Rolling WR, Dorrance AE, McHale LK. Testing methods and statistical models of genomic prediction for quantitative disease resistance to Phytophthora sojae in soybean [Glycine max (L.) Merr] germplasm collections. Theoretical and Applied Genetics. 2020 Dec;133(12):3441-54.

20. Fernández-González J, Akdemir D, Isidro y Sánchez J. A comparison of methods for training population optimization in genomic selection. Theoretical and Applied Genetics. 2023 Mar;136(3):30.

21. Spindel J, Begum H, Akdemir D, Virk P, Collard B, Redona E, Atlin G, Jannink JL, McCouch SR. Genomic selection and association mapping in rice (Oryza sativa): effect of trait genetic architecture, training population composition, marker number and statistical model on

accuracy of rice genomic selection in elite, tropical rice breeding lines. PLoS genetics. 2015 Feb 17;11(2):e1004982.

22. De Azevedo Peixoto L, Moellers TC, Zhang J, Lorenz AJ, Bhering LL, Beavis WD, Singh AK. Leveraging genomic prediction to scan germplasm collection for crop improvement. PloS one. 2017 Jun 9;12(6):e0179191.

23. Silva ÉD, Xavier A, Faria MV. Impact of genomic prediction model, selection intensity, and breeding strategy on the long-term genetic gain and genetic erosion in soybean breeding. Frontiers in Genetics. 2021 Sep 1;12:637133.

24. Habier D, Fernando RL, Dekkers J. The impact of genetic relationship information on genome-assisted breeding values. Genetics. 2007 Dec 1;177(4):2389-97.

25. Norman A, Taylor J, Edwards J, Kuchel H. Optimising genomic selection in wheat: effect of marker density, population size and population structure on prediction accuracy. G3: Genes, Genomes, Genetics. 2018 Sep 1;8(9):2889-99.

26. Crossa J, Perez P, Hickey J, Burgueno J, Ornella L, Cerón-Rojas J, Zhang X, Dreisigacker S, Babu R, Li Y, Bonnett D. Genomic prediction in CIMMYT maize and wheat breeding programs. Heredity. 2014 Jan;112(1):48-60.

27. Rincent R, Laloë D, Nicolas S, Altmann T, Brunel D, Revilla P, Rodriguez VM, Moreno-Gonzalez J, Melchinger A, Bauer E, Schoen CC. Maximizing the reliability of genomic selection by optimizing the calibration set of reference individuals: comparison of methods in two diverse groups of maize inbreds (Zea mays L.). Genetics. 2012 Oct 1;192(2):715-28.

28. Isidro J, Jannink JL, Akdemir D, Poland J, Heslot N, Sorrells ME. Training set optimization under population structure in genomic selection. Theoretical and applied genetics. 2015 Jan;128(1):145-58.

29. Zhang Z, Zhang Q, Ding X. Advances in genomic selection in domestic animals. Chinese science bulletin. 2011 Sep;56(25):2655-63.

30. Wang X, Xu Y, Hu Z, Xu C. Genomic selection methods for crop improvement: Current status and prospects. The Crop Journal. 2018 Aug 1;6(4):330-40.

31. Akdemir D, Beavis W, Fritsche-Neto R, Singh AK, Isidro-Sánchez J. Multi-objective optimized genomic breeding strategies for sustainable food improvement. Heredity. 2019 May;122(5):672-83.

32. Akdemir D, Sanchez JI, Jannink JL. Optimization of genomic selection training populations with a genetic algorithm. Genetics Selection Evolution. 2015 Dec;47:1-0.

33. Akdemir D, Isidro-Sánchez J. Design of training populations for selective phenotyping in genomic prediction. Scientific reports. 2019 Feb 5;9(1):1446.

34. Ramasubramanian V, Beavis WD. Strategies to assure optimal trade-offs among competing objectives for the genetic improvement of soybean. Frontiers in Genetics. 2021 Sep 24;12:675500.

35. Gorjanc G, Cleveland MA, Houston RD, Hickey JM. Potential of genotyping-by-sequencing for genomic selection in livestock populations. Genetics Selection Evolution. 2015 Mar 1;47(1):12.

36. Habier D, Fernando RL, Dekkers JC. Genomic selection using low-density marker panels. Genetics. 2009 May 1;182(1):343-53.

37. Ma Y, Reif JC, Jiang Y, Wen Z, Wang D, Liu Z, Guo Y, Wei S, Wang S, Yang C, Wang H. Potential of marker selection to increase prediction accuracy of genomic selection in soybean (Glycine max L.). Molecular Breeding. 2016 Aug;36(8):113.

38. Hoffstetter A, Cabrera A, Huang M, Sneller C. Optimizing training population data and validation of genomic selection for economic traits in soft winter wheat. G3: Genes, Genomes, Genetics. 2016 Sep 1;6(9):2919-28.

39. Tayeh N, Klein A, Le Paslier MC, Jacquin F, Houtin H, Rond C, Chabert-Martinello M, Magnin-Robert JB, Marget P, Aubert G, Burstin J. Genomic prediction in pea: effect of marker density and training population size and composition on prediction accuracy. Frontiers in Plant Science. 2015 Nov 17;6:941.

40. Thomson MJ. High-throughput SNP genotyping to accelerate crop improvement. Plant Breeding and Biotechnology. 2014 Sep 30;2(3):195-212.

41. Walsh B, Lynch M. Evolution and selection of quantitative traits. Oxford University Press; 2018 Jun 21.

42. Bauer AM, Léon J. Multiple-trait breeding values for parental selection in self-pollinating crops. Theoretical and Applied Genetics. 2008 Jan;116(2):235-42.

43. Schulthess AW, Wang Y, Miedaner T, Wilde P, Reif JC, Zhao Y. Multiple-trait-and selection indices-genomic predictions for grain yield and protein content in rye for feeding purposes. Theoretical and Applied Genetics. 2016 Feb;129(2):273-87.

44. Simmonds NW. The relation between yield and protein in cereal grain. Journal of the Science of Food and Agriculture. 1995 Mar;67(3):309-15.

45. Haile JK, N'Diaye A, Clarke F, Clarke J, Knox R, Rutkoski J, Bassi FM, Pozniak CJ. Genomic selection for grain yield and quality traits in durum wheat. Molecular breeding. 2018 Jun;38(6):75.

46. Heffner EL, Jannink JL, Iwata H, Souza E, Sorrells ME. Genomic selection accuracy for grain quality traits in biparental wheat populations. Crop Science. 2011 Nov;51(6):2597-606.

47. Bates D, Mächler M, Bolker B, Walker S (2015) Fitting Linear Mixed-Effects Models Using lme4. Journal of Statistical Software 67(1): 1–48.

48. Perez, P., and de los Campos, G., 2014 Genome-Wide Regression and Prediction with the BGLR Statistical Package. Genetics 198 (2): 483-495.

49. Endelman, J.B. 2011. Ridge regression and other kernels for genomic selection with R package rrBLUP. Plant Genome 4:250-255.

50. Kuhn, M. (2008). Building Predictive Models in R Using the caret Package. Journal of Statistical Software, 28(5), 1–26.

51. Bhering LL, de Almeida Filho JE, de Azevedo Peixoto L, Laviola BG, Gomes BE. Plateau regression reveals that eight plants per accession are representative for Jatropha germplasm bank. Industrial Crops and Products. 2015 Mar 1;65:210-5.

52. Shimizu G, Goncalves L (2024). AgroReg: Regression Analysis Linear and Nonlinear for Agriculture. R package version 1.2.10, <https://CRAN.R-project.org/package=AgroReg>.

53. Arnhold, E. Package in the R environment for analysis of variance and complementary analyses. Brazilian Journal of Veterinary Research and Animal Science, v.50, n.6, p.488-492, 2013.

54. Wickham, H. ggplot2: Elegant Graphics for Data Analysis. Springer-Verlag New York, 2016.

55. Merrick LF, Herr AW, Sandhu KS, Lozada DN, Carter AH. Optimizing plant breeding programs for genomic selection. Agronomy. 2022 Mar 16;12(3):714.

56. Lorenz A, Nice L. Training population design and resource allocation for genomic selection in plant breeding. InGenomic selection for crop improvement: new molecular breeding strategies for crop improvement 2017 Nov 1 (pp. 7-22). Cham: Springer International Publishing.

57. Belamkar V, Guttieri MJ, Hussain W, Jarquín D, El-Basyoni I, Poland J, Lorenz AJ, Baenziger PS. Genomic selection in preliminary yield trials in a winter wheat breeding program. G3: Genes, Genomes, Genetics. 2018 Aug 1;8(8):2735-47.

58. Merrick LF, Carter AH. Comparison of genomic selection models for exploring predictive ability of complex traits in breeding programs. The Plant Genome. 2021 Nov;14(3):e20158.

59. Pszczola M, Strabel T, Van Arendonk JA, Calus MP. The impact of genotyping different groups of animals on accuracy when moving from traditional to genomic selection. Journal of dairy science. 2012 Sep 1;95(9):5412-21.

60. Isidro y Sánchez J, Akdemir D. Training set optimization for sparse phenotyping in genomic selection: A conceptual overview. Frontiers in Plant Science. 2021 Sep 9;12:715910.

61. Alemu A, Åstrand J, Montesinos-Lopez OA, y Sanchez JI, Fernandez-Gonzalez J, Tadesse W, Vetukuri RR, Carlsson AS, Ceplitis A, Crossa J, Ortiz R. Genomic selection in plant breeding: Key factors shaping two decades of progress. Molecular Plant. 2024 Apr 1;17(4):552-78.

62. Jeon D, Kang Y, Lee S, Choi S, Sung Y, Lee TH, Kim C. Digitalizing breeding in plants: A new trend of next-generation breeding based on genomic prediction. Frontiers in Plant Science. 2023 Jan 19;14:1092584.

63. Sinha D, Maurya AK, Abdi G, Majeed M, Agarwal R, Mukherjee R, Ganguly S, Aziz R, Bhatia M, Majgaonkar A, Seal S. Integrated genomic selection for accelerating breeding programs of climate-smart cereals. Genes. 2023 Jul 21;14(7):1484.

64. Fernández-González J, Akdemir D, Isidro y Sánchez J. A comparison of methods for training population optimization in genomic selection. Theoretical and Applied Genetics. 2023 Mar;136(3):30.

65. Zhong S, Dekkers JC, Fernando RL, Jannink JL. Factors affecting accuracy from genomic selection in populations derived from multiple inbred lines: a barley case study. Genetics. 2009 May 1;182(1):355-64.

66. Lorenz AJ, Smith KP. Adding genetically distant individuals to training populations reduces genomic prediction accuracy in barley. Crop science. 2015 Nov;55(6):2657-67.

67. Zhang H, Yin L, Wang M, Yuan X, Liu X. Factors affecting the accuracy of genomic selection for agricultural economic traits in maize, cattle, and pig populations. Frontiers in genetics. 2019 Mar 14;10:189.

68. Rio S, Akdemir D, Carvalho T, Sanchez JI. Assessment of genomic prediction reliability and optimization of experimental designs in multi-environment trials. Theoretical and Applied Genetics. 2022 Feb;135(2):405-19.

69. Volpato L, Alves RS, Teodoro PE, Vilela de Resende MD, Nascimento M, Nascimento AC, Ludke WH, Lopes da Silva F, Borém A. Multi-trait multi-environment models in the genetic selection of segregating soybean progeny. PloS one. 2019 Apr 18;14(4):e0215315.

70. Đorđević V, Ćeran M, Miladinović J, Balešević-Tubić S, Petrović K, Miladinov Z, Marinković J. Exploring the performance of genomic prediction models for soybean yield using different validation approaches. Molecular Breeding. 2019 May;39:1-5.

71. DePauw RM, Knox RE, Clarke FR, Wang H, Fernandez MR, Clarke JM, McCaig TN. Shifting undesirable correlations. Euphytica. 2007 Oct;157(3):409-15.

72. Van der Laan L, de Azevedo Peixoto L, Singh AK. Genetic dissection of heat stress tolerance in soybean through genome-wide association studies and use of genomic prediction to enhance breeding applications. npj Science of Plants. 2025 Nov 3;1(1):9.

73. Wartha CA, Campbell BW, Ramasubramanian V, Nice L, Brock A, Cai G, Eskandari MM, Graef G, Hudson ME, Hyten D, Mahan AL. Genomic analysis and predictive modeling in the Northern Uniform Soybean Tests. Crop Science. 2025 Sep;65(5):e70138.

74. Shook JM, Lourenco D, Singh AK. PATRIOT: A pipeline for tracing identity-by-descent for chromosome segments to improve genomic prediction in self-pollinating crop species. Frontiers in plant science. 2021 Sep 29;12:676269.

75. Tayade R, Imran M, Ghimire A, Khan W, Nabi RB, Kim Y. Molecular, genetic, and genomic basis of seed size and yield characteristics in soybean. Frontiers in plant science. 2023 Nov 15;14:1195210.

76. Kumawat G, Raghuvanshi R, Vennampally N, Maranna S, Rajesh V, Chandra S, Kumar S, Rajput LS, Meena LK, Choyal P, Ratnaparkhe MB. Integrating germplasm diversity and omics science to enhance biotic stress resistance in soybean. InGenomics-aided Breeding Strategies for Biotic Stress in Grain Legumes 2024 Aug 14 (pp. 327-358). Singapore: Springer Nature Singapore.

77. Momen S, Sawant S, Fallen BD, Girard AL. Effects of genetic diversity on physicochemical and functional properties of soybean proteins. Journal of Agriculture and Food Research. 2025 Jun 1;21:101878.

78. Hou J, Liu M, Yang K, Liu B, Liu H, Liu J. Genetic variation for adaptive evolution in response to changed environments in plants. Journal of Integrative Plant Biology. 2025 Sep;67(9):2265-93.

79. Van der Laan L, Parmley K, Saadati M, Pacin HT, Panthulugiri S, Sarkar S, Ganapathysubramanian B, Lorenz A, Singh AK. Genomic and phenomic prediction for soybean seed yield, protein, and oil. The Plant Genome. 2025 Mar;18(1):e70002.

# Supporting information

**S1 Table**. Information about the 16 locations used to perform the genomic selection methods.

**S1 Fig.** Prediction accuracy of all genomic selection models across all locations under the Random selection training-population structure strategy, using 80% of the individuals for model training. Genomic selection models followed by different letters differ significantly according to Tukey's test at the 5% significance level.

**S2 Fig.** Prediction accuracy of all genomic selection models across all locations under the Maturity group random selection training-population structure strategy, using 80% of the individuals for model training. Genomic selection models followed by different letters differ significantly according to Tukey's test at the 5% significance level.

**S3 Fig.** Prediction accuracy of all genomic selection models across all locations under the Experiment random selection training-population structure strategy, using 80% of the individuals for model training. Genomic selection models followed by different letters differ significantly according to Tukey's test at the 5% significance level.

**S4 Fig.** Prediction accuracy of all genomic selection models across all locations under the Genetic Algorithm training-population structure strategy, using 80% of the individuals for model training. Genomic selection models followed by different letters differ significantly according to Tukey's test at the 5% significance level.

**S5 Fig.** Prediction accuracy across all locations for yield, protein (Pro), oil, and maturity (Mat) under four training-population structures: Random (random selection), MG (maturity-group random selection), Exp (experimental random selection), and GA (genetic algorithm), using the Bayes A genomic selection model. Locations followed by different letters differ significantly according to Tukey's test at the 5% significance level.

**S6 Fig.** Prediction accuracy across all locations for yield, protein (Pro), oil, and maturity (Mat) under four training-population structures: Random (random selection), MG (maturity-group random selection), Exp (experimental random selection), and GA (genetic algorithm), using the Bayes B genomic selection model. Locations followed by different letters differ significantly according to Tukey's test at the 5% significance level.

**S7 Fig.** Prediction accuracy across all locations for yield, protein (Pro), oil, and maturity (Mat) under four training-population structures: Random (random selection), MG (maturity-group random selection), Exp (experimental random selection), and GA (genetic algorithm), using the RKHS genomic selection model. Locations followed by different letters differ significantly according to Tukey's test at the 5% significance level.

**S8 Fig.** Prediction accuracy across all locations for yield, protein (Pro), oil, and maturity (Mat) under four training-population structures: Random (random selection), MG (maturity-group random selection), Exp (experimental random selection), and GA (genetic algorithm), using the

Random forest genomic selection model. Locations followed by different letters differ significantly according to Tukey’s test at the 5% significance level.

**S9 Fig.** Prediction accuracy across all locations for yield, protein (Pro), oil, and maturity (Mat) under four training-population structures: Random (random selection), MG (maturity-group random selection), Exp (experimental random selection), and GA (genetic algorithm), using the support vector machine genomic selection model. Locations followed by different letters differ significantly according to Tukey’s test at the 5% significance level.

**S10 Fig.** Prediction accuracy across all training population structures for yield, protein (Pro), oil, and maturity (Mat) under eight locations: Ames 01, Ames 02, Crawfordsville, Jerico, Lucas, Rockport, Sutherland, and Urbana, using the Bayes A genomic selection model. Training population structures followed by different letters differ significantly according to Tukey’s test at the 5% significance level.

**S11 Fig.** Prediction accuracy across all training population structures for yield, protein (Pro), oil, and maturity (Mat) under eight locations: Ames 01, Ames 02, Crawfordsville, Jerico, Lucas, Rockport, Sutherland, and Urbana, using the Bayes B genomic selection model. Training population structures followed by different letters differ significantly according to Tukey’s test at the 5% significance level.

**S12 Fig.** Prediction accuracy across all training population structures for yield, protein (Pro), oil, and maturity (Mat) under eight locations: Ames 01, Ames 02, Crawfordsville, Jerico, Lucas,

Rockport, Sutherland, and Urbana, using the RKHS genomic selection model. Training population structures followed by different letters differ significantly according to Tukey's test at the 5% significance level.

**S13 Fig.** Prediction accuracy across all training population structures for yield, protein (Pro), oil, and maturity (Mat) under eight locations: Ames 01, Ames 02, Crawfordsville, Jerico, Lucas, Rockport, Sutherland, and Urbana, using the Random Forest genomic selection model. Training population structures followed by different letters differ significantly according to Tukey's test at the 5% significance level.

**S14 Fig.** Prediction accuracy across all training population structures for yield, protein (Pro), oil, and maturity (Mat) under eight locations: Ames 01, Ames 02, Crawfordsville, Jerico, Lucas, Rockport, Sutherland, and Urbana, using the Support Vector Machine genomic selection model. Training population structures followed by different letters differ significantly according to Tukey's test at the 5% significance level.

Fig 1

Click here to access/download;Figure;Fig 1.tiff

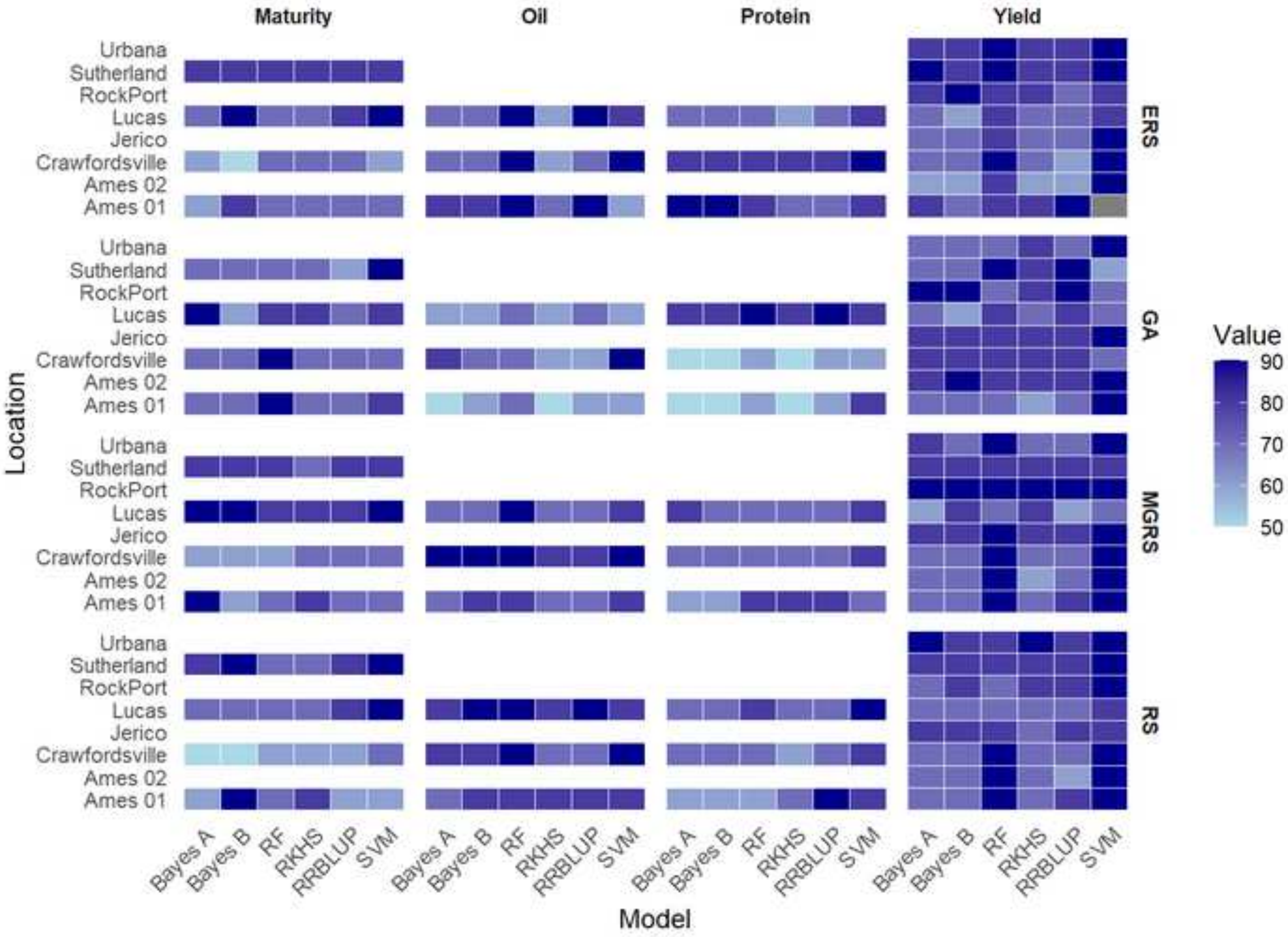

Fig 2

Click here to access/download;Figure;Fig 2.tiff

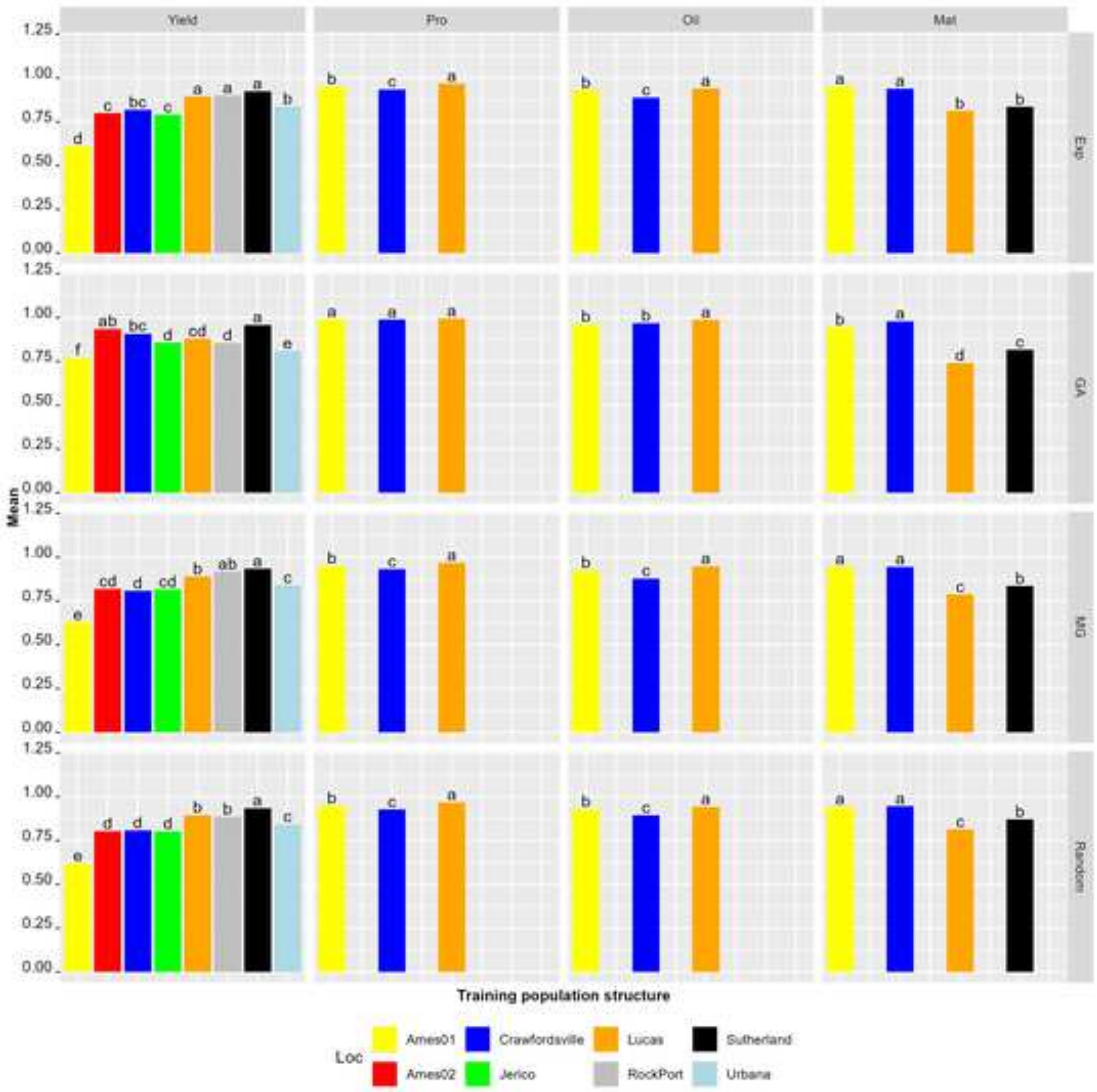

Fig 3

Click here to access/download;Figure;Fig 3.tiff

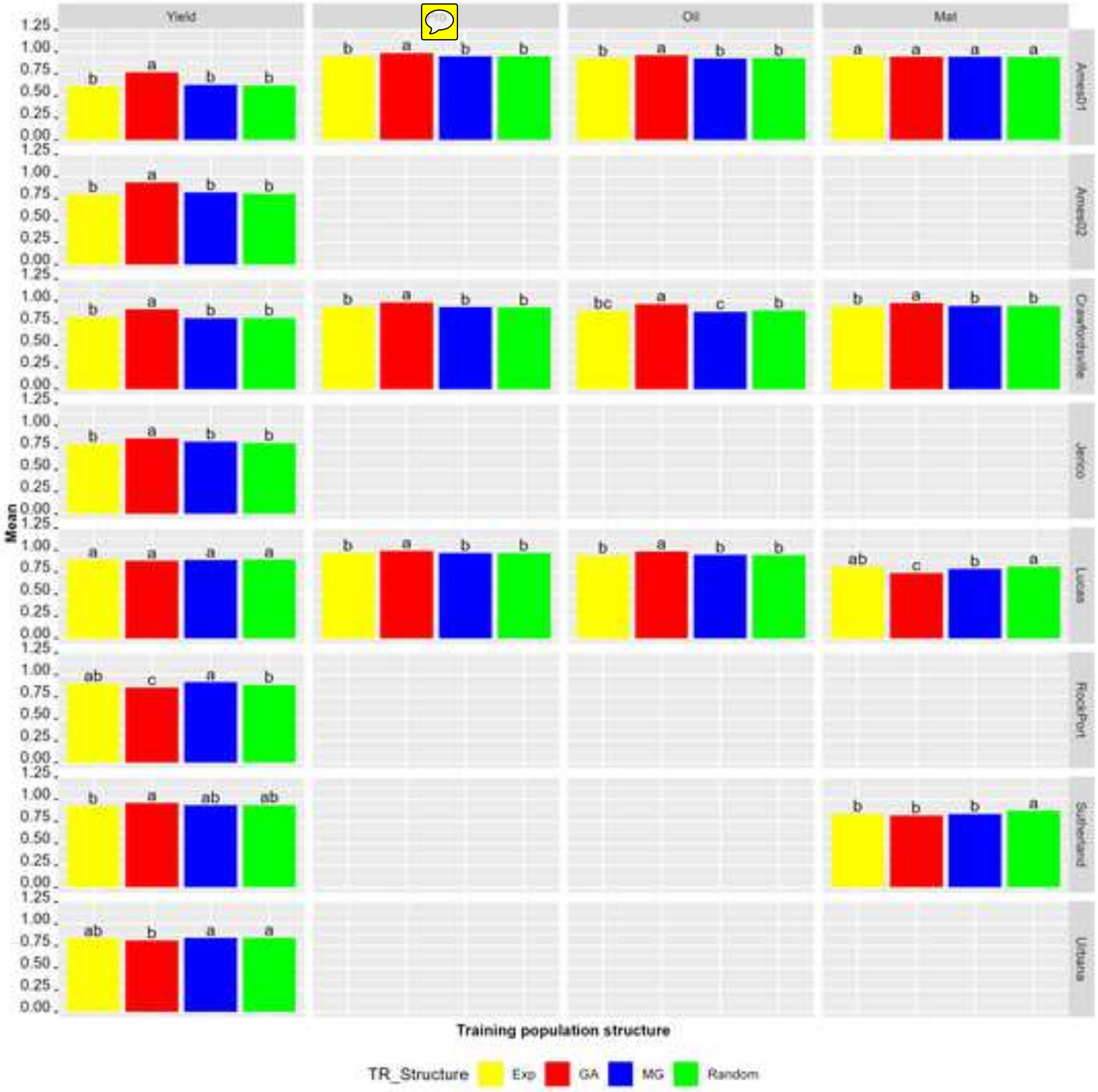

 Click here to access/download;Figure;Fig 4.tiff

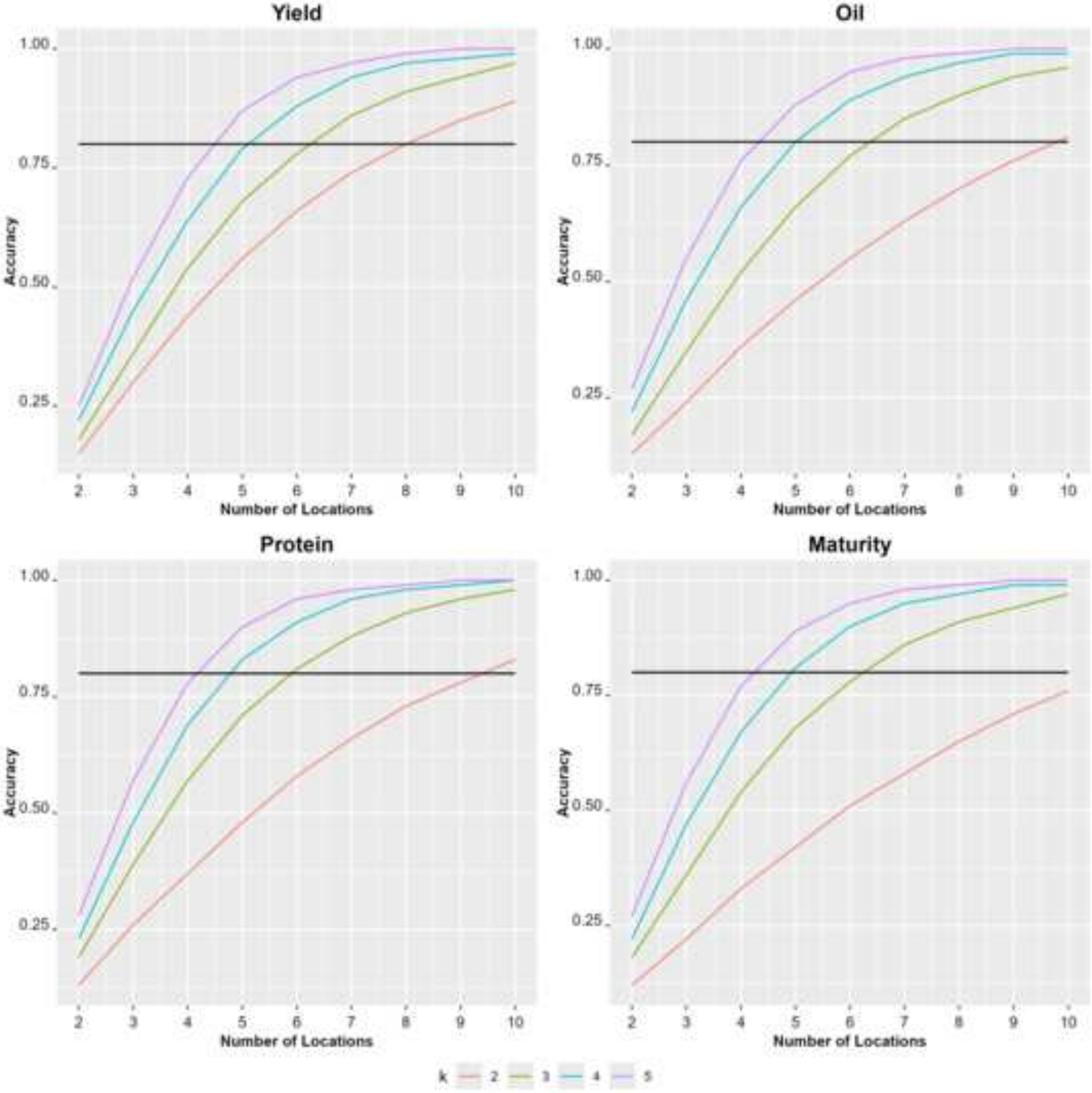

Fig 5

Click here to access/download;Figure;Fig 5.tiff

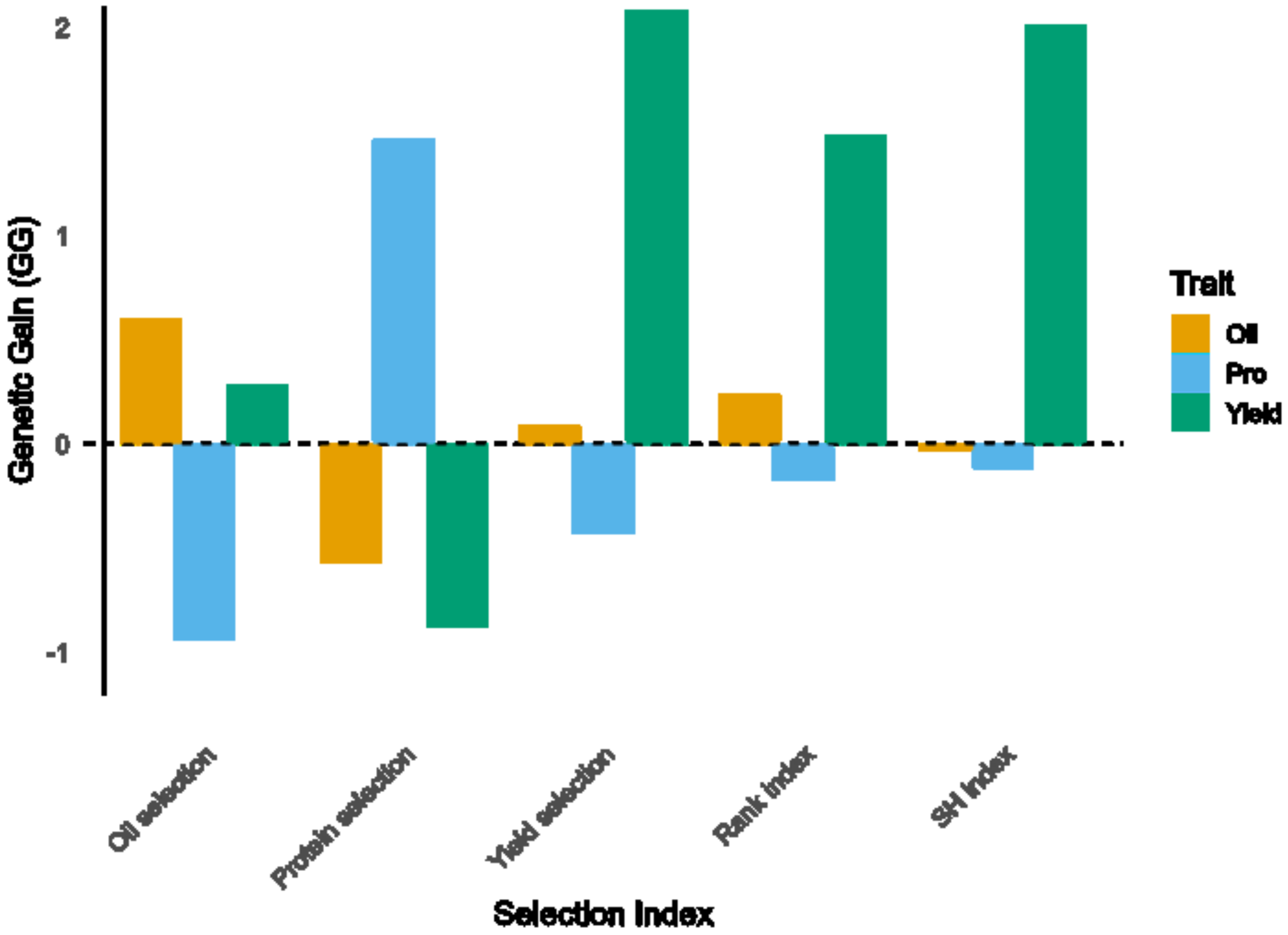

S1 Fig

Click here to access/download
**Supporting Information**
S1 Fig.tiff

S2 Fig

Click here to access/download
**Supporting Information**
S2 Fig.tiff

S3 Fig

Click here to access/download
**Supporting Information**
S3 Fig.tiff

S4 Fig

Click here to access/download
**Supporting Information**
S4 FIg.tiff

S5 Fig

Click here to access/download
**Supporting Information**
S5 Fig.tiff

S6 Fig

Click here to access/download
**Supporting Information**
S6 Fig.tiff

S7 Fig

Click here to access/download
**Supporting Information**
S7 Fig.tiff

S8 Fig

Click here to access/download
**Supporting Information**
S8 Fig.tiff

S9 Fig

Click here to access/download
**Supporting Information**
S9 Fig.tiff

S10 Fig

Click here to access/download
**Supporting Information**
S10 Fig.tiff

S11 Fig

Click here to access/download
**Supporting Information**
S11 Fig.tiff

S12 Fig

Click here to access/download
**Supporting Information**
S12 Fig.tiff

S13 Fig

Click here to access/download
**Supporting Information**
S13 Fig.tiff

S14 Fig

Click here to access/download
**Supporting Information**
S14 Fig.tiff
