# Supplementary material for: Strategies for implementing genomic selection in a public soybean breeding program
Source: PLoS One. 2026 Jul 13;21(7):e0353481. doi: 10.1371/journal.pone.0353481 (PMC13362134; doi:10.1371/journal.pone.0353481)
Supplement: Attachment — Submitted filename: Reviewer comments.docx [file pone.0353481.s022.docx]

Review Comments to the Author

Please use the space provided to explain your answers to the questions above. You may also include additional comments for the author, including concerns about dual publication, research ethics, or publication ethics. (Please upload your review as an attachment if it exceeds 20,000 characters)

**Reviewer #1**: Some points:

Line 147: The model uses Ej for environment and Eijk for error. It may be better to use different letters to avoid confusion.

Done.

Line 150: If Block is treated as a fixed effect, then it should not be assumed that Ej ~ N(0, σ²B).

We changed it.

Line 157: Genomic selection methods: Why not also include the GBLUP method based on the genomic kinship matrix?

The main reason we did not include GBLUP is that several papers show that the prediction accuracy of GBLUP and rrBLUP is similar.

**Reviewer #2**:

Dear Author, I don't have access to the TABLES, neither at the end of the article nor on the submission page. It's difficult to evaluate the manuscript.

The original version included tables; however, it seems there was a technical issue with the journal's interface that made it difficult to access. We have tables in the revised version; hopefully, they are accessible this time.

The objectives, methodology, and discussion are coherent and appropriately presented, and the scientific language is suitable for an academic audience.

We thank the reviewer for the positive assessment of our manuscript.

Line 54: Comment: OK, but what would be the alternative? Inform the reader, and explain why it is not used more frequently.

We rewrote the entire paragraph, bringing the idea that was missing.

Line 118: Write a little about how phenological characteristics can be acquired for genetic selection (conventional and digital methods).

We rewrote the entire paragraph, bringing the idea that was missing.

I don't have access to the TABLES, neither at the end of the article nor on the submission page. It's difficult to evaluate the manuscript.

Please see the comment above. We regret the inconvenience. Tables were included in the original version.

Line 138: What is the exact model and brand?

We have included this information.

Line 144: Isn't 3 standard deviations too much? Reference this.

We have added one of the first papers that explains the methodology.

Line 169: The sentences below appear to be arranged in bullet point format. Is this the most appropriate format for a scientific manuscript? Write in continuous text.

The description of the training population structures has been revised and rewritten in continuous text format in the manuscript.

Line 198: The text is very compartmentalized with small sections of only one sentence. I believe it would be easier to read without these separating structures.

We agree that the previous format made the text overly fragmented. The section describing the statistical parameters has been rewritten in continuous text to improve readability.

Line 263: Almost all figures are missing units. Please insert units where possible.

Units have now been added to all figures where applicable to improve clarity and interpretability.

**Reviewer #3**:

Dear Authors,

The manuscript “Strategies for Implementing Genomic Selection in a Public Soybean Breeding Program” addresses a relevant topic and has the potential to contribute to the improvement of public breeding programs both in the United States and in other regions of the world. The effort to identify efficient and cost-effective genomic selection strategies is particularly valuable given the financial constraints faced by many public breeding programs, especially in developing countries.

Overall, the manuscript presents an interesting approach; however, several aspects require clarification and improvement before the study can fully support its conclusions and provide a clear contribution to the scientific community. My main comments are provided below.

We thank the reviewer for the thoughtful evaluation of our manuscript and for recognizing the relevance of this study to public soybean breeding programs in the United States and internationally. We also appreciate the acknowledgment of the importance of identifying efficient, cost-effective genomic selection strategies, particularly given the financial constraints many public breeding programs face.

We are grateful for the constructive comments and suggestions that have helped improve the manuscript. In response, we have carefully revised the text to clarify methodological aspects, strengthen the interpretation of the results, and improve the presentation of the study’s contributions. We believe that these revisions have enhanced the clarity, robustness, and practical relevance of the work. Detailed responses to each of the reviewer’s comments are provided below.

Introduction
Since the manuscript focuses on public breeding programs, and the dataset appears to originate from such a program, a more detailed description of the breeding scheme would greatly improve the manuscript. Important aspects that could be included are the approximate number of crosses performed per cycle, the selection stages (e.g., early, intermediate, and advanced testing), plot types, number of testing locations at each stage, and the traits evaluated throughout the breeding process. A schematic representation of the breeding pipeline (for example, in a funnel format) could help readers better understand the context of the study.

Some information about the breeding program is provided in Table S1. We have also added more details for the specific breeding program that we highlighted in the paper. At the Iowa State University soybean breeding program, approximately 75 new breeding populations are created each year. Generations were advanced using a modified pod descent method, in which single-plant selection was made in the F2 generation, and a single pod from each selected plant was picked and bulked, which were sent to a winter-season nursery. The returning bulk from the winter nursery is planted in Iowa, and maturity separation, single plant selection for plant height, pod and node placement, and plant health traits are used to identify plants that are grown in a progeny row the following year in a single replication as a paired row plot. However, finer details of the breeding scheme, are considered confidential by the breeding program and therefore cannot be disclosed in the manuscript.

Importantly, the information that cannot be shared does not influence the analyses or results presented in this study. The models and evaluations performed in this work are based solely on the phenotypic and genomic datasets described in the manuscript. Therefore, the absence of these confidential details does not affect the interpretation of the results or the reproducibility of the analyses.

Plant Material

The genetic material used in this study would benefit from a more detailed description. Please clarify the number of genotypes evaluated, their type (e.g., whether they are fully homozygous lines), and the breeding stage from which they were derived.

The number of genotypes per location is explained in the S1 Table.

Experimental Design

I was unable to locate Table 1 in the supplementary materials. This table appears to be missing from the submitted files, which makes it difficult to fully understand the structure of the dataset and the experimental design. Including this information would greatly improve the clarity of the manuscript.

We have included the S1 Table in the Supplementary material.

Molecular Markers

The manuscript currently does not provide information about the molecular markers used in the study. Even if marker characteristics were not part of the evaluation itself, it would be important to include basic information such as marker type, origin of the genotypic data, number of markers used, and criteria for marker filtering or selection.

Information about the markers have been included.

Genomic Selection Methods

The implementation of the genomic selection methods would benefit from additional clarification. Since results are presented by location throughout the manuscript, it seems that adjusted means for each location may have been used to compose the response vector (y) in the models. This point should be clarified explicitly.

We have improved the mixed model to generate the BLUPs used as inputs to the genomic selection models.

In addition, the manuscript appears to use the terms predictive ability and prediction accuracy interchangeably. Prediction accuracy is typically calculated by dividing predictive ability by the square root of heritability, as described in Bernardo (2010, Breeding for Quantitative Traits in Plants, Third Edition). Clarifying this distinction would improve the methodological consistency of the manuscript.

We changed the term 'predictive accuracy' to 'predictive ability', which was what we estimated in our paper.

Optimal Number of Locations to Achieve Maximum Accuracy

The methodology used to determine the optimal number of locations is not entirely clear, and no references are provided. A more detailed description of the analytical approach, along with appropriate references, would help readers understand and evaluate this part of the study.

A reference for this methodology was added.

Selection Index Implementation

The approach used to estimate response to selection based on genomic estimated breeding values could be reconsidered. Since genomic breeding values are already adjusted for heritability, applying heritability again may not be necessary. An alternative approach would be to estimate direct and correlated responses to selection based on selection intensity and selection differentials relative to the population mean. Clarification of this point would strengthen the methodological framework.

You are correct, and we appreciate this important observation. Since genomic estimated breeding values (GEBVs) already incorporate heritability through the genomic prediction model, applying heritability again when estimating the response to selection is not appropriate. We acknowledge that the equation presented in the original manuscript was therefore incorrect.

We have revised the manuscript and corrected the equation to properly reflect the calculation of response to selection based on GEBVs. Importantly, this correction does not affect the results reported in the study, as the analyses were implemented correctly in the R scripts used for the calculations. In other words, the error was limited to the equation description in the manuscript and did not affect the actual computations.

To clarify this point, we now provide the corrected equation in the revised manuscript, and the corresponding R code used for the analysis is shown below.

```
# Define selection intensity (top 10%)
selection_intensity <- 0.1
n_selected <- ceiling(nrow(gebv2) * selection_intensity)
trait_mean<-colMeans(gebv2[,-1])
 gebv3<-gebv2[order(gebv2[[t]], decreasing = TRUE), ]
selected_varieties<-gebv3[1:n_selected,]
selected_mean<-colMeans(selected_varieties[,-1])
SG<-selected_mean-trait_mean
selection_gain[[t]]<-data.frame(Sel_Trait=names(gebv2)[t],t(SG))
```

Methodological Recommendations

The final recommendations regarding the use of RR-BLUP and the population structure strategy based on experimental random selection would benefit from further justification. In particular, it would be helpful to explain more clearly why these approaches are recommended instead of the methods that showed statistically superior performance (e.g., SMV and the genetic algorithm).
We agree that more clearly connecting the numerical results with the final recommendations strengthens the interpretation of the comparative analysis among genomic selection models and training population strategies.

To address this point, we revised the Results and Discussion sections to provide clearer quantitative summaries of model performance across traits and environments. Specifically, we now report the average predictive ability and associated standard errors across environments for each genomic prediction method. These summaries show that although Random Forest (RF) and Support Vector Machine (SVM) occasionally achieved slightly higher predictive ability for certain traits, such as yield, oil, and protein, the differences relative to rrBLUP were generally small and inconsistent across locations and years.

Importantly, more complex machine learning approaches did not consistently outperform rrBLUP across environments, and the magnitude of the observed differences in predictive ability was typically marginal. In contrast, rrBLUP demonstrated highly stable performance across traits, locations, and training population structures, with predictive abilities comparable to those obtained with RF and SVM.

In addition to its competitive predictive performance, rrBLUP offers several practical advantages, including computational efficiency, ease of interpretation, and widespread adoption in plant breeding programs. For these reasons, rrBLUP serves as a robust and practical baseline for genomic prediction in breeding pipelines.

Similarly, although alternative training population optimization approaches, such as the genetic algorithm, sometimes yielded slightly higher predictive performance, the experimental random selection (ERS) strategy consistently achieved high predictive performance across scenarios while maintaining a simpler, more easily implementable structure for breeding programs.

These clarifications have been incorporated into the revised manuscript to emphasize that the recommendations for rrBLUP and ERS are based not only on predictive performance but also on model stability, parsimony, and practical applicability in breeding programs, rather than on marginal numerical differences in predictive accuracy.

Overall, I believe the manuscript addresses an important topic and has good potential for publication after appropriate revisions and clarifications.

We thank the reviewer for the positive evaluation of our manuscript and for recognizing the study's relevance. We appreciate the constructive comments and suggestions provided throughout the review process, which has improved our submission.

Minor Revisions

Lines 95–105 – Since molecular markers were not considered as a factor in this study, dedicating an entire paragraph to this topic in the Introduction seems unnecessary. This section could be shortened or better aligned with the objectives of the study.

We decided to remove this paragraph as we did not evaluate the number of markers as a factor in the paper.

Line 134 – The trait evaluated was described as grain yield, whereas seed yield is the term more commonly used in soybean studies. Please clarify which denomination is most appropriate for the trait evaluated and use consistent terminology throughout the manuscript.

We decided to keep the term 'seed yield' because all the papers we cited used it instead of 'grain yield'.

Line 146 – Please clarify for which factors the Best Linear Unbiased Predictions (BLUPs) were obtained.

We added one sentence before the mixed-model equation in the Materials and Methods to clarify it.

Line 190 – From 10% to 90%, how many classes were considered? The text indicates 10 classes, but this should be clarified to ensure correctness.

We added one sentence in the Training Population Size section of the Materials and Methods to clarify it.

Line 194 – Prediction accuracy is incorrectly defined in this sentence. The correct term appears to be predictive ability (see Bernardo, 2010, p. 268).

We have changed all references to 'prediction accuracy' to 'predictive ability' in the paper.

Line 201 – The definition of prediction accuracy should be revised (see Bernardo, 2010, p. 270).

We have changed all references to 'prediction accuracy' to 'predictive ability' in the paper.

Line 329 – Table 1 – The information presented in Table 1 is not adequately discussed in the text. Additional interpretation would improve the clarity and usefulness of this table.

The main purpose of including Table 1 is to present the information used in the power analysis conducted in this study. Because the results shown in this table primarily support that analysis, we did not include an extensive discussion in the main text. Additionally, a detailed interpretation of these values is not directly related to the manuscript's main objectives. Nevertheless, we agree that additional clarification could improve readability, and we have revised the text to briefly explain the role of Table 1 in the context of the power analysis.

Line 341 – Figure 5 – Figure 5 does not clearly demonstrate an increase in oil content. Please clarify this interpretation or revise the description accordingly.

We thank the reviewer for this observation. We agree that the original description of Figure 5 could lead to the interpretation that oil content increased more clearly than what is directly supported by the figure. To address this issue, we have revised the text to more accurately reflect the patterns shown in the figure. Specifically, we clarified that the selection strategies primarily illustrate the trade-offs among seed yield, oil content, and protein content, rather than a consistent increase in oil content alone. The revised description now focuses on the relative changes among traits and the balance achieved through the selection indices, ensuring that the interpretation is fully consistent with the visual information presented in Figure 5.

**Reviewer #4**:

Generally, the study is comprehensive and well-structured and relevant topic in modern plant breeding landscape. The study presents a systematic analysis of relevant parameters both in breadth and depth and the applications of such analysis in practicable plant breeding programs. The manuscript can be accepted with some revisions, especially clarifying the model used because Gi in line 151 should be random. The author needs to correct Model equation formatting errors, minor typographical errors.

Done.

**Reviewer #5**:

Review of PLOS ONE manuscript PONE-D-26-02573

The PONE-D-26-02573 by Peixoto et al., described a study aimed at developing strategies for implementing genomic selection in public soybean breeding programs. The rationale is clearly stated as well as objectives. The materials and methods are written well but lacking detail in some sections. For example, data collection in field trials is rather cryptic and does not abide by the level of detail required for journal articles of such nature. I have made several comments on the annotated PDF to that effect. In addition, I am sharing the following general comments below.

The selection indices are stated but poorly explained. Authors need to provide more detail on rank-based selection and especially on the sparsely used Smith-Hazel method. How did they calculate the indices in particular? This needs to be described in detail to make a study repeatable independently. None exists currently.

We have included the references for each index method.

For the most part, results are well written and presented. In some cases, a reference is made to certain numbers or percentages, e.g., Figure 1, but the color-coded shades do not show it without a scale being given to read it. This needs to be added.

We agree that the absence of numerical values in Figure 1 could make it difficult for readers to precisely interpret the percentages represented by the color-coded shading. To improve clarity, we have revised Figure 1 to include the corresponding numerical values directly in the figure, enabling readers to easily identify the exact percentages for each color. This modification ensures that the information presented in the figure is interpreted more clearly and consistently with the descriptions in the text.

The population structure, minimum number of individuals and locations, differences between locations for predictive accuracy and strategies around genetic diversity in training panels are the strongest points and contribution of the manuscript.

Thank you for the comment.

Line 134: Plot combine make and model needs to be mentioned.

The make and model of the plot combine have now been added to the manuscript.

Line 135: Which are? Please define as the readers need to know.

We have now clearly defined these items in the manuscript so that readers can easily identify and understand them.

Line 136: This needs to be defines as is in most soybean article, such as R8 (reference) where 95% of the plants are at that stage. A generic statement is not sufficient.

Either define the check cultivars or give a maturity range for them.

We have included information about maturity ratings.

Line 138: Make, model of NIR.

The make and model of the NIR device used for seed composition analysis have now been included in the revised manuscript.

Line 161: It would be helpful to give a reference where the models were first published, or one overall reference for them.

We added one reference for each genomic selection method used in this paper.

Line 238: Authors need to describe how they ranked the traits for the Rank-ased index in detail.

We have included the original reference for the rank index method.

Line 238: Authors need to provide a reference or a brief description of this index, or both. It is not commonly used in the literature.

We have included the original reference for the Smith-Hazel index method.

Line 259: Was this mentioned in the Materials and Methods? If not, it needs to be.

The plateau regression is defined in the first paragraph in the Statistical analysis section in the Materials and Methods.

Line 260: It is impossible to read these numbers in Figure 1 as there is no scale provided in the Figure or its caption, nor is there a color coded scale given for diffrent shades of blue. It must be added, so reader could see the results as stated.

Figure 1 was modified to make all the numbers readable.

Line 323: How was the population partitioned into groups? On what basis? If mentioned elsewhere, it needs to be repeated here in the Results for clarity as well. Fiver different categories are provided in the M & M but which of those made up the three groups here is also important to state.

The population partitioning procedure is described in the Materials and Methods section, where genotypes were grouped into performance-based clusters using the k-means clustering algorithm, resulting in up to five categories: Elite Performers, High Performers, Average Performers, Low Performers, and Underperformers.

To improve clarity, we have now added a brief explanation of this grouping procedure in the Results section, explicitly indicating how the genotypes were partitioned into the groups analyzed in this section. This clarification ensures readers can easily understand the basis of the grouping without referring to the Materials and Methods section.

The revolution of the Figure seems poor. It needs to be improved.

All figures were saved again at 300 dpi, and we use PACE to ensure the highest quality.

Resolution of some Figures seems low and could be improved.

Additional comments are provided in the PDF.

All figures were saved again at 300 dpi, and we use PACE to ensure the highest quality.

Overall, I find the manuscript well written with a potential to advance knowledge on the use of genomic selection in public soybean breeding programs. Pending revision, I would support its publication in PLOS ONE.

We thank the reviewer for the useful comments, which have helped improve the paper.

**Reviewer #6**:

The manuscript presents a technically solid and well-contextualized study, firmly grounded in recent literature and with objectives that are clearly aligned with the needs of public soybean breeding programs, particularly regarding the application of genomic selection to multiple traits and the optimization of training population structure and size. Nonetheless, several aspects of the statistical methodology, interpretation of results, and practical applicability can be refined to further strengthen the work.

We thank the reviewer for the thoughtful evaluation of our manuscript and for recognizing its technical rigor and relevance to public soybean breeding programs. We appreciate the constructive suggestions for improving the statistical methodology, the interpretation of the results, and the discussion of practical implications.

In response to these comments, we have carefully revised the manuscript to clarify the statistical approaches used, improve the interpretation of the results, and expand the discussion of their practical applicability in breeding programs. We provided additional methodological details, improved the connection between the numerical results and the final recommendations, and included further explanations of the optimization of the training population's structure and size, as well as the use of selection indices in breeding decisions.

We believe these revisions have strengthened the clarity, robustness, and practical relevance of the manuscript.

Regarding the mixed model used to obtain BLUPs, the current description contains conceptual and notational inconsistencies. The manuscript states that genotypes were treated as fixed effects, while BLUPs are subsequently used in genomic prediction, which presupposes that genotypes are modeled as random effects. In addition, the notation describing the block and environment effects and their assumed distributions includes what appear to be typographical errors. It would be advisable to explicitly state which effects are fixed and which are random, aligning this choice with the intended inference (e.g., variance components, heritability, and genomic estimated breeding values), and to revise the model notation so that it is internally consistent and coherent with the concept of BLUP.

The model was reviewed, and all the necessary modifications were made.

The criteria adopted for outlier detection, combining boxplot visualization and standardized residuals greater than ±3 standard deviations, are appropriate and commonly used. However, the impact of this filtering step on the data structure and genetic variability could be documented more thoroughly. Presenting the proportion of discarded plots by trait and environment, and briefly commenting on the potential effect of removing extreme genotypes on variance estimates and the distribution of GEBVs, would provide greater transparency and reassure the reader that the data cleaning process did not inadvertently bias the results.

To improve transparency regarding the outlier filtering process, we have now included a detailed summary of the proportion of removed observations by trait, location, and year in Table S2. This table provides a comprehensive overview of the percentage of observations identified as outliers across environments.

In our dataset, the proportion of removed observations was small and relatively consistent across traits and environments, suggesting that the filtering process did not substantially alter the dataset's structure. Outliers were identified based on extreme deviations (±3 standard deviations) and visual inspection using boxplots, which primarily capture observations likely resulting from measurement errors or data inconsistencies rather than biologically meaningful extreme genotypes.

Because the proportion of removed observations was low, the impact on variance components and the distribution of genomic estimated breeding values (GEBVs) is expected to be minimal. Moreover, genomic prediction models are generally robust to the removal of clear data anomalies when these are identified using conservative statistical thresholds. These clarifications have been added to the manuscript to better document the data cleaning procedure and its potential implications.

The cross-validation strategy based on 5-fold partitioning with 10 repetitions is robust from a computational standpoint, but its structure in relation to environments and years is not fully clear. For genomic selection in multi-environment trials, it is crucial to specify whether the folds were constructed within environments (evaluating prediction of unobserved genotypes in observed environments) or whether data from multiple environments were mixed in each fold. This distinction directly affects the interpretation of prediction accuracy and its relevance for predicting new environments or breeding cycles. A more detailed description of how genotypes, environments, and years were stratified (or not) in the cross-validation scheme, along with a short discussion of the implications for extrapolating the results, would substantially improve the methodological clarity.

We have made some modifications to better explain the material and methods to ensure that, for genomic selection, it is clear that analysis was performed for each environment and year separately. There is no analysis considering GxE in the genomic prediction model.

The comparison among genomic selection models is comprehensive, but the narrative could better reconcile the numerical results with the final recommendations. The results sections indicate that Random Forest and Support Vector Machine often yield the highest prediction accuracies for yield, oil, and protein, whereas the conclusions emphasize rrBLUP as the most appropriate approach. This preference is scientifically defensible—simple parametric models are typically more interpretable, computationally efficient, and robust—but the argument would be stronger if supported by quantitative summaries. Presenting average prediction accuracies with standard errors or confidence intervals for each method and trait, and explicitly showing that differences are small or non-significant in most scenarios, would justify the choice of rrBLUP as a practical standard, anchored more in stability and parsimony than in marginal numerical superiority.

We agree that better connecting the numerical results with the final recommendation strengthens the interpretation of the comparative analysis among genomic selection models.

To address this point, we have revised the Results and Discussion sections to provide clearer quantitative summaries of model performance across traits. Specifically, we now report the average predictive ability and associated standard errors across environments for each genomic prediction method. These summaries show that although Random Forest (RF) and Support Vector Machine (SVM) occasionally achieved slightly higher predictive ability for certain traits such as yield, oil, and protein, the differences relative to rrBLUP were generally small and inconsistent across locations and years.

Importantly, rrBLUP showed highly stable performance across environments and traits, with predictive abilities comparable to those of more complex machine learning approaches. Given that rrBLUP is computationally efficient, easier to interpret, and widely adopted in plant breeding programs, we considered it a practical and robust baseline model.

These clarifications have been incorporated into the manuscript to better emphasize that the recommendation of rrBLUP is based not only on predictive performance but also on model stability, parsimony, and practical applicability in breeding pipelines, rather than marginal differences in predictive accuracy.

The implementation details for machine learning methods, particularly Random Forest and SVM, also merit more explicit description. Since these methods are sensitive to hyperparameter tuning, it would be helpful to report which parameter grids were considered, how tuning was performed (e.g., nested within each cross-validation repetition or using a separate procedure), and which performance criteria were used to select optimal configurations. Without these details, readers may question whether the comparison between classical genomic prediction methods and machine learning approaches is fully balanced.

Details on hyperparameter tuning were included in the Materials and Methods.

The use of plateau regression to determine the minimum number of genotypes required in the training population is a valuable contribution, but the statistical characterization of the fitted models is somewhat underdeveloped. Reporting the estimated plateau point alone does not fully convey the reliability of the fitted relationship between training population size and prediction accuracy. Including, at least in supplementary material, the estimated parameters of the plateau models (plateau level, breakpoint), associated standard errors, and goodness-of-fit measures (such as R²) for representative trait–location combinations would allow readers to assess how well the model describes the data and how precise the estimates of minimum training size actually are. In the main text, the current statement that “between 50% and 90% of individuals” are needed is rather broad and could be refined by summarizing more specific ranges by trait or grouping locations with similar behavior.

Thank you for this helpful suggestion. To improve the statistical characterization of the plateau regression models, we have included a new supplementary table (S2 Table) reporting the main parameters of the fitted models, including the estimated breakpoint, plateau accuracy, and goodness-of-fit (R²) for each trait, prediction model, and training population structure. This additional information allows readers to better evaluate how well the plateau models describe the relationship between training population size and predictive ability.

In addition, we revised the main text paragraph to provide a more detailed interpretation of the results by trait, rather than presenting a broad overall range. This revision clarifies how the minimum training population size varies among traits and prediction scenarios, improving the transparency and interpretability of the analysis.

The power analysis used to define the optimal number of locations required to achieve prediction accuracy above 0.80 is conceptually interesting and highly relevant for breeding program design, but its methodological basis would benefit from a clearer exposition. At present, the connection between the variance components reported in the table (genetic, genotype-by-environment, residual variance, and heritability) and the resulting prediction accuracies as a function of the number of locations is only implicit. A more formal description of the statistical model used in the power analysis, including assumptions about replication, the role of genotype clusters, and how the expected correlation between predicted and true genetic values was derived, would make the conclusions more transparent. It would also be valuable to discuss the practical feasibility of deploying the recommended number of locations in public programs and to briefly address possible trade-offs between spatial (number of locations) and temporal (number of years) replication, as well as the potential use of historical data to effectively increase environmental diversity.

We agree that a clearer description of the methodological framework underlying the power analysis improves the transparency of our conclusions. To address this point, we have expanded the Methods section to more explicitly describe how the power analysis was conducted and how the variance components were used to estimate prediction accuracy as a function of the number of locations.

Specifically, we now clarify that the analysis was based on the variance components estimated from the mixed model, including genetic variance, genotype-by-environment interaction variance, and residual variance. These components were used to derive the expected reliability of genotype evaluation across environments, assuming balanced replication across locations. From this framework, we calculated the expected correlation between predicted and true genetic values as a function of the number of testing locations, enabling us to determine the minimum number of environments required to achieve prediction accuracy above the predefined threshold.

We have also clarified the analysis's assumptions, including the use of genotype clusters to represent the population's genetic structure and the assumption of equal replication across locations. These additions make the connection between the estimated variance components and the predicted accuracy more explicit.

In addition, we have expanded the Discussion to address the practical implications of the recommended number of breeding locations. We now note that while increasing the number of testing locations can improve prediction accuracy by capturing greater environmental diversity, breeders often face logistical and budget constraints. Therefore, a balance between spatial replication (number of locations) and temporal replication (number of years) may be necessary. We also highlight that historical multi-environment trial data can effectively increase environmental diversity and help improve prediction accuracy without requiring a proportional increase in new field experiments.

These clarifications have been incorporated into the revised manuscript to better document the assumptions and practical implications of the power analysis.

The section on selection indices is an important link between genomic prediction and actual breeding decisions. The use of both a rank-based index and the Smith–Hazel index is appropriate and well motivated by the known trade-offs among yield, oil, and protein. Nevertheless, the criterion used to define the weights in the Smith–Hazel index—genetic standard deviation of each trait—could be more thoroughly justified. While using genetic standard deviations is a practical approach, it is not the only option, and the manuscript itself highlights the importance and difficulty of defining economic or strategic weights. A brief discussion acknowledging that the chosen weights are a biologically reasonable but somewhat arbitrary compromise, and indicating how the results might change under alternative weighting schemes (for example, prioritizing protein more strongly or incorporating explicit economic values), would add depth to the interpretation. Similarly, specifying the selection intensity used for the calculation of expected genetic gain and quantifying the gains in absolute units (e.g., kg ha⁻¹ for yield, percentage points for oil and protein) would make the results more tangible for breeders.

We agree that additional clarification of the weighting strategy used in the Smith–Hazel index would improve the interpretation of selection index results.

In this study, the weights used in the Smith–Hazel index were based on the genetic standard deviation of each trait, which provides a practical and biologically meaningful way to balance traits measured on different scales while reflecting their underlying genetic variability. However, we acknowledge that this approach represents one of several possible weighting strategies. In breeding programs, index weights are often defined using economic values, breeding priorities, or strategic program objectives, and the optimal choice may vary depending on the target production system and market demands.

To address this point, we have expanded the Discussion section to acknowledge that the weights used in this study represent a practical compromise rather than a unique optimal solution, and that alternative weighting schemes, such as assigning greater importance to protein content or incorporating explicit economic values, could lead to different selection outcomes. We have also clarified the selection intensity used in the calculation of expected genetic gain and reported the gains in absolute trait units to facilitate interpretation from a breeding perspective.

These additions improve the transparency of the selection index methodology and provide a clearer link between genomic prediction results and practical breeding decisions.

From an applied perspective, the manuscript already hints at a set of practical recommendations for public soybean breeding programs, but these could be made more explicit and actionable. A brief, integrative paragraph in the Discussion, summarizing the main operational guidelines—such as adopting rrBLUP as the default genomic prediction model, training with approximately 80% of genotypes per location, constructing the training population using experimental random selection to maintain diversity and representativeness, targeting a minimum number of locations depending on trait complexity and genetic structuring, and relying on a rank-based index for simultaneous improvement of yield and oil while accepting a moderate decrease in protein—would greatly facilitate the translation of the findings into breeding practice.

We agree that making the practical implications of our results more explicit would improve the manuscript's usefulness to breeding programs. Following your recommendation, we have added a new paragraph at the end of the Discussion section summarizing the main operational guidelines derived from our study. This paragraph highlights key recommendations, including the use of rrBLUP as a baseline genomic prediction model, training the model with approximately 80% of the available genotypes, constructing the training population using experimental random selection to maintain genetic diversity, ensuring an adequate number of testing locations depending on trait complexity and population structure, and applying a rank-based selection index to enable simultaneous improvement of multiple traits. We believe this addition clarifies how our results can be applied in practice and enhances the study's translational value for public soybean breeding programs.

Finally, several minor issues related to terminology, notation, and presentation can be refined to improve clarity and consistency. It would be helpful to standardize the terminology for training population structure across the text and figures, to ensure that abbreviations and labels are used in a uniform way. The frequent use of expressions such as “statistically similar” could be accompanied by explicit reference to the statistical test employed and the significance level. In addition, given that the journal strongly encourages open data and reproducibility, it would be preferable to make all analysis scripts and code publicly available in an accessible repository, rather than only “upon request,” and to provide a short schematic overview of the analytical pipeline to help readers follow the sequence from phenotypic data through BLUP estimation, genomic prediction, model comparison, plateau regression, power analysis, and selection index evaluation.

Thank you for these helpful suggestions. We have revised the manuscript to improve clarity and consistency throughout the text and figures. Specifically, we standardized the terminology used to describe the training population structure to ensure that abbreviations and labels are used consistently across the manuscript and figures. In addition, statements referring to results as “statistically similar” have been clarified by explicitly indicating the statistical tests used and the corresponding significance level.

Taken together, addressing these points would not require substantial changes in the core results, but would substantially enhance the statistical rigor, transparency, and practical impact of the study, making the manuscript more compelling both for quantitative geneticists and for breeders interested in implementing genomic selection in real-world public programs.

Thank you for this thoughtful and constructive summary. We appreciate your suggestions and have carefully revised the manuscript to address the points raised throughout the review, which helped improve the overall quality and impact of the manuscript.
