# Supplementary material for: Strategies for implementing genomic selection in a public soybean breeding program
Source: PLoS One. 2026 Jul 13;21(7):e0353481. doi: 10.1371/journal.pone.0353481 (PMC13362134; doi:10.1371/journal.pone.0353481)
Supplement: Peer review history

# Peer review: Decision Letter 0

**DOI:** 10.1371/journal.pone.0353481.r001

**Authors:** Karthikeyan Adhimoolam

3 Mar 2026

PONE-D-26-02573

Strategies for Implementing Genomic Selection in a Public Soybean Breeding Program

PLOS One

Dear Dr. Azevedo Peixoto,

Thank you for submitting your manuscript to PLOS ONE. After careful consideration, we feel that it has merit but does not fully meet PLOS ONE’s publication criteria as it currently stands. Therefore, we invite you to submit a revised version of the manuscript that addresses the points raised during the review process.

Please submit your revised manuscript by Apr 17 2026 11:59PM. If you will need more time than this to complete your revisions, please reply to this message or contact the journal office at plosone@plos.org. When you're ready to submit your revision, log on to https://www.editorialmanager.com/pone/ and select the 'Submissions Needing Revision' folder to locate your manuscript file.

Please include the following items when submitting your revised manuscript:

- A letter that responds to each point raised by the academic editor and reviewer(s). You should upload this letter as a separate file labeled 'Response to Reviewers'.
- A marked-up copy of your manuscript that highlights changes made to the original version. You should upload this as a separate file labeled 'Revised Manuscript with Track Changes'.
- An unmarked version of your revised paper without tracked changes. You should upload this as a separate file labeled 'Manuscript'.

If you would like to make changes to your financial disclosure, please include your updated statement in your cover letter. Guidelines for resubmitting your figure files are available below the reviewer comments at the end of this letter.

If applicable, we recommend that you deposit your laboratory protocols in protocols.io to enhance the reproducibility of your results. Protocols.io assigns your protocol its own identifier (DOI) so that it can be cited independently in the future. For instructions see: https://journals.plos.org/plosone/s/submission-guidelines#loc-laboratory-protocols. Additionally, PLOS ONE offers an option for publishing peer-reviewed Lab Protocol articles, which describe protocols hosted on protocols.io. Read more information on sharing protocols at https://plos.org/protocols?utm_medium=editorial-email&utm_source=authorletters&utm_campaign=protocols.

We look forward to receiving your revised manuscript.

Kind regards,

Karthikeyan Adhimoolam

Academic Editor

PLOS One

Journal Requirements:

When submitting your revision, we need you to address these additional requirements.

1. Please ensure that your manuscript meets PLOS ONE's style requirements, including those for file naming. The PLOS ONE style templates can be found at

https://journals.plos.org/plosone/s/file?id=wjVg/PLOSOne_formatting_sample_main_body.pdf and

https://journals.plos.org/plosone/s/file?id=ba62/PLOSOne_formatting_sample_title_authors_affiliations.pdf

2. In your Methods section, please provide additional information regarding the permits you obtained for the work. Please ensure you have included the full name of the authority that approved the field site access and, if no permits were required, a brief statement explaining why.

3. Please note that PLOS One has specific guidelines on code sharing for submissions in which author-generated code underpins the findings in the manuscript. In these cases, we expect all author-generated code to be made available without restrictions upon publication of the work.

Please review our guidelines at https://journals.plos.org/plosone/s/materials-and-software-sharing#loc-sharing-code and ensure that your code is shared in a way that follows best practice and facilitates reproducibility and reuse.

4. Thank you for stating the following financial disclosure:

“Iowa Soybean Association

R.F. Baker Center for Plant Breeding

Plant Sciences Institute

North Central Soybean Research Program

USDA CRIS project IOW04714

AI Institute for Resilient Agriculture (USDA-NIFA 2021-67021-35329)

G.F. Sprague Chair in Agronomy”

Please state what role the funders took in the study.  If the funders had no role, please state: "The funders had no role in study design, data collection and analysis, decision to publish, or preparation of the manuscript." If this statement is not correct you must amend it as needed.

Please include this amended Role of Funder statement in your cover letter; we will change the online submission form on your behalf.

5. In the online submission form, you indicated that:

“The data underlying the results presented in the study are available from corresponding authors.”

All PLOS journals now require all data underlying the findings described in their manuscript to be freely available to other researchers, either

1. In a public repository,

2. Within the manuscript itself, or

3. Uploaded as supplementary information.

This policy applies to all data except where public deposition would breach compliance with the protocol approved by your research ethics board. If your data cannot be made publicly available for ethical or legal reasons (e.g., public availability would compromise patient privacy), please explain your reasons on resubmission and your exemption request will be escalated for approval.

6. Please upload a copy of Supporting Information S1 Table which you refer to in your text on pages 6 and 33.

7. If the reviewer comments include a recommendation to cite specific previously published works, please review and evaluate these publications to determine whether they are relevant and should be cited. There is no requirement to cite these works unless the editor has indicated otherwise.

**Additional Editor Comments:**

Major revision

[Note: HTML markup is below. Please do not edit.]

Reviewers' comments:

Reviewer's Responses to Questions

**Comments to the Author**

1. Is the manuscript technically sound, and do the data support the conclusions?

The manuscript must describe a technically sound piece of scientific research with data that supports the conclusions. Experiments must have been conducted rigorously, with appropriate controls, replication, and sample sizes. The conclusions must be drawn appropriately based on the data presented.

Reviewer #1: Yes

Reviewer #2: Yes

Reviewer #3: No

Reviewer #4: Yes

Reviewer #5: Yes

Reviewer #6: Yes

**********

2. Has the statistical analysis been performed appropriately and rigorously?

Reviewer #1: Yes

Reviewer #2: Yes

Reviewer #3: No

Reviewer #4: Yes

Reviewer #5: Yes

Reviewer #6: Yes

**********

3. Have the authors made all data underlying the findings in their manuscript fully available?

The PLOS Data policy requires authors to make all data underlying the findings described in their manuscript fully available without restriction, with rare exception (please refer to the Data Availability Statement in the manuscript PDF file). The data should be provided as part of the manuscript or its supporting information, or deposited to a public repository. For example, in addition to summary statistics, the data points behind means, medians and variance measures should be available. If there are restrictions on publicly sharing data—e.g. participant privacy or use of data from a third party—those must be specified.

Reviewer #1: Yes

Reviewer #2: No

Reviewer #3: Yes

Reviewer #4: Yes

Reviewer #5: Yes

Reviewer #6: Yes

**********

4. Is the manuscript presented in an intelligible fashion and written in standard English?

PLOS ONE does not copyedit accepted manuscripts, so the language in submitted articles must be clear, correct, and unambiguous. Any typographical or grammatical errors should be corrected at revision, so please note any specific errors here.

Reviewer #1: Yes

Reviewer #2: Yes

Reviewer #3: Yes

Reviewer #4: Yes

Reviewer #5: Yes

Reviewer #6: Yes

**********

5. Review Comments to the Author

Please use the space provided to explain your answers to the questions above. You may also include additional comments for the author, including concerns about dual publication, research ethics, or publication ethics. (Please upload your review as an attachment if it exceeds 20,000 characters)

Reviewer #1: Some points:

Line 147: The model uses Ej for environment and Eijk for error. It may be better to use different letters to avoid confusion.

Line 150: If Block is treated as a fixed effect, then it should not be assumed that Ej ~ N(0, σ²B).

Line 157: Genomic selection methods: Why not also include the GBLUP method based on the genomic kinship matrix?

Reviewer #2: Dear Author

I don't have access to the TABLES, neither at the end of the article nor on the submission page. It's difficult to evaluate the manuscript.

The objectives, methodology, and discussion are coherent and appropriately presented, and the scientific language is suitable for an academic audience.

Reviewer #3: Dear Authors,

The manuscript “Strategies for Implementing Genomic Selection in a Public Soybean Breeding Program” addresses a relevant topic and has the potential to contribute to the improvement of public breeding programs both in the United States and in other regions of the world. The effort to identify efficient and cost-effective genomic selection strategies is particularly valuable given the financial constraints faced by many public breeding programs, especially in developing countries.

Overall, the manuscript presents an interesting approach; however, several aspects require clarification and improvement before the study can fully support its conclusions and provide a clear contribution to the scientific community. My main comments are provided below.

Introduction

Since the manuscript focuses on public breeding programs, and the dataset appears to originate from such a program, a more detailed description of the breeding scheme would greatly improve the manuscript. Important aspects that could be included are the approximate number of crosses performed per cycle, the selection stages (e.g., early, intermediate, and advanced testing), plot types, number of testing locations at each stage, and the traits evaluated throughout the breeding process. A schematic representation of the breeding pipeline (for example, in a funnel format) could help readers better understand the context of the study.

Plant Material

The genetic material used in this study would benefit from a more detailed description. Please clarify the number of genotypes evaluated, their type (e.g., whether they are fully homozygous lines), and the breeding stage from which they were derived.

Experimental Design

I was unable to locate Table 1 in the supplementary materials. This table appears to be missing from the submitted files, which makes it difficult to fully understand the structure of the dataset and the experimental design. Including this information would greatly improve the clarity of the manuscript.

Molecular Markers

The manuscript currently does not provide information about the molecular markers used in the study. Even if marker characteristics were not part of the evaluation itself, it would be important to include basic information such as marker type, origin of the genotypic data, number of markers used, and criteria for marker filtering or selection.

Genomic Selection Methods

The implementation of the genomic selection methods would benefit from additional clarification. Since results are presented by location throughout the manuscript, it seems that adjusted means for each location may have been used to compose the response vector (y) in the models. This point should be clarified explicitly.

In addition, the manuscript appears to use the terms predictive ability and prediction accuracy interchangeably. Prediction accuracy is typically calculated by dividing predictive ability by the square root of heritability, as described in Bernardo (2010, Breeding for Quantitative Traits in Plants, Third Edition). Clarifying this distinction would improve the methodological consistency of the manuscript.

Optimal Number of Locations to Achieve Maximum Accuracy

The methodology used to determine the optimal number of locations is not entirely clear, and no references are provided. A more detailed description of the analytical approach, along with appropriate references, would help readers understand and evaluate this part of the study.

Selection Index Implementation

The approach used to estimate response to selection based on genomic estimated breeding values could be reconsidered. Since genomic breeding values are already adjusted for heritability, applying heritability again may not be necessary. An alternative approach would be to estimate direct and correlated responses to selection based on selection intensity and selection differentials relative to the population mean. Clarification of this point would strengthen the methodological framework.

Methodological Recommendations

The final recommendations regarding the use of RR-BLUP and the population structure strategy based on experimental random selection would benefit from further justification. In particular, it would be helpful to explain more clearly why these approaches are recommended instead of the methods that showed statistically superior performance (e.g., SMV and the genetic algorithm).

Overall, I believe the manuscript addresses an important topic and has good potential for publication after appropriate revisions and clarifications.

Minor Revisions

Lines 95–105 – Since molecular markers were not considered as a factor in this study, dedicating an entire paragraph to this topic in the Introduction seems unnecessary. This section could be shortened or better aligned with the objectives of the study.

Line 134 – The trait evaluated was described as grain yield, whereas seed yield is the term more commonly used in soybean studies. Please clarify which denomination is most appropriate for the trait evaluated and use consistent terminology throughout the manuscript.

Line 146 – Please clarify for which factors the Best Linear Unbiased Predictions (BLUPs) were obtained.

Line 190 – From 10% to 90%, how many classes were considered? The text indicates 10 classes, but this should be clarified to ensure correctness.

Line 194 – Prediction accuracy is incorrectly defined in this sentence. The correct term appears to be predictive ability (see Bernardo, 2010, p. 268).

Line 201 – The definition of prediction accuracy should be revised (see Bernardo, 2010, p. 270).

Line 329 – Table 1 – The information presented in Table 1 is not adequately discussed in the text. Additional interpretation would improve the clarity and usefulness of this table.

Line 341 – Figure 5 – Figure 5 does not clearly demonstrate an increase in oil content. Please clarify this interpretation or revise the description accordingly.

Reviewer #4: Generally, the study is comprehensive and well-structured and relevant topic in modern plant breeding landscape. The study presents a systematic analysis of relevant parameters both in breadth and depth and the applications of such analysis in practicable plant breeding programs. The manuscript can be accepted with some revisions, especially clarifying the model used because Gi in line 151 should be random. The author needs to correct Model equation formatting errors, minor typographical errors

Reviewer #5: Review of PLOS ONE manuscript PONE-D-26-02573

The PONE-D-26-02573 by Peixoto et al., described a study aimed at developing strategies for implementing genomic selection in public soybean breeding programs. The rationale is clearly stated as well as objectives. The materials and methods are written well but lacking detail in some sections. For example, data collection in field trials is rather cryptic and does not abide by the level of detail required for journal articles of such nature. I have made several comments on the annotated PDF to that effect. In addition, I am sharing the following general comments below.

The selection indices are stated but poorly explained. Authors need to provide more detail on rank-based selection and especially on the sparsely used Smith-Hazel method. How did they calculate the indices in particular? This needs to be described in detail to make a study repeatable independently. None exists currently.

For the most part, results are well written and presented. In some cases, a reference is made to certain numbers or percentages, e.g., Figure 1, but the color-coded shades do not show it without a scale being given to read it. This needs to be added.

The population structure, minimum number of individuals and locations, differences between locations for predictive accuracy and strategies around genetic diversity in training panels are the strongest points and contribution of the manuscript.

Resolution of some Figures seems low and could be improved.

Additional comments are provided in the PDF.

Overall, I find the manuscript well written with a potential to advance knowledge on the use of genomic selection in public soybean breeding programs. Pending revision, I would support its publication in PLOS ONE.

Reviewer #6: The manuscript presents a technically solid and well-contextualized study, firmly grounded in recent literature and with objectives that are clearly aligned with the needs of public soybean breeding programs, particularly regarding the application of genomic selection to multiple traits and the optimization of training population structure and size. Nonetheless, several aspects of the statistical methodology, interpretation of results, and practical applicability can be refined to further strengthen the work.

Regarding the mixed model used to obtain BLUPs, the current description contains conceptual and notational inconsistencies. The manuscript states that genotypes were treated as fixed effects, while BLUPs are subsequently used in genomic prediction, which presupposes that genotypes are modeled as random effects. In addition, the notation describing the block and environment effects and their assumed distributions includes what appear to be typographical errors. It would be advisable to explicitly state which effects are fixed and which are random, aligning this choice with the intended inference (e.g., variance components, heritability, and genomic estimated breeding values), and to revise the model notation so that it is internally consistent and coherent with the concept of BLUP.

The criteria adopted for outlier detection, combining boxplot visualization and standardized residuals greater than ±3 standard deviations, are appropriate and commonly used. However, the impact of this filtering step on the data structure and genetic variability could be documented more thoroughly. Presenting the proportion of discarded plots by trait and environment, and briefly commenting on the potential effect of removing extreme genotypes on variance estimates and the distribution of GEBVs, would provide greater transparency and reassure the reader that the data cleaning process did not inadvertently bias the results.

The cross-validation strategy based on 5-fold partitioning with 10 repetitions is robust from a computational standpoint, but its structure in relation to environments and years is not fully clear. For genomic selection in multi-environment trials, it is crucial to specify whether the folds were constructed within environments (evaluating prediction of unobserved genotypes in observed environments) or whether data from multiple environments were mixed in each fold. This distinction directly affects the interpretation of prediction accuracy and its relevance for predicting new environments or breeding cycles. A more detailed description of how genotypes, environments, and years were stratified (or not) in the cross-validation scheme, along with a short discussion of the implications for extrapolating the results, would substantially improve the methodological clarity.

The comparison among genomic selection models is comprehensive, but the narrative could better reconcile the numerical results with the final recommendations. The results sections indicate that Random Forest and Support Vector Machine often yield the highest prediction accuracies for yield, oil, and protein, whereas the conclusions emphasize rrBLUP as the most appropriate approach. This preference is scientifically defensible—simple parametric models are typically more interpretable, computationally efficient, and robust—but the argument would be stronger if supported by quantitative summaries. Presenting average prediction accuracies with standard errors or confidence intervals for each method and trait, and explicitly showing that differences are small or non-significant in most scenarios, would justify the choice of rrBLUP as a practical standard, anchored more in stability and parsimony than in marginal numerical superiority.

The implementation details for machine learning methods, particularly Random Forest and SVM, also merit more explicit description. Since these methods are sensitive to hyperparameter tuning, it would be helpful to report which parameter grids were considered, how tuning was performed (e.g., nested within each cross-validation repetition or using a separate procedure), and which performance criteria were used to select optimal configurations. Without these details, readers may question whether the comparison between classical genomic prediction methods and machine learning approaches is fully balanced.

The use of plateau regression to determine the minimum number of genotypes required in the training population is a valuable contribution, but the statistical characterization of the fitted models is somewhat underdeveloped. Reporting the estimated plateau point alone does not fully convey the reliability of the fitted relationship between training population size and prediction accuracy. Including, at least in supplementary material, the estimated parameters of the plateau models (plateau level, breakpoint), associated standard errors, and goodness-of-fit measures (such as R²) for representative trait–location combinations would allow readers to assess how well the model describes the data and how precise the estimates of minimum training size actually are. In the main text, the current statement that “between 50% and 90% of individuals” are needed is rather broad and could be refined by summarizing more specific ranges by trait or grouping locations with similar behavior.

The power analysis used to define the optimal number of locations required to achieve prediction accuracy above 0.80 is conceptually interesting and highly relevant for breeding program design, but its methodological basis would benefit from a clearer exposition. At present, the connection between the variance components reported in the table (genetic, genotype-by-environment, residual variance, and heritability) and the resulting prediction accuracies as a function of the number of locations is only implicit. A more formal description of the statistical model used in the power analysis, including assumptions about replication, the role of genotype clusters, and how the expected correlation between predicted and true genetic values was derived, would make the conclusions more transparent. It would also be valuable to discuss the practical feasibility of deploying the recommended number of locations in public programs and to briefly address possible trade-offs between spatial (number of locations) and temporal (number of years) replication, as well as the potential use of historical data to effectively increase environmental diversity.

The section on selection indices is an important link between genomic prediction and actual breeding decisions. The use of both a rank-based index and the Smith–Hazel index is appropriate and well motivated by the known trade-offs among yield, oil, and protein. Nevertheless, the criterion used to define the weights in the Smith–Hazel index—genetic standard deviation of each trait—could be more thoroughly justified. While using genetic standard deviations is a practical approach, it is not the only option, and the manuscript itself highlights the importance and difficulty of defining economic or strategic weights. A brief discussion acknowledging that the chosen weights are a biologically reasonable but somewhat arbitrary compromise, and indicating how the results might change under alternative weighting schemes (for example, prioritizing protein more strongly or incorporating explicit economic values), would add depth to the interpretation. Similarly, specifying the selection intensity used for the calculation of expected genetic gain and quantifying the gains in absolute units (e.g., kg ha⁻¹ for yield, percentage points for oil and protein) would make the results more tangible for breeders.

From an applied perspective, the manuscript already hints at a set of practical recommendations for public soybean breeding programs, but these could be made more explicit and actionable. A brief, integrative paragraph in the Discussion, summarizing the main operational guidelines—such as adopting rrBLUP as the default genomic prediction model, training with approximately 80% of genotypes per location, constructing the training population using experimental random selection to maintain diversity and representativeness, targeting a minimum number of locations depending on trait complexity and genetic structuring, and relying on a rank-based index for simultaneous improvement of yield and oil while accepting a moderate decrease in protein—would greatly facilitate the translation of the findings into breeding practice.

Finally, several minor issues related to terminology, notation, and presentation can be refined to improve clarity and consistency. It would be helpful to standardize the terminology for training population structure across the text and figures, to ensure that abbreviations and labels are used in a uniform way. The frequent use of expressions such as “statistically similar” could be accompanied by explicit reference to the statistical test employed and the significance level. In addition, given that the journal strongly encourages open data and reproducibility, it would be preferable to make all analysis scripts and code publicly available in an accessible repository, rather than only “upon request,” and to provide a short schematic overview of the analytical pipeline to help readers follow the sequence from phenotypic data through BLUP estimation, genomic prediction, model comparison, plateau regression, power analysis, and selection index evaluation.

Taken together, addressing these points would not require substantial changes in the core results, but would substantially enhance the statistical rigor, transparency, and practical impact of the study, making the manuscript more compelling both for quantitative geneticists and for breeders interested in implementing genomic selection in real-world public programs.

**********

6. PLOS authors have the option to publish the peer review history of their article (what does this mean?). If published, this will include your full peer review and any attached files.

If you choose “no”, your identity will remain anonymous but your review may still be made public.

**Do you want your identity to be public for this peer review?**  For information about this choice, including consent withdrawal, please see our Privacy Policy.

Reviewer #1: **Yes:** Bhering, L.L.

Reviewer #2: No

Reviewer #3: **Yes:** Mateus Figueiredo Santos

Reviewer #4: **Yes:** Godfree Chigeza

Reviewer #5: No

Reviewer #6: No

**********

[NOTE: If reviewer comments were submitted as an attachment file, they will be attached to this email and accessible via the submission site. Please log into your account, locate the manuscript record, and check for the action link "View Attachments". If this link does not appear, there are no attachment files.]

To ensure your figures meet our technical requirements, please review our figure guidelines: https://journals.plos.org/plosone/s/figures

You may also use PLOS’s free figure tool, NAAS, to help you prepare publication quality figures: https://journals.plos.org/plosone/s/figures#loc-tools-for-figure-preparation.

NAAS will assess whether your figures meet our technical requirements by comparing each figure against our figure specifications.

Attachment Submitted filename: PONE-D-26-02573_COR.pdf

Attachment Submitted filename: Reviewer comment-GC.pdf

Attachment Submitted filename: PONE-D-26-02573_review.pdf

# Author comment: Author response to Decision Letter 1

**DOI:** 10.1371/journal.pone.0353481.r002

5 May 2026

Review Comments to the Author

Please use the space provided to explain your answers to the questions above. You may also include additional comments for the author, including concerns about dual publication, research ethics, or publication ethics. (Please upload your review as an attachment if it exceeds 20,000 characters)

Reviewer #1: Some points:

Line 147: The model uses Ej for environment and Eijk for error. It may be better to use different letters to avoid confusion.

Done.

Line 150: If Block is treated as a fixed effect, then it should not be assumed that Ej ~ N(0, σ²B).

We changed it.

Line 157: Genomic selection methods: Why not also include the GBLUP method based on the genomic kinship matrix?

The main reason we did not include GBLUP is that several papers show that the prediction accuracy of GBLUP and rrBLUP is similar.

Reviewer #2:

Dear Author, I don't have access to the TABLES, neither at the end of the article nor on the submission page. It's difficult to evaluate the manuscript.

The original version included tables; however, it seems there was a technical issue with the journal's interface that made it difficult to access. We have tables in the revised version; hopefully, they are accessible this time.

The objectives, methodology, and discussion are coherent and appropriately presented, and the scientific language is suitable for an academic audience.

We thank the reviewer for the positive assessment of our manuscript.

Line 54: Comment: OK, but what would be the alternative? Inform the reader, and explain why it is not used more frequently.

We rewrote the entire paragraph, bringing the idea that was missing.

Line 118: Write a little about how phenological characteristics can be acquired for genetic selection (conventional and digital methods).

We rewrote the entire paragraph, bringing the idea that was missing.

I don't have access to the TABLES, neither at the end of the article nor on the submission page. It's difficult to evaluate the manuscript.

Please see the comment above. We regret the inconvenience. Tables were included in the original version.

Line 138: What is the exact model and brand?

We have included this information.

Line 144: Isn't 3 standard deviations too much? Reference this.

We have added one of the first papers that explains the methodology.

Line 169: The sentences below appear to be arranged in bullet point format. Is this the most appropriate format for a scientific manuscript? Write in continuous text.

The description of the training population structures has been revised and rewritten in continuous text format in the manuscript.

Line 198: The text is very compartmentalized with small sections of only one sentence. I believe it would be easier to read without these separating structures.

We agree that the previous format made the text overly fragmented. The section describing the statistical parameters has been rewritten in continuous text to improve readability.

Line 263: Almost all figures are missing units. Please insert units where possible.

Units have now been added to all figures where applicable to improve clarity and interpretability.

Reviewer #3:

Dear Authors,

The manuscript “Strategies for Implementing Genomic Selection in a Public Soybean Breeding Program” addresses a relevant topic and has the potential to contribute to the improvement of public breeding programs both in the United States and in other regions of the world. The effort to identify efficient and cost-effective genomic selection strategies is particularly valuable given the financial constraints faced by many public breeding programs, especially in developing countries.

Overall, the manuscript presents an interesting approach; however, several aspects require clarification and improvement before the study can fully support its conclusions and provide a clear contribution to the scientific community. My main comments are provided below.

We thank the reviewer for the thoughtful evaluation of our manuscript and for recognizing the relevance of this study to public soybean breeding programs in the United States and internationally. We also appreciate the acknowledgment of the importance of identifying efficient, cost-effective genomic selection strategies, particularly given the financial constraints many public breeding programs face.

We are grateful for the constructive comments and suggestions that have helped improve the manuscript. In response, we have carefully revised the text to clarify methodological aspects, strengthen the interpretation of the results, and improve the presentation of the study’s contributions. We believe that these revisions have enhanced the clarity, robustness, and practical relevance of the work. Detailed responses to each of the reviewer’s comments are provided below.

Introduction

Since the manuscript focuses on public breeding programs, and the dataset appears to originate from such a program, a more detailed description of the breeding scheme would greatly improve the manuscript. Important aspects that could be included are the approximate number of crosses performed per cycle, the selection stages (e.g., early, intermediate, and advanced testing), plot types, number of testing locations at each stage, and the traits evaluated throughout the breeding process. A schematic representation of the breeding pipeline (for example, in a funnel format) could help readers better understand the context of the study.

Some information about the breeding program is provided in Table S1. We have also added more details for the specific breeding program that we highlighted in the paper. At the Iowa State University soybean breeding program, approximately 75 new breeding populations are created each year. Generations were advanced using a modified pod descent method, in which single-plant selection was made in the F2 generation, and a single pod from each selected plant was picked and bulked, which were sent to a winter-season nursery. The returning bulk from the winter nursery is planted in Iowa, and maturity separation, single plant selection for plant height, pod and node placement, and plant health traits are used to identify plants that are grown in a progeny row the following year in a single replication as a paired row plot. However, finer details of the breeding scheme, are considered confidential by the breeding program and therefore cannot be disclosed in the manuscript.

Importantly, the information that cannot be shared does not influence the analyses or results presented in this study. The models and evaluations performed in this work are based solely on the phenotypic and genomic datasets described in the manuscript. Therefore, the absence of these confidential details does not affect the interpretation of the results or the reproducibility of the analyses.

Plant Material

The genetic material used in this study would benefit from a more detailed description. Please clarify the number of genotypes evaluated, their type (e.g., whether they are fully homozygous lines), and the breeding stage from which they were derived.

The number of genotypes per location is explained in the S1 Table.

Experimental Design

I was unable to locate Table 1 in the supplementary materials. This table appears to be missing from the submitted files, which makes it difficult to fully understand the structure of the dataset and the experimental design. Including this information would greatly improve the clarity of the manuscript.

We have included the S1 Table in the Supplementary material.

Molecular Markers

The manuscript currently does not provide information about the molecular markers used in the study. Even if marker characteristics were not part of the evaluation itself, it would be important to include basic information such as marker type, origin of the genotypic data, number of markers used, and criteria for marker filtering or selection.

Information about the markers have been included.

Genomic Selection Methods

The implementation of the genomic selection methods would benefit from additional clarification. Since results are presented by location throughout the manuscript, it seems that adjusted means for each location may have been used to compose the response vector (y) in the models. This point should be clarified explicitly.

We have improved the mixed model to generate the BLUPs used as inputs to the genomic selection models.

In addition, the manuscript appears to use the terms predictive ability and prediction accuracy interchangeably. Prediction accuracy is typically calculated by dividing predictive ability by the square root of heritability, as described in Bernardo (2010, Breeding for Quantitative Traits in Plants, Third Edition). Clarifying this distinction would improve the methodological consistency of the manuscript.

We changed the term 'predictive accuracy' to 'predictive ability', which was what we estimated in our paper.

Optimal Number of Locations to Achieve Maximum Accuracy

The methodology used to determine the optimal number of locations is not entirely clear, and no references are provided. A more detailed description of the analytical approach, along with appropriate references, would help readers understand and evaluate this part of the study.

A reference for this methodology was added.

Selection Index Implementation

The approach used to estimate response to selection based on genomic estimated breeding values could be reconsidered. Since genomic breeding values are already adjusted for heritability, applying heritability again may not be necessary. An alternative approach would be to estimate direct and correlated responses to selection based on selection intensity and selection differentials relative to the population mean. Clarification of this point would strengthen the methodological framework.

You are correct, and we appreciate this important observation. Since genomic estimated breeding values (GEBVs) already incorporate heritability through the genomic prediction model, applying heritability again when estimating the response to selection is not appropriate. We acknowledge that the equation presented in the original manuscript was therefore incorrect.

We have revised the manuscript and corrected the equation to properly reflect the calculation of response to selection based on GEBVs. Importantly, this correction does not affect the results reported in the study, as the analyses were implemented correctly in the R scripts used for the calculations. In other words, the error was limited to the equation description in the manuscript and did not affect the actual computations.

To clarify this point, we now provide the corrected equation in the revised manuscript, and the corresponding R code used for the analysis is shown below.

# Define selection intensity (top 10%)

selection_intensity <- 0.1

n_selected <- ceiling(nrow(gebv2) * selection_intensity)

trait_mean<-colMeans(gebv2[,-1])

gebv3<-gebv2[order(gebv2[[t]], decreasing = TRUE), ]

selected_varieties<-gebv3[1:n_selected,]

selected_mean<-colMeans(selected_varieties[,-1])

SG<-selected_mean-trait_mean

selection_gain[[t]]<-data.frame(Sel_Trait=names(gebv2)[t],t(SG))

Methodological Recommendations

The final recommendations regarding the use of RR-BLUP and the population structure strategy based on experimental random selection would benefit from further justification. In particular, it would be helpful to explain more clearly why these approaches are recommended instead of the methods that showed statistically superior performance (e.g., SMV and the genetic algorithm).

We agree that more clearly connecting the numerical results with the final recommendations strengthens the interpretation of the comparative analysis among genomic selection models and training population strategies.

To address this point, we revised the Results and Discussion sections to provide clearer quantitative summaries of model performance across traits and environments. Specifically, we now report the average predictive ability and associated standard errors across environments for each genomic prediction method. These summaries show that although Random Forest (RF) and Support Vector Machine (SVM) occasionally achieved slightly higher predictive ability for certain traits, such as yield, oil, and protein, the differences relative to rrBLUP were generally small and inconsistent across locations and years.

Importantly, more complex machine learning approaches did not consistently outperform rrBLUP across environments, and the magnitude of the observed differences in predictive ability was typically marginal. In contrast, rrBLUP demonstrated highly stable performance across traits, locations, and training population structures, with predictive abilities comparable to those obtained with RF and SVM.

In addition to its competitive predictive performance, rrBLUP offers several practical advantages, including computational efficiency, ease of interpretation, and widespread adoption in plant breeding programs. For these reasons, rrBLUP serves as a robust and practical baseline for genomic prediction in breeding pipelines.

Similarly, although alternative training population optimization approaches, such as the genetic algorithm, sometimes yielded slightly higher predictive performance, the experimental random selection (ERS) strategy consistently achieved high predictive performance across scenarios while maintaining a simpler, more easily implementable structure for breeding programs.

These clarifications have been incorporated into the revised manuscript to emphasize that the recommendations for rrBLUP and ERS are based not only on predictive performance but also on model stability, parsimony, and practical applicability in breeding programs, rather than on marginal numerical differences in predictive accuracy.

Overall, I believe the manuscript addresses an important topic and has good potential for publication after appropriate revisions and clarifications.

We thank the reviewer for the positive evaluation of our manuscript and for recognizing the study's relevance. We appreciate the constructive comments and suggestions provided throughout the review process, which has improved our submission.

Minor Revisions

Lines 95–105 – Since molecular markers were not considered as a factor in this study, dedicating an entire paragraph to this topic in the Introduction seems unnecessary. This section could be shortened or better aligned with the objectives of the study.

We decided to remove this paragraph as we did not evaluate the number of markers as a factor in the paper.

Line 134 – The trait evaluated was described as grain yield, whereas seed yield is the term more commonly used in soybean studies. Please clarify which denomination is most appropriate for the trait evaluated and use consistent terminology throughout the manuscript.

We decided to keep the term 'seed yield' because all the papers we cited used it instead of 'grain yield'.

Line 146 – Please clarify for which factors the Best Linear Unbiased Predictions (BLUPs) were obtained.

We added one sentence before the mixed-model equation in the Materials and Methods to clarify it.

Line 190 – From 10% to 90%, how many classes were considered? The text indicates 10 classes, but this should be clarified to ensure correctness.

We added one sentence in the Training Population Size section of the Materials and Methods to clarify it.

Line 194 – Prediction accuracy is incorrectly defined in this sentence. The correct term appears to be predictive ability (see Bernardo, 2010, p. 268).

We have changed all references to 'prediction accuracy' to 'predictive ability' in the paper.

Line 201 – The definition of prediction accuracy should be revised (see Bernardo, 2010, p. 270).

We have changed all references to 'prediction accuracy' to 'predictive ability' in the paper.

Line 329 – Table 1 – The information presented in Table 1 is not adequately discussed in the text. Additional interpretation would improve the clarity and usefulness of this table.

The main purpose of including Table 1 is to present the information used in the power analysis conducted in this study. Because the results shown in this tab

Attachment Submitted filename: Reviewer comments.docx

# Peer review: Decision Letter 1

**DOI:** 10.1371/journal.pone.0353481.r003

**Authors:** Karthikeyan Adhimoolam, Karthikeyan Adhimoolam

24 Jun 2026

<p>Strategies for Implementing Genomic Selection in a Public Soybean Breeding Program

PONE-D-26-02573R1

Dear Dr. Leonardo,

We’re pleased to inform you that your manuscript has been judged scientifically suitable for publication and will be formally accepted for publication once it meets all outstanding technical requirements.

Within one week, you’ll receive an e-mail detailing the required amendments. When these have been addressed, you’ll receive a formal acceptance letter and your manuscript will be scheduled for publication.

An invoice will be generated when your article is formally accepted. Please note, if your institution has a publishing partnership with PLOS and your article meets the relevant criteria, all or part of your publication costs will be covered. Please make sure your user information is up-to-date by logging into Editorial Manager at Editorial Manager® and clicking the ‘Update My Information' link at the top of the page. For questions related to billing, please contact billing support.

If your institution or institutions have a press office, please notify them about your upcoming paper to help maximize its impact. If they’ll be preparing press materials, please inform our press team as soon as possible -- no later than 48 hours after receiving the formal acceptance. Your manuscript will remain under strict press embargo until 2 pm Eastern Time on the date of publication. For more information, please contact onepress@plos.org.

Kind regards,

Karthikeyan Adhimoolam

Academic Editor

PLOS One

Additional Editor Comments (optional):

Reviewers' comments:

Reviewer's Responses to Questions

**Comments to the Author**

1. If the authors have adequately addressed your comments raised in a previous round of review and you feel that this manuscript is now acceptable for publication, you may indicate that here to bypass the “Comments to the Author” section, enter your conflict of interest statement in the “Confidential to Editor” section, and submit your "Accept" recommendation.

Reviewer #3: All comments have been addressed

Reviewer #5: All comments have been addressed

Reviewer #6: All comments have been addressed

**********

2. Is the manuscript technically sound, and do the data support the conclusions?

The manuscript must describe a technically sound piece of scientific research with data that supports the conclusions. Experiments must have been conducted rigorously, with appropriate controls, replication, and sample sizes. The conclusions must be drawn appropriately based on the data presented.

Reviewer #3: Yes

Reviewer #5: Yes

Reviewer #6: Yes

**********

3. Has the statistical analysis been performed appropriately and rigorously?

Reviewer #3: Yes

Reviewer #5: Yes

Reviewer #6: Yes

**********

4. Have the authors made all data underlying the findings in their manuscript fully available?

The PLOS Data policy requires authors to make all data underlying the findings described in their manuscript fully available without restriction, with rare exception (please refer to the Data Availability Statement in the manuscript PDF file). The data should be provided as part of the manuscript or its supporting information, or deposited to a public repository. For example, in addition to summary statistics, the data points behind means, medians and variance measures should be available. If there are restrictions on publicly sharing data—e.g. participant privacy or use of data from a third party—those must be specified.

Reviewer #3: Yes

Reviewer #5: Yes

Reviewer #6: Yes

**********

5. Is the manuscript presented in an intelligible fashion and written in standard English?

PLOS ONE does not copyedit accepted manuscripts, so the language in submitted articles must be clear, correct, and unambiguous. Any typographical or grammatical errors should be corrected at revision, so please note any specific errors here.

Reviewer #3: Yes

Reviewer #5: Yes

Reviewer #6: Yes

**********

6. Review Comments to the Author

Please use the space provided to explain your answers to the questions above. You may also include additional comments for the author, including concerns about dual publication, research ethics, or publication ethics. (Please upload your review as an attachment if it exceeds 20,000 characters)

Reviewer #3: Dear authors,

The manuscript is now ready for publication.

One minor revision: include the titles of the tables in supplementary material.

Reviewer #5: Dear Authors,

I believe that you have addressed all reviewers' comments adequately. Hence, I suggest that the manuscript be accepted for publication in PLOS ONE. Thank you.

Reviewer #6: The authors have made the corrections I requested, and the manuscript can be accepted for publication.

**********

7. PLOS authors have the option to publish the peer review history of their article (what does this mean?). If published, this will include your full peer review and any attached files.

If you choose “no”, your identity will remain anonymous but your review may still be made public.

**Do you want your identity to be public for this peer review?**  For information about this choice, including consent withdrawal, please see our Privacy Policy.

Reviewer #3: No

Reviewer #5: No

Reviewer #6: No

**********

# Editor report: Acceptance letter

**DOI:** 10.1371/journal.pone.0353481.r004

**Authors:** Karthikeyan Adhimoolam, Karthikeyan Adhimoolam

PONE-D-26-02573R1

PLOS One

Dear Dr. Azevedo Peixoto,

I'm pleased to inform you that your manuscript has been deemed suitable for publication in PLOS One. Congratulations! Your manuscript is now being handed over to our production team.

At this stage, our production department will prepare your paper for publication. This includes ensuring the following:

* All references, tables, and figures are properly cited

* All relevant supporting information is included in the manuscript submission,

* There are no issues that prevent the paper from being properly typeset

You will receive further instructions from the production team, including instructions on how to review your proof when it is ready. Please keep in mind that we are working through a large volume of accepted articles, so please give us a few days to review your paper and let you know the next and final steps.

Lastly, if your institution or institutions have a press office, please let them know about your upcoming paper now to help maximize its impact. If they'll be preparing press materials, please inform our press team within the next 48 hours. Your manuscript will remain under strict press embargo until 2 pm Eastern Time on the date of publication. For more information, please contact onepress@plos.org.

You will receive an invoice from PLOS for your publication fee after your manuscript has reached the completed accept phase. If you receive an email requesting payment before acceptance or for any other service, this may be a phishing scheme. Learn how to identify phishing emails and protect your accounts at https://explore.plos.org/phishing.

If we can help with anything else, please email us at customercare@plos.org.

Thank you for submitting your work to PLOS ONE and supporting open access.

Kind regards,

PLOS ONE Editorial Office Staff

on behalf of

Dr. Karthikeyan Adhimoolam

Academic Editor

PLOS One
